# Methyltransferase TaSAMT1 mediates wheat freezing tolerance by integrating brassinosteroid and salicylic acid signaling

**DOI:** 10.1093/plcell/koae100

**Published:** 2024-03-27

**Authors:** Wei Chu, Shumin Chang, Jingchen Lin, Chenji Zhang, Jinpeng Li, Xingbei Liu, Zehui Liu, Debiao Liu, Qun Yang, Danyang Zhao, Xiaoyu Liu, Weilong Guo, Mingming Xin, Yingyin Yao, Huiru Peng, Chaojie Xie, Zhongfu Ni, Qixin Sun, Zhaorong Hu

**Affiliations:** Frontiers Science Center for Molecular Design Breeding/Key Laboratory of Crop Heterosis and Utilization (MOE)/Beijing Key Laboratory of Crop Genetic Improvement, China Agricultural University, No. 2 Yuanmingyuan Xi Road, Haidian District, Beijing 100193, PR China; Frontiers Science Center for Molecular Design Breeding/Key Laboratory of Crop Heterosis and Utilization (MOE)/Beijing Key Laboratory of Crop Genetic Improvement, China Agricultural University, No. 2 Yuanmingyuan Xi Road, Haidian District, Beijing 100193, PR China; Frontiers Science Center for Molecular Design Breeding/Key Laboratory of Crop Heterosis and Utilization (MOE)/Beijing Key Laboratory of Crop Genetic Improvement, China Agricultural University, No. 2 Yuanmingyuan Xi Road, Haidian District, Beijing 100193, PR China; Frontiers Science Center for Molecular Design Breeding/Key Laboratory of Crop Heterosis and Utilization (MOE)/Beijing Key Laboratory of Crop Genetic Improvement, China Agricultural University, No. 2 Yuanmingyuan Xi Road, Haidian District, Beijing 100193, PR China; Frontiers Science Center for Molecular Design Breeding/Key Laboratory of Crop Heterosis and Utilization (MOE)/Beijing Key Laboratory of Crop Genetic Improvement, China Agricultural University, No. 2 Yuanmingyuan Xi Road, Haidian District, Beijing 100193, PR China; Frontiers Science Center for Molecular Design Breeding/Key Laboratory of Crop Heterosis and Utilization (MOE)/Beijing Key Laboratory of Crop Genetic Improvement, China Agricultural University, No. 2 Yuanmingyuan Xi Road, Haidian District, Beijing 100193, PR China; Frontiers Science Center for Molecular Design Breeding/Key Laboratory of Crop Heterosis and Utilization (MOE)/Beijing Key Laboratory of Crop Genetic Improvement, China Agricultural University, No. 2 Yuanmingyuan Xi Road, Haidian District, Beijing 100193, PR China; Frontiers Science Center for Molecular Design Breeding/Key Laboratory of Crop Heterosis and Utilization (MOE)/Beijing Key Laboratory of Crop Genetic Improvement, China Agricultural University, No. 2 Yuanmingyuan Xi Road, Haidian District, Beijing 100193, PR China; Frontiers Science Center for Molecular Design Breeding/Key Laboratory of Crop Heterosis and Utilization (MOE)/Beijing Key Laboratory of Crop Genetic Improvement, China Agricultural University, No. 2 Yuanmingyuan Xi Road, Haidian District, Beijing 100193, PR China; Frontiers Science Center for Molecular Design Breeding/Key Laboratory of Crop Heterosis and Utilization (MOE)/Beijing Key Laboratory of Crop Genetic Improvement, China Agricultural University, No. 2 Yuanmingyuan Xi Road, Haidian District, Beijing 100193, PR China; Frontiers Science Center for Molecular Design Breeding/Key Laboratory of Crop Heterosis and Utilization (MOE)/Beijing Key Laboratory of Crop Genetic Improvement, China Agricultural University, No. 2 Yuanmingyuan Xi Road, Haidian District, Beijing 100193, PR China; Frontiers Science Center for Molecular Design Breeding/Key Laboratory of Crop Heterosis and Utilization (MOE)/Beijing Key Laboratory of Crop Genetic Improvement, China Agricultural University, No. 2 Yuanmingyuan Xi Road, Haidian District, Beijing 100193, PR China; Frontiers Science Center for Molecular Design Breeding/Key Laboratory of Crop Heterosis and Utilization (MOE)/Beijing Key Laboratory of Crop Genetic Improvement, China Agricultural University, No. 2 Yuanmingyuan Xi Road, Haidian District, Beijing 100193, PR China; Frontiers Science Center for Molecular Design Breeding/Key Laboratory of Crop Heterosis and Utilization (MOE)/Beijing Key Laboratory of Crop Genetic Improvement, China Agricultural University, No. 2 Yuanmingyuan Xi Road, Haidian District, Beijing 100193, PR China; Frontiers Science Center for Molecular Design Breeding/Key Laboratory of Crop Heterosis and Utilization (MOE)/Beijing Key Laboratory of Crop Genetic Improvement, China Agricultural University, No. 2 Yuanmingyuan Xi Road, Haidian District, Beijing 100193, PR China; Frontiers Science Center for Molecular Design Breeding/Key Laboratory of Crop Heterosis and Utilization (MOE)/Beijing Key Laboratory of Crop Genetic Improvement, China Agricultural University, No. 2 Yuanmingyuan Xi Road, Haidian District, Beijing 100193, PR China; Frontiers Science Center for Molecular Design Breeding/Key Laboratory of Crop Heterosis and Utilization (MOE)/Beijing Key Laboratory of Crop Genetic Improvement, China Agricultural University, No. 2 Yuanmingyuan Xi Road, Haidian District, Beijing 100193, PR China; Frontiers Science Center for Molecular Design Breeding/Key Laboratory of Crop Heterosis and Utilization (MOE)/Beijing Key Laboratory of Crop Genetic Improvement, China Agricultural University, No. 2 Yuanmingyuan Xi Road, Haidian District, Beijing 100193, PR China; Frontiers Science Center for Molecular Design Breeding/Key Laboratory of Crop Heterosis and Utilization (MOE)/Beijing Key Laboratory of Crop Genetic Improvement, China Agricultural University, No. 2 Yuanmingyuan Xi Road, Haidian District, Beijing 100193, PR China

## Abstract

Cold injury is a major environmental stress affecting the growth and yield of crops. Brassinosteroids (BRs) and salicylic acid (SA) play important roles in plant cold tolerance. However, whether or how BR signaling interacts with the SA signaling pathway in response to cold stress is still unknown. Here, we identified an SA methyltransferase, TaSAMT1 that converts SA to methyl SA (MeSA) and confers freezing tolerance in wheat (*Triticum aestivum*). *TaSAMT1* overexpression greatly enhanced wheat freezing tolerance, with plants accumulating more MeSA and less SA, whereas *Tasamt1* knockout lines were sensitive to freezing stress and accumulated less MeSA and more SA. Spraying plants with MeSA conferred freezing tolerance to *Tasamt1* mutants, but SA did not. We revealed that BRASSINAZOLE-RESISTANT 1 (TaBZR1) directly binds to the *TaSAMT1* promoter and induces its transcription. Moreover, TaBZR1 interacts with the histone acetyltransferase TaHAG1, which potentiates *TaSAMT1* expression via increased histone acetylation and modulates the SA pathway during freezing stress. Additionally, overexpression of *TaBZR1* or *TaHAG1* altered *TaSAMT1* expression and improved freezing tolerance. Our results demonstrate a key regulatory node that connects the BR and SA pathways in the plant cold stress response. The regulatory factors or genes identified could be effective targets for the genetic improvement of freezing tolerance in crops.

## Teaser

Transcription factor TaBZR1 directly activates methyltransferase-encoding *TaSAMT1-B* under freezing stress by recruiting a histone acetyltransferase and increasing its histone H3 acetylation.

## Introduction

Cold stress severely limits crop quality and productivity. As one of the most important staple crops worldwide, bread wheat (*T. aestivum*) provides approximately 20% of the daily dietary calories and protein (Shewry and Hey 2015; FAOSTAT 2020). As with other crops, climate-driven temperature extremes impair the vegetative and reproductive growth of wheat, resulting in lower yield (Thakur et al. 2010; Frederiks et al. 2015). Periods of cold are common in wheat-growing regions throughout the world, including Australia, China, Europe, and the United States (Holman et al. 2011; Trnka et al. 2014; Zheng et al. 2015; Crimp et al. 2016; Jiang et al. 2022). Therefore, understanding the molecular mechanisms underlying cold tolerance has become a high priority for research and wheat breeding programs.

Phytohormones influence diverse events associated with plant growth and tolerance under cold stress by dynamically modulating physiological, biochemical, and molecular responses through stress-responsive regulatory cascades (Eremina et al. 2016a; Raza et al. 2023). Abscisic acid (ABA), brassinosteroid (BR), ethylene, jasmonic acid (JA), and gibberellic acid (GA) signaling play important roles in regulating plant freezing tolerance via pathways that depend on or are independent of the C-REPEAT BINDING FACTOR (CBF) transcription factors (Hu et al. 2013; Venzhik et al. 2016; Li et al. 2017; Ye et al. 2019; Lantzouni et al. 2020; Yu et al. 2020; Huang et al. 2023).

In addition, molecular and physiological responses to cold stress are intricately linked to the regulation of phytohormones (Verma et al. 2016; Waadt et al. 2022). Low temperatures influence phytohormone pathways by activating or suppressing key molecular hubs in plant hormone biosynthesis, homeostasis, signaling, and downstream responses (Ding and Yang 2022). These regulatory steps typically depend on transcriptional, post-transcriptional, and posttranslational mechanisms, some of which have been directly connected to canonical CBF signaling (Ye et al. 2019; Jiang et al. 2020; Castroverde and Dina 2021). Moreover, crosstalk among plant hormones is important for the transcriptional activation of downstream genes essential for plant development and responses to stress. For example, JASMONATE-ZIM DOMAIN PROTEINs (JAZs) interact with ETHYLENE-INSENSITIVE3 (EIN3) and the related EIN3-LIKE1 (EIL1) to mediate JA, ethylene, and CBF signaling (Hu et al. 2013; Li et al. 2012; Shi et al. 2012, 2015). Likewise, regulation of DELLAs by PHYTOCHROME-INTERACTING FACTOR4 (PIF4)–BRASSINAZOLE-RESISTANT 1 (BZR1) modulates GA, BR, and CBF signaling pathways (Achard et al. 2008; Oh et al. 2012; Shi et al. 2015; Wang et al. 2020). However, the mechanisms underlying the crosstalk among different phytohormone signaling cascades remain to be investigated in the regulation of freezing tolerance.

Salicylic acid (SA), a small-molecule plant hormone that regulates plant development and plant responses to pathogen attack, also appears to be involved in cold tolerance (Siboza et al. 2014). Exogenous SA treatment mitigates freezing injury in the leaves of maize (*Zea mays*) and wheat plants grown under low-temperature conditions (Kang and Saltveit 2002; Taşgín et al. 2003). Treatment with SA also diminished cold injury symptoms in several horticultural crops (Ding et al. 2002; Wang et al. 2006; Sayyari et al. 2009). Notably, high concentrations of SA and the continuous application of SA impose severe growth damage and decreased cold tolerance capacity, suggesting that timely application of SA may enhance cold tolerance, whereas continuous SA application may lead to the opposite effect (Scott et al. 2004; Miura and Tada 2014; Nadarajah et al. 2021). Moreover, methyl SA (MeSA) enhances cold tolerance in various species, including Arabidopsis (*Arabidopsis thaliana*), tomato (*Solanum lycopersicum*), wheat, and several fruit trees (Sayyari et al. 2011; Zhang et al. 2011; Siboza et al. 2014; Habibi et al. 2019).

SA was initially postulated to be a mobile signal in defense signaling pathways and required for systemic acquired resistance (SAR) (Gaffney et al. 1993; Durrant and Dong 2004), despite grafting studies suggesting that SA maybe not be a translocated mobile signal during SAR (Vernooij et al. 1994). Recent reports have shown that translocation of SA from primary infected tissue to distal tissues likely occurs via the apoplast and is regulated by leaf transpiration (Lim et al. 2016, 2020). MeSA is a volatile form of SA but can be also transported (Attaran et al. 2009). Intriguingly, MeSA also acts as a critical mobile signal and is assembled via the phloem to stimulate SAR (Park et al. 2007; Klessig et al. 2018). SA is converted to MeSA by the activity of carboxyl methyltransferase (SAMT) enzymes (Dudareva et al. 1998). In Arabidopsis, this enzyme is BSMT1 (also named S-ADENOSYL-L-METHIONINE-DEPENDENT METHYLTRANSFERASE1 [SAMT1]), which uses SA as substrate and forms MeSA (Chen et al. 2003; Liu et al. 2010). MeSA was also proposed as a signal molecule in plant responses to abiotic stress, as it improved plant tolerance against chilling injury (Seydpour and Sayyari 2016; Ren et al. 2020; Gondor et al. 2022). However, the function and the regulatory mechanisms of *SAMT* expression during the cold stress response are still largely unknown.

BR signaling also regulates plant cold tolerance (Li et al. 2017; Fang et al. 2019). After BR perception at the plasma membrane by the receptors BR INSENSITIVE 1 (BRI1) and brassinosteroid-associated kinase 1 (BAK1, also named SOMATIC EMBRYOGENESIS RECEPTOR-LIKE KINASE 3 [SERK3]), a well-established cascade relays BR signals to members of the BRI1-EMS-SUPPRESSOR 1 (BES1) and BZR1 family of transcription factors, which control BR-regulated gene expression (Nolan et al. 2020). BRs promote plant cold tolerance via governing different cold-responsive transcriptional cascades through translational and post-translational modifications of key regulators (Eremina et al. 2016b). For example, active BR signaling leads to the accumulation of the active unphosphorylated form of BZR1, modulating plant freezing tolerance through CBF-dependent and -independent pathways that positively regulate cold stress responses (Li et al. 2017).

BRs integrate multiple phytohormone and environmental inputs, eventually leading to the activation or repression of BR-regulated genes. Extensive research in recent years has helped characterize the key points of interaction between BRs and other phytohormones or the signaling components regulating stress responses in plants (Keuskamp et al. 2011; Oh et al. 2012; Bernardo-García et al. 2014). However, whether or how the BR signaling pathway interacts synergistically with the SA signaling pathway to establish or modulate plant cold tolerance remains unknown.

Regulation of gene expression in response to cold stress often employs posttranslational histone modifications (To et al. 2011; Yuan et al. 2013; Kim et al. 2015; Ding et al. 2019). Furthermore, several studies have indicated that epigenetic modifications also have critical roles in coordinating environmental and BR signals (Yu et al. 2008; Sui et al. 2012; Zhang et al. 2014). However, a direct demonstration of a function for epigenetic regulators in establishing crosstalk with the BR and SA signaling pathways to confer cold tolerance is still elusive.

In this study, we report that the methyltransferase TaSAMT1 plays a pivotal role in freezing tolerance by promoting SA conversion to MeSA in wheat. Moreover, we demonstrate that TaBZR1, a master regulator of the BR signaling pathway, interacts with histone acetyltransferase of the GNAT family 1 (TaHAG1) to potentiate *TaSAMT1* transcription by increasing the levels of histone acetylation under cold stress, thus playing key roles in the freezing tolerance of wheat. These findings provide an in-depth understanding of cold stress tolerance in wheat and reveal a key regulatory module integrating the BR and SA signaling pathways, providing effective targets for breeding cold-tolerant wheat varieties.

## Results

### TaSAMT1 is a salicylate carboxyl methyltransferase in wheat

In a previous whole-transcriptome analysis of cold stress performed in wheat, the transcript levels of TraesCS3B02G445100 increased upon a cold treatment of 4°C compared to 22°C (Supplementary Fig. S1) and were induced by exposure to low temperatures (Li et al. 2015). Sequence analysis of the encoded protein revealed that this gene encodes a salicylate carboxyl methyltransferase, a putative ortholog of SAMTs from barley (*Hordeum vulgare*), purple false brome (*Brachypodium distachyon*), and rice (*Oryza sativa*); we thus named TraesCS3B02G445100 *TaSAMT1* (Supplementary Fig. S2). A BLAST search against the genomes of the wheat varieties Chinese Spring, Fielder, and others identified three *TaSAMT1* homoeologs in hexaploid wheat, designated as *TaSAMT1-A*, *TaSAMT1-B*, and *TaSAMT1-D*, according to their respective subgenomes (Walkowiak et al. 2020; Sato et al. 2021).

Intriguingly, the three TaSAMT1s encoded by these homoeologs showed substantial variation in sequence, with TaSAMT1-A consisting of a truncated peptide of only 130 amino acids (aa), TaSAMT1-B (encoded by TraesCS3B02G445100) being a 356-aa protein with a complete carboxyl methyltransferase domain, and TaSAMT1-D comprising 240 aa and lacking the N terminus present in the other two TaSAMT1s (Supplementary Fig. S3). In addition, a protein structure prediction analysis suggested that only TaSAMT1-B harbors a complete catalytic domain (Supplementary Fig. S3), prompting us to choose TaSAMT1-B for analysis of its role in cold responses. TaSAMT1-B clustered together with HvSAMT1, BdSAMT1, and OsSAMT1, with which it showed 90.45%, 63.1%, and 54.1% amino acid identity, respectively (Supplementary Fig. S2). We detected a complete SAM-dependent carboxyl methyltransferase domain in TaSAMT1-B and SAMT1s from the three other grasses, suggesting that TaSAMT1-B may play a conserved role in methylation (Supplementary Fig. S2).

**Figure 1.**
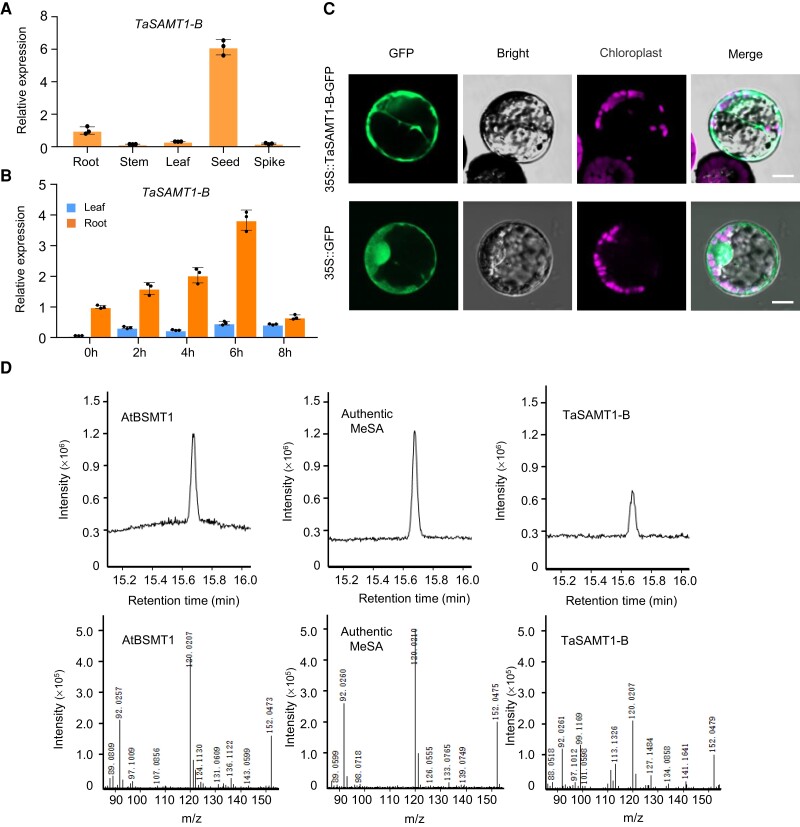
*TaSAMT1* expression pattern and enzyme activity of the encoded protein. **A)** Relative *TaSAMT1* transcript levels in the indicated tissues, as determined by RT-qPCR. **B)** Relative *TaSAMT1* transcript levels in response to treatment at −10°C for up to 8 h. Data were normalized to *β-ACTIN* and are shown as means ± standard deviation (SD, *n* = 3). **C)** Subcellular localization of TaSAMT1 in wheat leaf protoplasts. TaSAMT1-B (fused to GFP) localizes to the cytoplasm. Scale bars, 10 μm. **D)** Conversion of salicylic acid (SA) to methyl salicylate (MeSA) by TaSAMT1-B. Each enzymatic reaction was performed using SA as a substrate in the presence of S-adenosyl-L-methionine (SAM). Top, partial GC chromatograms of the MeSA standard and the reaction products from cell lysates containing AtBSMT1 or TaSAMT1, as analyzed by GC-MS; bottom, GC mass spectra of the authentic MeSA standard and the enzymatic products from cell lysates containing TaSAMT1.

*TaSAMT1* expression was highest in seeds, followed by roots, with low and similar levels in stems, leaves, and spikes (Fig. 1A). We also examined the response of *TaSAMT1* transcript levels to low-temperature stress by exposing seedlings to −10°C for up to 8 h. *TaSAMT1* expression was quickly induced in leaves and roots by freezing stress (Fig. 1B). To determine the subcellular localization of TaSAMT1, we transfected wheat protoplasts with a construct encoding a TaSAMT1-B-GFP fusion protein between TaSAMT1-B and green fluorescent protein (GFP). We detected green fluorescence signals mainly in the cytoplasm, suggesting that TaSAMT1-B is a cytosolic protein (Fig. 1C).

To test whether TaSAMT1 has enzymatic function, we produced and purified recombinant TaSAMT1-B fused to glutathione S-transferase (GST) in *Escherichia coli*, together with GST alone, TaSAMT1-A-GST, and TaSAMT1-D-GST, using AtBSMT1-GST as a positive control (Chen et al. 2003; Supplementary Fig. S4A). We then incubated crude bacterial protein extracts in the presence of SA to test methylation activity, revealing efficient catalysis of SA methylation with S-adenosyl-L-methionine (SAM) as a methyl donor for the cell lysates containing TaSAMT1-B or AtBSMT1 (Fig. 1D), but not those with TaSAMT1-A or TaSAMT1-D (Supplementary Fig. S4B), consistent with the absence of a complete carboxyl methyltransferase domain for these latter proteins (Supplementary Fig. S3). These results suggest that TaSAMT1-B converts SA to MeSA and contributes to the regulation of the cold response in wheat.

### Overexpression and knockout of *TaSAMT1-B* validate its role in freezing tolerance

**Figure 2.**
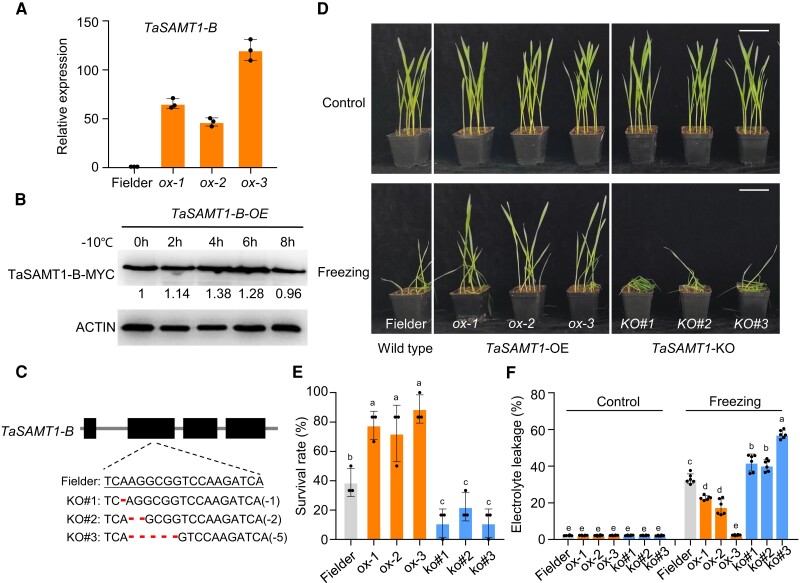
TaSAMT1 improves freezing tolerance in wheat. **A)** Relative *TaSAMT1-B* transcript levels in wild-type (Fielder) and *TaSAMT1-*OE (ox-1, ox-2, and ox-3) plants. Data are shown as means ± SD (*n* = 3). **B)** Immunoblot analysis of TaSAMT1-B-MYC in *TaSAMT1-*OE lines under freezing stress. Ten-day-old seedlings grown in normal conditions were exposed to −10°C for 2, 4, 6, or 8 h, or collected prior to cold treatment (0 h). Total proteins were extracted and subjected to immunoblot analysis with anti-MYC antibodies. **C)***TaSAMT1-B* sequences in the wild type (Fielder) and gene-edited mutants. The targeted sequence is underlined, and deleted nucleotides are indicated by red hyphens. The numbers to the right represent the number of deleted base pairs in each mutant. **D)** Ten-day-old wild-type Fielder, *TaSAMT1-*OE (ox-1, ox-2, and ox-3), and *Tasamt1-B* knockout (KO#1, KO#2, and KO#3) lines treated at −10°C for 1.5 h, followed by recovery for 24 h at 22°C. Representative photographs are shown. Scale bars, 5 cm. **E)** Survival rates of wild-type, *TaSAMT1-*OE, and *Tasamt1-B* lines after freezing treatment. Data are shown as means ± SD derived from measurements of at least 15 plants per genotype in three independent experiments. **F)** Electrolyte leakage of the wild-type Fielder, *TaSAMT1-*OE lines, and *Tasamt1-B* lines in control conditions and after freezing treatment. Data are shown as means ± SD (*n* = 6). The data in **(E)** and **(F)** were analyzed by one-way ANOVA followed by Tukey's test. Different lowercase letters indicate a significant difference (*P* < 0.05).

To investigate the role of TaSAMT1 in the cold tolerance of wheat, we generated transgenic wheat lines overexpressing *TaSAMT1-B-MYC* (*TaSAMT1*-*B-*OE). We obtained 11 putative *TaSAMT1-B* overexpression lines in the wheat cultivar Fielder and chose three independent *TaSAMT1*-*B-*OE lines (ox1, ox2, and ox3) with significantly elevated *TaSAMT1-B* transcript abundance for characterization (Fig. 2A). Immunoblot analysis with an anti-MYC antibody showed that TaSAMT1-B accumulates in the *TaSAMT1*-*B-*OE plants (Fig. 2B).

We also generated *Tasamt1* mutants in Fielder using clustered regularly interspaced short palindromic repeat (CRISPR)/CRISPR-associated nuclease 9 (Cas9)-mediated gene editing. We designed a single guide RNA (sgRNA) targeting a specific region in the second exon of *TaSAMT1-B*, since only TaSAMT1-B contains a complete carboxyl methyltransferase domain. We obtained three null mutants of *TaSAMT1-B* (KO#1, with a 1-bp deletion; KO#2, with a 2-bp deletion; and KO#3, with a 5-bp deletion in *TaSAMT1-B*) (Fig. 2C). To assess the performance of the *TaSAMT1*-*B-*OE and *Tasamt1* mutant lines under freezing stress conditions, we exposed 10-day-old seedlings (after germination) to −10°C for 1.5 h before returning them to normal conditions for recovery (Fig. 2D). When we scored the survival rates (SR) of all seedlings 24 h later, the *TaSAMT1*-*B-*OE plants had significantly higher SRs (83.3% in ox1, 75.0% in ox2, and 87.5% in ox3) than wild-type seedlings (∼33%) (Fig. 2, D and E). Conversely, when subjected to freezing stress, the SRs of the *Tasamt1* lines (8.3% in KO#1, 16.7% in KO#2, and 15.3% in KO#3) were significantly lower than that of wild-type plants (Fig. 2, D and E).

Cell membrane stability and integrity under temperature stress are important indicators of cold tolerance in plants. Therefore, we examined changes in the relative electrolyte leakage (REL) of the *TaSAMT1*-*B-*OE and *Tasamt1* lines. We observed no clear difference in REL between the wild-type, *TaSAMT1*-*B-*OE, and *Tasamt1* lines when grown at 22°C (Fig. 2F). However, after exposure to freezing treatment (−10°C for 1.5 h), the REL of all *TaSAMT1*-*B-*OE lines was significantly lower, while that of the *Tasamt1* lines was higher, compared to the wild type (Fig. 2F). Taken together, these results indicate that TaSAMT1-B is a positive regulator of cold tolerance in wheat.

### TaSAMT1-B affects MeSA and SA accumulation under freezing stress

**Figure 3.**
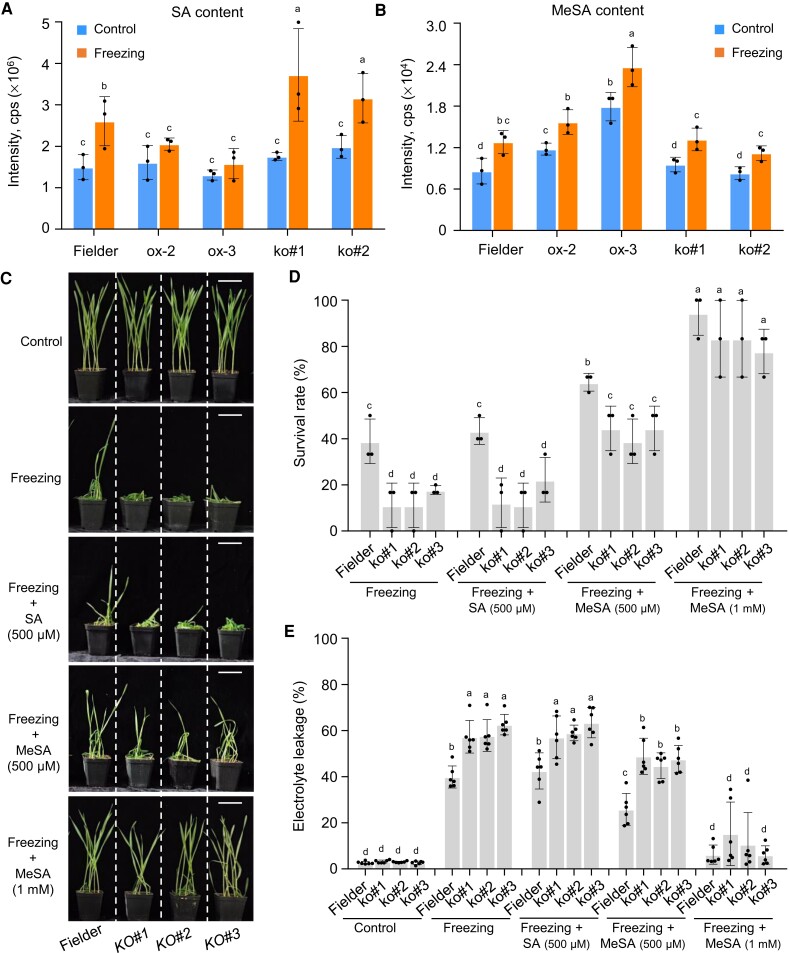
TaSAMT1 affects SA and MeSA accumulation under freezing stress. **A, B)** The content of endogenous SA **(A)** and MeSA **(B)** levels in wild-type Fielder, *TaSAMT1*-OE lines (ox-2 and ox-3), and *Tasamt1-B* lines (KO#1 and KO#2) under control and freezing treatment. **C)** Ten-day-old wild-type and *Tasamt1-B* (KO#1, KO#2, and KO#3) lines were sprayed with SA (500 μM) or MeSA (500 μM, 1 mM) before being exposed to −10°C for 1.5 h, followed by recovery for 24 h. Representative photographs are shown. Scale bars, 5 cm. **D)** Survival rates of the wild-type and *Tasamt1-B* lines after freezing treatment alone or together with spraying of 500 μM SA, 500 μM MeSA, or 1 mM MeSA. Data are shown as means ± SD derived from measurements of at least 15 plants per genotype in three independent experiments. **E)** Electrolyte leakage of the wild-type and *Tasamt1-B* lines in control and freezing conditions alone or together with spraying of 500 μM SA, 500 μM MeSA, or 1 mM MeSA. Data are shown as means ± SD (*n* = 6). The data in **(D)** and **(E)** were analyzed by one-way ANOVA followed by Tukey's test. Different lowercase letters indicate a significant difference (*P* < 0.05).

Multiple studies have proposed that MeSA may be an important messenger that plays a signaling role in regulating stress response and alleviating cold injury symptoms in crops (Park et al. 2007; Zhang et al. 2011; Siboza et al. 2014; Habibi et al. 2019). To investigate whether TaSAMT1-B affects endogenous MeSA levels to modulate tolerance under freezing stress, we measured the endogenous SA and MeSA levels in the wild type together with different *TaSAMT1*-*B-*OE and *Tasamt1* lines experiencing freezing stress. We established that MeSA accumulates to significantly higher levels in the *TaSAMT1*-*B-*OE lines relative to wild-type plants under both control and freezing conditions, while SA levels did not significantly increase upon exposure to freezing stress, in contrast to the wild type (Fig. 3, A and B). Consistent with this finding, MeSA levels were substantially lower and SA contents higher in *Tasamt1-B* lines exposed to freezing stress compared to wild-type plants (Fig. 3, A and B), demonstrating that TaSAMT1 is a major SA methyltransferase that converts SA to MeSA in response to freezing stress.

### Exogenous MeSA application rescues the compromised freezing tolerance of the *Tasamt1* mutant

To determine whether TaSAMT1 modulates freezing tolerance via MeSA accumulation, we assessed the effect of exogenous MeSA application on the freezing tolerance of *Tasamt1* mutants and wild-type plants (Fig. 3C). To this end, we measured SRs and cell membrane stability in the wild-type Fielder and *Tasamt1* lines exposed to freezing conditions alone or together with a prior spray of SA or MeSA at different concentrations (Fig. 3, D and E). When 500 μM MeSA was applied before freezing treatment, the SR and cell membrane stability of both wild-type and *Tasamt1* plants significantly improved over those seen following freezing stress treatment alone (Fig. 3, C to E). This response was dosage dependent, as an application of 1 mM MeSA fully rescued the SR and cell membrane stability defects seen in the *Tasamt1* mutants following freezing treatment, and comparable to wild-type Fielder under freezing stress (Fig. 3, C to E). This finding is consistent with a role for TaSAMT1 in freezing tolerance that is dependent on MeSA accumulation.

Notably, an exogenous application of 500 µM SA before freezing stress treatment resulted in no significant effect in SR or cell membrane stability of either wild-type or *Tasamt1* plants relative to plants experiencing freezing stress alone (Fig. 3, C to E). These results suggest that MeSA, but not SA, plays a vital role in freezing tolerance in wheat. Together, these results strongly indicate that TaSAMT1 is indeed involved in freezing tolerance through catalyzing SA methylation to produce MeSA.

### TaBZR1 directly binds to the promoter of *TaSAMT1*

**Figure 4.**
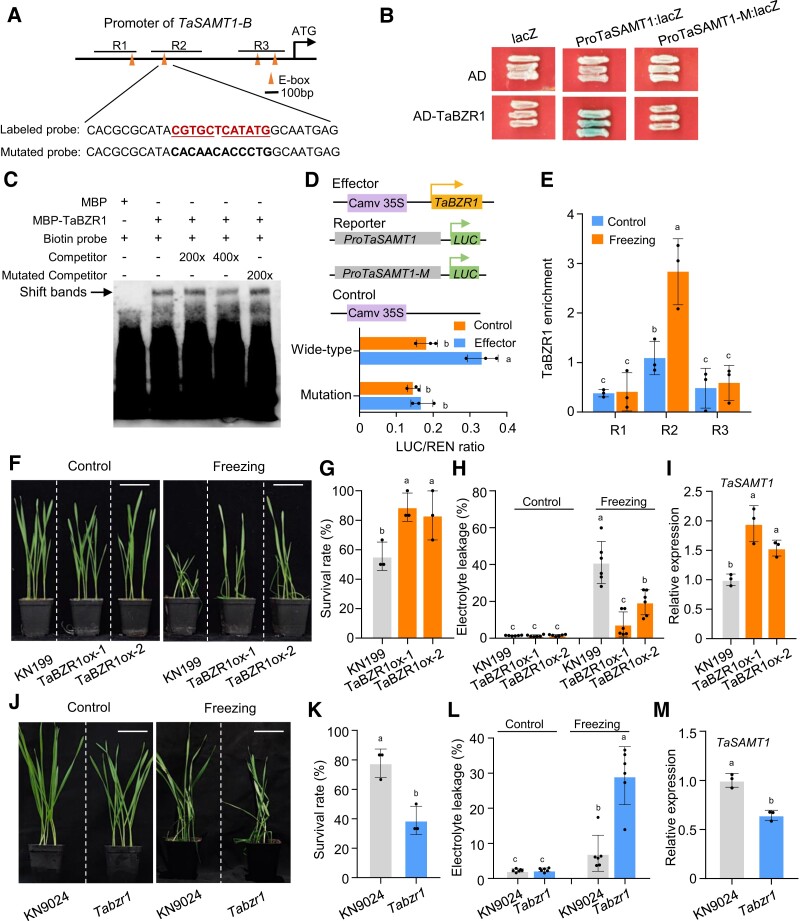
TaBZR1 directly regulates *TaSAMT1* transcription and contributes to wheat freezing tolerance. **A)** Diagram of the *TaSAMT1-B* promoter showing the positions of predicted TaBZR1-binding motifs and the sequences of the labeled probe (red font indicates E-box) and mutated probes used for electrophoretic mobility shift assay (EMSA). **B)** Yeast one-hybrid assays showing the binding of TaBZR1 to the *TaSAMT1-B* promoter. pLACZi2*μ* co-transformed with pB42AD was used as negative control. **C)** EMSA showing the binding of TaBZR1 to the *TaSAMT1-B* promoter. Biotin-labeled fragments representing parts of the *TaSAMT1-B* promoter containing the E-box were incubated with recombinant MBP or MBP-TaBZR1. Competitor or mutated competitor probes were added in 200× or 400× excess to analyze the specificity of binding. **D)** Transactivation of the *TaSAMT1* promoter by TaBZR1 in wheat protoplasts. The two reporters represent wild-type (*proTaSAMT1*) and mutated (*proTaSAMT1*-M) 1-kb *TaSAMT1* promoter fragments. LUC, firefly luciferase; REN, *Renilla* luciferase. Data are shown as means ± SD (*n* = 3). **E)** Chromatin immunoprecipitation followed by quantitative PCR (ChIP-qPCR) validation of TaBZR1-binding sites on the *TaSAMT1-B* promoter. Enrichment of the R1, R2, and R3 fragments of the *TaSAMT1-B* promoter was determined in chromatin precipitated with anti-BZR1 antibodies. **F)** Ten-day-old seedlings from the wild-type KN199 and *TaBZR1*-OE lines (ox-1 and ox-2) were exposed to −10°C for 1.5 h, then allowed to recover for 24 h. Representative photographs are shown. Scale bars, 5 cm. **G)** Survival rates of KN199 and *TaBZR1*-OE lines after freezing treatment. Data are shown as means ± SD derived from measurements of at least 15 plants for each genotype in three independent experiments. **H)** Electrolyte leakage of KN199 and *TaBZR1*-OE lines in control and freezing conditions. Data are shown as means ± SD (*n* = 6). **I)** Relative *TaSAMT1-B* transcript levels in KN199 and *TaBZR1-*OE plants. Data were normalized to wheat *TaACTIN* and shown as means ± SD (*n* = 3). **J)** Ten-day-old wild-type KN9204 and *Tabzr1* mutant seedlings were exposed to −10°C for 1.5 h, then allowed to recover for 24 h. Representative photographs are shown. Scale bars, 5 cm. **K)** Survival rates of KN9204 and *Tabzr1* after freezing treatment. Data are shown as means ± SD derived from measurements of at least 15 plants per genotype in three independent experiments. **L)** Electrolyte leakage of KN9204 and *Tabzr1* in control and freezing conditions. Data are shown as means ± SD (*n* = 6). **M)** Relative *TaSAMT1-B* transcript levels in KN9204 and *Tabzr1* plants. Data were normalized to *TaACTIN* and are shown as means ± SD (*n* = 3). The data in **(G, H, I)** and **(K, L, M)** were analyzed by one-way ANOVA followed by Tukey's test. Different lowercase letters indicate a significant difference (*P* < 0.05).

To investigate the molecular mechanism by which cold stress triggers *TaSAMT1* expression, we performed a yeast one-hybrid screen using the *TaSAMT1-B* promoter as a bait and cDNA prepared from total wheat RNA extracted from leaves and roots as a library. We thus identified 91 potential upstream regulators of *TaSAMT1-B* transcription, based on their ability to bind to the *TaSAMT1-B* promoter (Supplementary Data Set 1). Among them, we noticed a putative BZR1 family member encoded by TraesCS2A02G187800, which we confirmed as binding to the *TaSAMT1-B* promoter in directed yeast one-hybrid assays (Fig. 4, A and B). Sequence alignment indicated that the protein encoded by TraesCS2A02G187800 contains a basic helix–loop–helix (bHLH)-like DNA-binding domain and shares the highest sequence identity with Os07g0580500 and AtBZR1; we thus named this protein TaBZR1 (Supplementary Fig. S5, A and B).

In addition, RT-qPCR and immunoblot analyses indicated that *TaBZR1* transcript levels and TaBZR1 protein abundance are significantly enhanced in wheat under freezing stress, with an expression pattern for *TaBZR1* similar to that of *TaSAMT1* (Supplementary Fig. S6, A and B). As a master transcriptional regulator in the BR signaling pathway, BZR1 binds to BR response elements (BRREs, CGTG(T/C)) and E-box (CANNTG) motifs in the promoters of its target genes (Sun et al. 2010; Nolan et al. 2020). We thus analyzed the sequences of the *TaSAMT1-B* promoter (defined as up to 1 kb upstream from the ATG) and identified several putative E-box motifs (Fig. 4A). We found the TaBZR1 could directly bind to the promoter of *TaSAMT1* region R2 containing E-box motif from −679 bp to −387 bp (Fig. 4B).

To further test the binding of TaBZR1 to the R2 region of the *TaSAMT1-B* promoter, we performed an electrophoretic mobility shift assay (EMSA) using recombinant purified TaBZR1 fused to maltose-binding protein (MBP) and a labeled probe with an E-box motif from the *TaSAMT1* promoter (−614 to −584 bp, in the center of the R2 region above). We determined that recombinant MBP-TaBZR1 binds to the labeled probe but not to a mutated version of the probe that abolishes the E-box motif (Fig. 4C). Y1H assays also showed no binding between a mutated version of the *TaSAMT1-B* promoter lacking the E-box motif with the R2 region and TaBZR1 (Fig. 4B). We also performed firefly luciferase (LUC) activity assays to examine the transactivation capacity of TaBZR1 toward the *TaSAMT1-B* promoter. To this end, we placed the *LUC* reporter gene under the control of the *TaSAMT1-B* promoter (1 kb upstream from the ATG, as above) and transfected the resulting *proTaSAMT1-B:LUC* construct into wheat protoplasts. LUC activity derived from the *proTaSAMT1* construct increased significantly when co-transfected with an effector construct expressing *TaBZR1*, compared to the empty vector control (Fig. 4D). However, disruption of the E-box abolished TaBZR1-mediated transactivation of the *TaSAMT1* promoter (Fig. 4D), indicating that TaBZR1 positively regulates *TaSAMT1* transcription. Moreover, chromatin immunoprecipitation followed by qPCR (ChIP-qPCR) with an anti-BZR1 antibody confirmed that TaBZR1 binds directly to the E-box motif-containing segments within the *TaSAMT1-B* promoter regions. Specifically, freezing stress treatment enhanced the enrichment of the R2 region containing the E-box motif from the *TaSAMT1-B* promoter in the precipitated chromatin, in line with *TaSAMT1-B* elevated transcriptional activation compared to mock treatment (Fig. 4E). Together, these results indicate that TaSAMT1-B may be an important TaBZR1 target for freezing tolerance, thus connecting the BR and SA pathways.

### TaBZR1 contributes to freezing tolerance in wheat

To investigate the possible role of TaBZR1 in freezing tolerance in wheat, we obtained *TaBZR1* overexpression lines in wheat (*TaBZR1-*OE) (Yang et al. 2023). RT-qPCR analysis showed significantly higher *TaBZR1* transcript levels in the *TaBZR1-*OE lines compared with wild-type plants (Supplementary Fig. S6C). Moreover, we identified a *Tabzr1* mutant by screening a mutant library in the KN9204 (a wheat variety) background. The mutant carries a G-to-A transition in the second exon of TraesCS2A02G187800, resulting in the introduction of a premature translation termination codon (Supplementary Fig. S7A). Immunoblot analysis with an anti-BZR1 antibody revealed a 50% decrease in TaBZR1 abundance in the *Tabzr1* mutant compared to the wild type (Supplementary Fig. S7B).

As with the *TaSAMT1-B-*OE and *Tasamt1-B* lines above, we assessed the performance of the *TaBZR1*-OE and *Tabzr1* mutant lines under freezing stress conditions (Fig. 4, F and J). When subjected to freezing treatment, the SR was significantly higher in *TaBZR1-*OE lines and lower in the *Tabzr1* mutant relative to the wild type (Fig. 4, G and K). While there was no clear difference in REL among the wild-type, *TaBZR1-*OE, and *Tabzr1* plants when grown at 22°C (Fig. 4, H and L), the REL after freezing treatment decreased in the *TaBZR1-*OE lines but increased in the *Tabzr1* mutant, compared to the wild type (Fig. 4, H and L). These results indicate that TaBZR1 participates in wheat freezing tolerance.

We assessed the relative *TaSAMT1-B* expression levels in the *TaBZR1*-OE lines and *Tabzr1* mutant plants. *TaSAMT1-B* expression was upregulated in the *TaBZR1*-OE lines but downregulated in the *Tabzr1* mutant compared to the wild type (Fig. 4, I and M). Accordingly, MeSA accumulated to significantly higher levels in *TaBZR1*-OE lines compared to wild-type plants exposed to freezing stress (Supplementary Fig. S8). These results are consistent with our hypothesis that TaBZR1 functions as a positive regulator of *TaSAMT1-B* expression and MeSA accumulation, leading to elevated freezing tolerance.

### *TaSAMT1-B* is required for the role of the TaBZR1-mediated BR pathway in freezing tolerance

**Figure 5.**
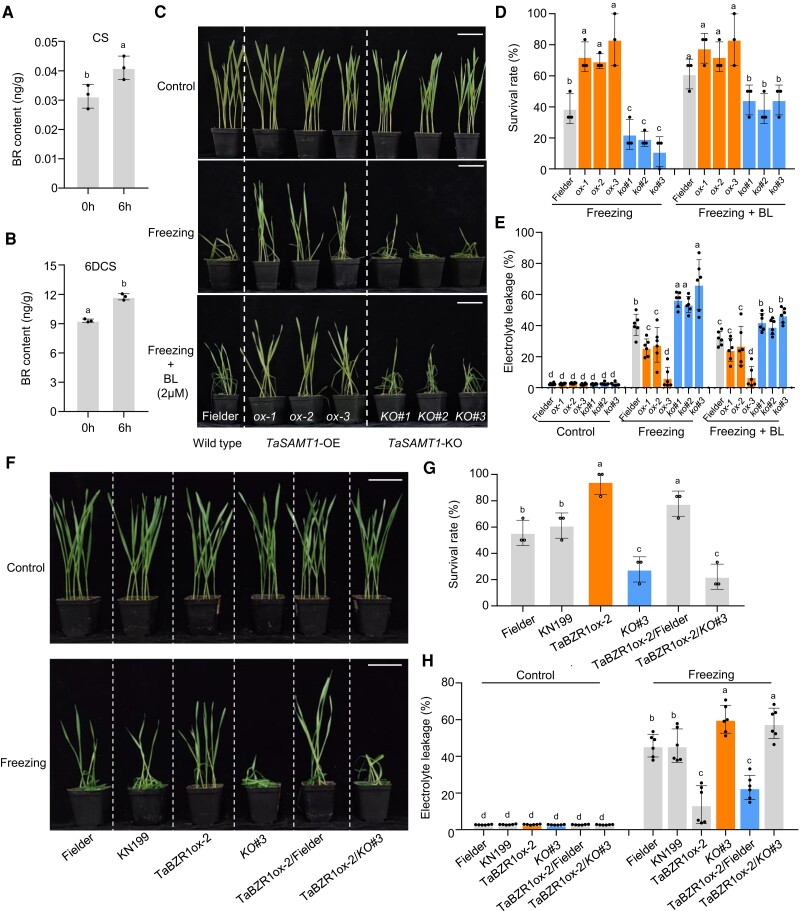
TaSAMT1-B is required for the TaBZR1-mediated involvement of the BR pathway in freezing tolerance. **A**, **B)** Levels of castasterone (CS) and 6-deoxocastasterone (6DCS) in wild-type Fielder under control and freezing conditions. Data are shown as means ± SD (*n* = 3 biologically independent samples). **C)** Ten-day-old wild-type Fielder, *TaSAMT1*-OE (ox-1, ox-2, and ox-3), and *Tasamt1-B* (KO#1, KO#2, and KO#3) lines sprayed with 2 μM brassinolide (BL), exposed to −10°C for 1.5 h, then allowed to recover for 24 h. Scale bars, 5 cm. **D)** Survival rates of wild-type Fielder, *TaSAMT1*-OE, and *Tasamt1-B* lines following freezing treatment alone or together with prior spraying with 2 μM BL. Data are shown as means ± SD derived from measurements of at least 15 plants per genotype in three independent experiments. **E)** Electrolyte leakage of the wild-type, *TaSAMT1*-OE, and *Tasamt1-B* lines after freezing treatment and/or spraying with 2 μM BL. Data are shown as means ± SD (*n* = 6). **F)** Phenotypes of Fielder, KN199, *TaBZR1* ox-2, one *Tasamt1-B* line (KO#3), *TaBZR1* ox-2 in Fielder, and *TaBZR1*ox-2 in *Tasamt1-B* KO#3 under freezing treatment (−10°C for 1.5 h, followed by recovery for 24 h). Representative photographs are shown. Scale bars, 5 cm. **G)** Survival rates of the indicated genotypes after freezing treatment. Data are shown as means ± SD derived from measurements of at least 15 plants per genotype in three independent experiments. **H)** Electrolyte leakage of the indicated genotypes in control and freezing conditions. Data are shown as means ± SD (*n* = 6). The data were analyzed by one-way ANOVA followed by an LSD test. Different lowercase letters indicate a significant difference (*P* < 0.05).

To check whether the BR pathway is involved in the cold response of wheat, we measured the endogenous levels of BRs in wheat seedlings under freezing stress. We detected higher levels of endogenous BRs, including castasterone and 6-deoxocastasterone, in wild-type seedlings under cold stress (Fig. 5, A and B). Moreover, relative *TaBZR1* transcript levels were upregulated after BL treatment (Supplementary Fig. S9A), as were those of *TaSAMT1* (Supplementary Fig. S9B). To further examine whether the BR signaling pathway regulates cold tolerance synergistically with the SA pathway via TaSAMT1 in wheat, we exposed wild-type, *TaSAMT1*-*B-*OE, and *Tasamt1-B* plants to freezing stress alone or following a prior spray with brassinolide (BL) (Fig. 5C).

The effects of BL application on survival rate and cell membrane stability under cold stress were negligible in *TaSAMT1-B*-OE lines between freezing stress alone and combined freezing stress and BL treatment, consistent with the strong cold tolerance of these lines (Fig. 5, C to E). By contrast, an application of BL onto wild-type and *Tasamt1-B* seedlings before freezing stress treatment improved their survival rate and cell membrane stability compared to freezing stress treatment alone. Notably, the application of BL only partially alleviated the freezing sensitivity of *Tasamt1-B* seedlings and was not sufficient to return to wild-type levels (Fig. 5, C to E), suggesting the TaSAMT1-B functions downstream of the BR pathway and is important for BR-mediated freezing tolerance.

To test this hypothesis genetically, we crossed a *TaBZR1*-OE line (ox-2) to a *Tasamt1-B* mutant line (KO#3) and identified plants homozygous for the mutation of *TaSAMT1-B* and overexpressing *TaBZR1*. This *Tasamt1-B TaBZR1*-OE line shared the same decreased SR and cell membrane integrity phenotypes as the *Tasamt1-B* mutant under freezing treatment (Fig. 5, F to H), indicating that *TaSAMT1-B* functions downstream of *TaBZR1* and modulates freezing tolerance. Collectively, these results suggest that TaSAMT1-B is required for the BR-regulated pathway and plays a vital role in freezing tolerance in wheat.

### TaBZR1 interacts with the histone acetyltransferase TaHAG1

To explore the regulatory mechanisms of TaBZR1 in the transcriptional regulation of *TaSAMT1-B*, we performed a yeast two-hybrid screen using TaBZR1 as bait against a wheat cDNA library prepared from total RNA extracted from seedling leaves and roots of wheat. We identified 192 potential TaBZR1-interacting proteins (Supplementary Data Set 2). Among them, a putative plant histone acetyltransferase protein TaHAG1 (also named GENERAL CONTROL NONDEREPRESSIBLE 5 [GCN5]) encoded by TraesCS1D02G134200 caught our attention; we confirmed its interaction with TaBZR1 in a targeted yeast two-hybrid assay (Supplementary Fig. S10A).

**Figure 6.**
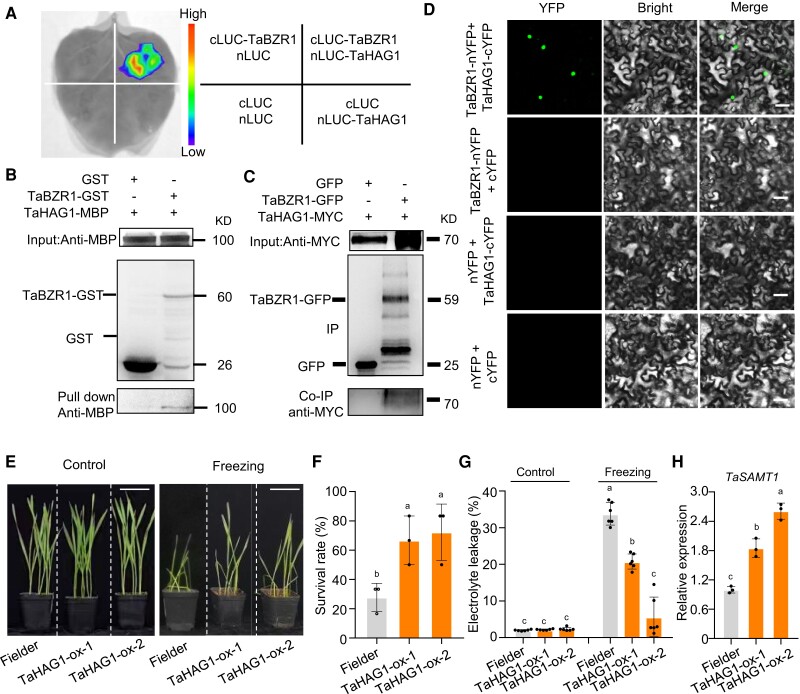
TaBZR1 physically interacts with histone acetyltransferase TaHAG1. **A)** Luciferase complementation imaging (LCI) assays. The construct encoding nLUC-tagged TaHAG1 was co-infiltrated into the leaves of *N. benthamiana* plants along with a construct encoding cLUC-targeted TaBZR1. **B)** MBP pull-down assay showing the interaction of recombinant TaHAG1-MBP with TaBZR1-GST. TaBZR1-GST was detected using anti-GST antibody. TaBZR1-GST was incubated with immobilized MBP or TaHAG1-MBP. Immunoprecipitated fractions were probed by immunoblotting with an anti-MBP and anti-GST antibody. Protein size (kD) is provided to the right of the gel. **C)** Co-immunoprecipitation (Co-IP) showing the physical interaction of TaBZR1 and TaHAG1. Protein extracts from the leaves of *N. benthamiana* plants (Input) and after IP with GFP-trap beads were probed by immunoblotting using an anti-MYC antibody. **D)** Bimolecular fluorescence complementation (BiFC) assay showing that TaBZR1 interacts with TaHAG1 in nuclei. Constructs encoding TaBZR1 and TaHAG1 fused to the N terminus or the C terminus of yellow fluorescent protein (YFP) were co-infiltrated into the leaves of *N. benthamiana* plants. Scale bars, 50 μm. **E)** Ten-day-old wild-type Fielder and *TaHAG1*-OE (ox-1 and ox-2) lines were exposed to −10°C for 1.5 h, followed by recovery for 24 h. Scale bars, 5 cm. **F)** Survival rates of the wild-type and *TaHAG1*-OE lines after freezing treatment. Data are shown as means ± SD derived from measurements of at least 15 plants per genotype in three independent experiments. **G)** Electrolyte leakage of the wild-type and *TaHAG1*-OE lines in control and freezing conditions. Data are shown as means ± SD (*n* = 6). **H)** Relative *TaSAMT1* transcript levels in wild-type Fielder and *TaHAG1*-OE plants. Data were normalized to *TaACTIN* and are shown as means ± SD (*n* = 3). The data in **(F)**, **(G)**, and **(H)** were analyzed by one-way ANOVA followed by an LSD test. Different lowercase letters indicate a significant difference (*P* < 0.05).

To corroborate the interaction between TaBZR1 and TaHAG1 in planta, we performed luciferase complementation imaging (LCI) assays, which showed that co-expression of cLUC-TaBZR1 and nLUC-TaHAG1 in the leaves of *Nicotiana benthamiana* plants yields strong luciferase activity; however, we detected no signal in leaves expressing the empty control constructs or when co-expressing cLUC-TaBZR1 with nLUC-TaSRT1 (SIRTUIN 1) or co-expressing nLUC-TaHAG1 with cLUC-TaDT-A (Drought Tolerant-A), although cLUC-TaDT-A interacts with nLUC-TaSRT1 (Fig. 6A and Supplementary Fig. S10B). GST pull-down assays also showed that TaBZR1-GST directly interacts with TaHAG1-MBP (Fig. 6B). Furthermore, we conducted co-immunoprecipitation (Co-IP) assays of protein extracts from *N. benthamiana* leaves expressing *TaBZR1-GFP*, *TaHAG1-MYC*, and the *GFP* alone via Agrobacterium (*Agrobacterium tumefaciens*)-mediated infiltration. Following an IP with an anti-GFP antibody, an immunoblot analysis with an anti-MYC antibody detected a band corresponding to TaHAG1-MYC in the samples co-expressing *TaBZR1-GFP* and *TaHAG1-MYC*, but not in those expressing *TaHAG1-MYC* and *GFP* (Fig. 6C). We then confirmed the TaBZR1–TaHAG1 interaction using bimolecular fluorescence complementation (BiFC) assays in which an excited YFP reconstitution signal was observed using a laser scanning confocal fluorescence microscope when the fusion constructs TaBZR1-nYFP and TaHAG1-cYFP were present, but not when those transformed with the corresponding control were present (Fig. 6D). Meanwhile, no interaction was detected between TaBZR1-nYFP and TaSRT1-cYFP or TaHAG1-cYFP and TaDT-A-nYFP, although TaSRT1 interacts with TaDT-A in nuclei (Supplementary Fig. S10C), suggesting that the TaBZR1–TaHAG1 interaction in vivo is specific. Collectively, these results demonstrate the TaBZR1–TaHAG1 interaction in vitro and in vivo, suggesting that TaHAG1 might participate in the TaBZR1 pathway under freezing stress in wheat.

### TaHAG1 positively regulates freezing stress tolerance in wheat

To determine whether *TaHAG1* responds to cold tolerance, we checked the expression pattern of *TaHAG1* under freezing stress. RT-qPCR analysis showed that *TaHAG1* transcript levels are upregulated by up to 50% under freezing stress relative to control conditions (Supplementary Fig. S11A). To characterize TaHAG1 function in the wheat response to freezing stress, we obtained *TaHAG1* overexpression lines with significantly higher transcript levels compared to Fielder (Supplementary Fig. S11B; Zheng et al. 2021). Under freezing treatment, the SR of *TaHAG1-*OE lines was significantly higher than that of the wild type (Fig. 6, E and F). In addition, while the REL was comparable between *TaHAG1-*OE lines and wild-type seedlings grown under normal conditions, the REL values of the *TaHAG1-*OE lines were lower than that of the wild type following exposure to freezing conditions (Fig. 6G). These results indicate that TaHAG1 participates in the freezing tolerance of wheat.

### TaHAG1 physically associates with the *TaSAMT1* promoter and facilitates its transcription by modulating histone acetylation

The above findings that TaBZR1 targets and facilitates *TaSAMT1-B* transcription, together with the physical interaction of TaBZR1 and TaHAG1, prompted us to investigate whether *TaSAMT1-B* is a direct target of TaHAG1. To this end, we examined *TaSAMT1-B* transcripts levels in *TaHAG1*-OE and wild-type seedlings by RT-qPCR. *TaSAMT1-B* expression was upregulated in *TaHAG1*-OE lines compared to the wild type (Fig. 6H). Consistent with this observation, endogenous MeSA levels were significantly higher in *TaHAG1*-OE lines than in wild-type seedlings exposed to freezing stress conditions (Supplementary Fig. S12).

**Figure 7.**
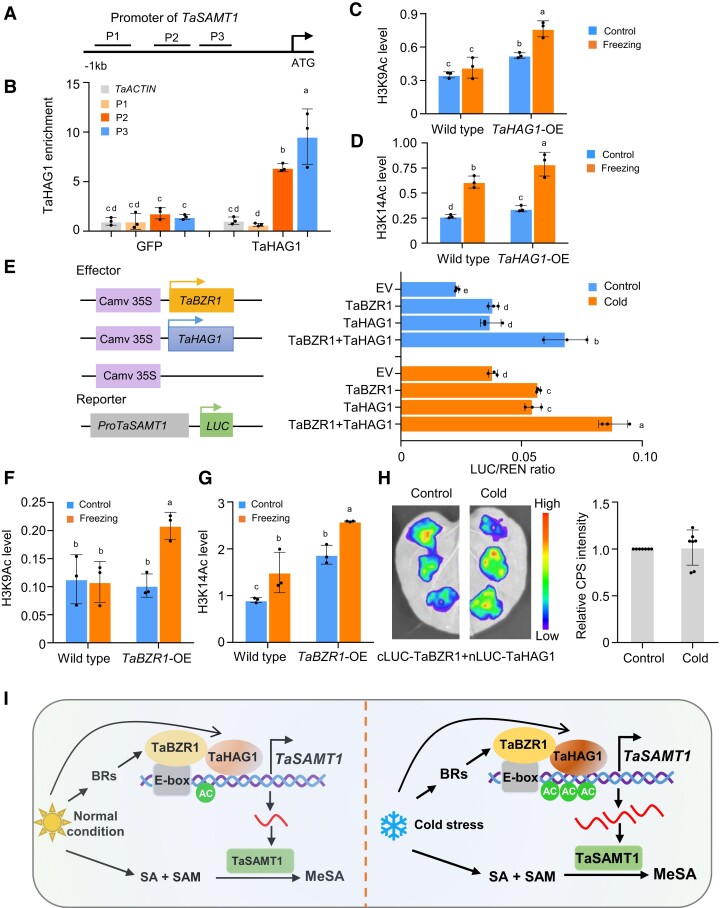
TaBZR1 and TaHAG1 cooperate to activate *TaSAMT1-B* transcription. **A**, **B)** ChIP-qPCR validation of TaHAG1-binding sites in the *TaSAMT1-B* promoter. Enrichment of the P1, P2, and P3 fragments of the *TaSAMT1-B* promoter was determined in chromatin precipitated with anti-GFP antibodies in *TaHAG1*-GFP lines. **C, D)** Levels of H3K9ac **(C)** and H3K14ac **(D)** modification along the *TaSAMT1-B* promoter region under control and freezing conditions. Data are shown as means ± SD (*n* = 3). **E)** Dual luciferase reporter assays in wheat protoplasts showing the ability of TaHAG1 and TaBZR1 to transactivate *TaSAMT1-B* promoter activity before and after freezing treatment (4°C in dark for 2 h). Left, diagrams of the effector and reporter constructs. Right, ratio of firefly luciferase (LUC) to *Renilla* luciferase (REN) indicating the transactivation ability of TaBZR1 and/or TaHAG1. Data are shown as means ± SD (*n* = 3). **F, G)** Enrichment of H3K9ac **(F)** and H3K14ac **(G)** in the *TaSAMT1-B* promoter region in wild-type KN199 and *TaBZR1*-OE lines under control and freezing conditions. Data are shown as means ± SD (*n* = 3). **H)** LCI assays. The construct encoding nLUC-tagged TaBZR1 was co-infiltrated into *N. benthamiana* leaves with a construct encoding cLUC-tagged TaHAG1 before and after cold treatment. Data are shown as means ± SD (*n* = 7). The relative luminescence values in control were set to 1. The data were analyzed by one-way ANOVA followed by an LSD test. Different lowercase letters indicate a significant difference (*P* < 0.05). **I)** A proposed working model for the TaHAG1–TaBZR1 cooperation in activating *TaSAMT1* transcription for freezing tolerance. TaBZR1 alone establishes a basic transregulation system for activating the *TaSAMT1* expression through its binding to the E-box; the interaction of TaHAG1 with TaBZR1 is necessary for enhanced transregulation via increasing histone acetylation. Higher TaSAMT1 levels in turn result in accumulation of MeSA and enhanced freezing tolerance.

To determine whether TaHAG1 binds directly to the *TaSAMT1-B* promoter in vivo, we performed ChIP-qPCR using *proUbi*:*TaHAG1*-*GFP* seedlings. We detected a significant enrichment of the P2 and P3 fragments (−760 to −360 bp) of the *TaSAMT1-B* promoter in the chromatin precipitated with anti-GFP antibodies (Fig. 7, A and B). Since TaHAG1 is mainly involved in histone H3 lysine 9 and 14 acetylation in wheat (Zheng et al. 2021), we investigated the levels of H3K9 and H3K14 acetylation at the P3 fragment of *TaSAMT1-B* promoter. Accordingly, we performed ChIP-qPCR assays with antibodies against H3K9ac and H3K14ac using wild-type and *TaHAG1*-OE seedlings grown under control or freezing stress conditions. We observed low levels of the H3K9ac and/or H3K14ac modification over the *TaSAMT1-B* promoter in control wild-type seedlings, with significantly higher H3K14ac levels seen after freezing stress. Notably, H3K9ac and H3K14ac levels along the *TaSAMT1-B* promoter were significantly increased in *TaHAG1*-OE seedlings compared to wild-type seedlings under control and freezing stress conditions (Fig. 7, C and D).

We also used a transient expression system in wheat protoplasts to assess whether TaHAG1 induces *TaSAMT1* transcription in vivo. The co-expression of *TaHAG1* with a *proTaSAMT1-B:LUC* reporter construct resulted in significantly increased LUC activity compared to control samples with the empty vector control (Fig. 7E), indicating that TaHAG1 activates the transcription of *TaSAMT1-B*. Together, based on these findings, we propose that TaHAG1 directly binds to the *TaSAMT1-B* promoter to trigger epigenetic changes, which in turn facilitate its transcription in response to freezing stress.

### TaBZR1 and TaHAG1 cooperatively activate *TaSAMT1* transcription

The in vitro and in vivo protein interaction assays suggested that TaBZR1 and TaHAG1 may form a protein complex, which may bind to the *TaSAMT1-B* promoter and induce transcription. We therefore tested whether co-expression of *TaHAG1* affected the TaBZR1-mediated transcriptional regulation of *TaSAMT1-B* in the same transient expression system used above with the *proTaSAMT1-B:LUC* reporter (Fig. 7E). Indeed, while the single expression of *TaBZR1* or *TaHAG1* activated transcription from the *TaSAMT1-B* promoter, their co-expression led to a significant increase in *TaSAMT1-B* promoter activation under control and freezing stress conditions. Moreover, the levels of H3K9ac at the *TaSAMT1-B* promoter were significantly increased in *TaBZR1*-OE seedlings compared to the wild type under freezing treatment; H3K14ac levels were significantly higher in *TaBZR1*-OE seedlings than in the wild type under both control and freezing stress conditions (Fig. 7, F and G). Thus, the overexpression of *TaBZR1* triggers increased H3K9ac, H3K14ac modification, and transcriptional upregulation of the *TaSAMT1-B* locus under cold stress.

To connect the changes in histone acetylation and transcriptional activation of the *TaSAMT1-B* locus to the BR pathway, we performed a ChIP-qPCR assay for TaBZR1 on wild-type seedlings grown under control conditions or treated with BL. We observed an enrichment for TaBZR1 at the *TaSAMT1-B* promoter upon BL treatment (Supplementary Fig. S13A). In addition, we detected increased H3K14ac levels over the *TaSAMT1* promoter following BL treatment relative to control seedlings; however, H3K9ac levels remained unchanged (Supplementary Fig. S13, B and C). Notably, these changes in acetylation levels were not caused by changes in the affinity between TaBZR1 and TaHAG1, as determined by LCI assays in *N. benthamiana* leaves co-expressing *cLUC-TaBZR1* and *nLUC-TaHAG1* exposed to control or freezing stress conditions (Fig. 7H). Taken together, these results indicate that TaBZR1 directly binds to the *TaSAMT1-B* promoter and further activates its transcription under cold stress by recruiting TaHAG1 and increasing the levels of histone H3 acetylation.

## Discussion

Identifying the function of key modulators and exploring the regulatory mechanisms in plant cold stress responses are crucial to developing a strategy to improve cold tolerance and ensure crop productivity under adverse climate conditions. Genetic and molecular analyses have revealed major quantitative trait loci (QTLs) and genes that affect cold tolerance in crops (Fujino et al. 2008; Saito et al. 2010; Ma et al. 2015; Zhang et al. 2017; Liu et al. 2018; Mao et al. 2019; Zeng et al. 2021). However, the identity and nature of the underlying mechanisms remain to be fully dissected. This question is particularly challenging in common wheat, which carries a large hexaploid genome with limited gene editing efficiency. Here, we demonstrate that the SA methyl transferase TaSAMT1-B, which methylates SA into MeSA in vitro, plays a vital role in the freezing tolerance of wheat. We further reveal a key transcriptional regulatory node consisting of TaBZR1, the master regulator of BR signaling, interacting with the epigenetic modulator TaHAG1 to confer freezing tolerance to wheat through potentiating the transcriptional output of *TaSAMT1-B*.

SA and BR are two well-characterized signaling molecules in terms of their biosynthesis and signal transduction (Kim et al. 2022). SA plays key roles in plant immune responses, including resistance to pathogen attack, the hypersensitive response, cell death, and SAR (Ding and Ding 2020). Accumulating evidence indicates that SA is also involved in responses to abiotic stresses, including drought, salinity, and low temperature (Miura and Tada 2014). However, a mechanistic understanding of the potential interaction between the SA pathway and the cold stress response is extremely limited, although a recent study reported that the SA receptor NON-EXPRESSER OF PATHOGENESIS-RELATED GENES 1 (NPR1) plays an important role in freezing tolerance in Arabidopsis independently of the SA pathway (Olate et al. 2018).

In the present study, knocking out *TaSAMT1-B* resulted in lower MeSA accumulation and decreased tolerance to freezing stress, whereas overexpression of *TaSAMT1-B* led to elevated MeSA levels and enhanced tolerance to freezing stress. The higher content of MeSA and lower content of SA in *TaSAMT1*-*B-*OE lines indicate that *TaSAMT1-B* converts SA to MeSA in response to freezing stress (Fig. 3, A and B). We confirmed the positive effect of MeSA on cold tolerance when spraying seedlings with MeSA prior to imposing freezing conditions (Fig. 3, C to E). Therefore, our results demonstrate that the SA methyl transferase TaSAMT1-B is involved in MeSA accumulation upon freezing stress and is essential for cold tolerance in wheat.

The biological role of MeSA, a methyl ester form of SA, is not as well known as that of SA. SA is methylated to MeSA by SAMT, while MeSA is converted to SA by SA binding protein 2 (SABP2) (Kumar 2014). Since SA and MeSA are reciprocal precursors, it is often difficult to differentiate between their individual effects (Gondor et al. 2022). Several studies have shown that application of MeSA improves the cold stress tolerance of plants (Sayyari et al. 2011; Seydpour and Sayyari 2016; Fan et al. 2021; Zeng et al. 2022). Moreover, a recent study revealed that the volatile MeSA is emitted by cold-stressed tea (*Camellia sinensis*) plants and plays a pivotal role in priming cold tolerance for plant-to-plant communication via a CBF-dependent pathway (Zhao et al. 2020). MeSA is more hydrophobic and volatile than SA, and it can spread more easily and act faster than SA (Gondor et al. 2022). Together with our findings, we propose that MeSA might be a mobile signal in response to cold stress in plants. There is evidence that MeSA mediates cold signaling via the CBF-dependent pathway (Zhao et al. 2020); however, the mechanism by which MeSA promotes *CBF* expression and how MeSA modulates SA-mediated cold stress responses remain to be elucidated.

To mount protection against cold stress, endogenous BRs promote cold tolerance through the accumulation of the active unphosphorylated forms of BZR1 and BES1, leading to the activation of CBF-dependent or -independent signaling cascades (Li et al. 2017; Nolan et al. 2020). The relationships between BRs and other phytohormones have been widely studied and well established (De Vleesschauwer et al. 2012; Nolan et al. 2020). By contrast, less is known about whether BRs are engaged in physiological and molecular interactions with the SA signaling cascades to modulate plant growth and responses to stress. One study reported that BRs enhanced SA-mediated immune responses by inhibiting BRASSINOSTEROID-INSENSITIVE 2 (BIN2)-mediated phosphorylation of clade I TGA transcription factors in Arabidopsis (Kim et al. 2022).

In this study, a series of genetic and biochemical analyses revealed that the TaBZR1–TaSAMT1-B module plays an essential role in mediating the crosstalk between SA and BR signaling in response to cold stress. TaBZR1 directly targets the *TaSAMT1-B* promoter to play a key role in freezing tolerance in wheat (Fig. 4). Therefore, we propose that TaBZR1, which participates in the BR pathway, and TaSAMT1-B, involved in the SA pathway, form a crosstalk and improve freezing tolerance under cold stress. In addition, the expression pattern of *TaSAMT1-B* in the *TaBZR1*-OE and *Tabzr1* mutant seedlings and the freezing tolerance phenotype of *TaBZR1*-OE and *Tabzr1* plants both support a function for TaBZR1 in cold tolerance (Fig. 4), in agreement with a previous study (Li et al. 2017).

Switching from repressed to active chromatin status in epigenetic regulation is one of the means employed by plants to survive in an ever-changing environment. As a major epigenetic regulatory mechanism, histone acetylation plays an important role in transcriptional regulation (Servet et al. 2010; Kim et al. 2018; Zhao et al. 2019; Lin et al. 2022; Song et al. 2022). Multiple studies have suggested histone acetylation as an additional layer of regulation for transcriptional reprogramming upon cold stress response (Chinnusamy et al. 2007; Zhu et al. 2008; Moraga and Aquea 2015; Park et al. 2018). In this study, we revealed that TaBZR1 physically interacts with the histone acetyltransferase TaHAG1 and demonstrated that the *TaSAMT1-B* locus is a direct downstream target of TaHAG1 and plays a critical role in cold tolerance of wheat (Figs. 6 and 7).

TaHAG1 associates directly with the *TaSAMT1-B* promoter and deposits increased H3 acetylation levels in response to freezing stress, providing further epigenetic regulatory evidence that TaHAG1 contributes to freezing tolerance. Accordingly, we hypothesize that *TaHAG1* expression upon freezing stress might induce a primed state characterized by enhanced histone acetylation at the promoter of cold-associated factors such as *TaSAMT1-B*, whose encoded protein then catalyzes more SA methylation to produce MeSA and improve freezing tolerance. Hence, we uncovered a regulatory mechanism between TaBZR1 and TaHAG1 for the regulation of *TaSAMT1-B* expression and freezing tolerance in wheat via TaBZR1 binding to the E-box motifs in the *TaSAMT1-B* promoter to recruit TaHAG1 and increase the levels of H3 acetylation.

It is worth noting that co-expression of *TaBZR1* with *TaHAG1* led to a significant increase in *TaSAMT1-B* promoter activation compared with the expression of *TaBZR1* alone, reflecting the coactivation function of TaHAG1. Therefore, on the basis of the current and previous work, we present the following model to illustrate the interaction between BR and SA signaling in wheat response to freezing stress. TaBZR1, the master regulator of the BR signaling pathway, binds to the E-box motif in the *TaSAMT1-B* promoter to induce *TaSAMT1* expression, which is further increased under freezing stress; the interaction of TaBZR1 with TaHAG1 is necessary for this enhanced transactivation (Fig. 7I). In addition, the *Tasamt1-B* mutants showed a lower tolerance to freezing stress that could not be rescued by exogenous BL application; mutation of *TaSAMT1-B* also suppressed the enhanced freezing stress tolerance of *TaBZR1*-OE seedlings (Fig. 5, F to H), suggesting that TaSAMT1-B functions downstream of the BR pathway and is important for BR-mediated freezing tolerance. Our findings also suggest that the synergistic mechanism between SA and BR pathways is more complex than what we revealed here. Future studies should include the identification of critical components with uncharacterized functions, analysis of functional redundancy between *TaSAMT1* homologous genes, and dissection of potential crosstalk at the protein level to fully address how the SA and BR signaling pathways are involved in freezing tolerance.

Depending on the duration and severity of cold stress, plants must carefully coordinate growth and stress responses. An overactive response may impose a fitness and yield penalty, although it is not clear if growth limitation represents an energy trade-off or is simply concomitant with stress responses (Nolan et al. 2020). Therefore, identifying the target genes and elucidating the underlying mechanisms that coordinate growth and stress responses are crucial for engineering crops with optimized stress responses. We measured the performance of the wild type, *TaSAMT1-B-*OE, and *Tasamt1-B* mutants throughout their entire growth period. We detected comparable yield-related traits under field conditions for wild-type, *TaSAMT1*-*B*-OE, and *Tasamt1-B* plants for spike length, spikelet number per spike, and thousand-kernel weight (TKW) (Supplementary Fig. S14). These results suggest that manipulation of *TaSAMT1* did not result in clear pleiotropic phenotypes or yield penalty under normal conditions but significantly increased freezing tolerance compared with wild-type plants under cold stress conditions. These findings suggest that *TaSAMT1* could serve as a target for both genetic engineering and selection for improvement of wheat cold tolerance. The regulatory factors or genes involved in cold tolerance identified in this study may also be harnessed for genetic improvement of other crops.

## Materials and methods

### Plant materials and phenotyping of wheat freezing tolerance

Wheat (*T. aestivum*) plants with different genotypes were cultivated in the experimental field in Beijing (39°57′N, 116°17′E) during the natural growing seasons. The seedlings of different genotypes with similar germination rate and vigor were grown in cultivation pots (4 × 4 × 6 cm, length × width × depth) filled with uniformly mixed soil (Pindstrup turf to vermiculite in a v/v ratio of 1:1) in a greenhouse at a relative humidity of 70% and 26/20°C day/night temperatures, with a 16-h-light/8-h-dark photoperiod and a light intensity of 3,000 lux (Master GreenPower CG T 400W E40; Philips). Ten-day-old seedlings after germination were exposed to low-temperature conditions (−10°C) for 1.5 h and subsequently allowed to recover at 22°C for 24 h. The number of surviving seedlings with green and viable stems was recorded. Phenotypes were evaluated by survival rates. At least 15 seedlings for each line were assessed in each test; statistical analyses were based on data obtained from three independent experiments.

The *N. benthamiana* plants, used as host plants, were grown in a greenhouse at 24 to 25°C under a 16-h-light/8-h-dark cycle for 4 to 6 weeks before agroinfection. The *Arabidopsis* Col-0 plants were grown in a greenhouse at a relative humidity of 70% and 22/18°C day/night temperatures, with a 16-h-light/8-h-dark photoperiod for 3 weeks before total RNA extraction.

### Electrolyte leakage assay

The relative electrolyte leakage (REL) assays were performed as described previously (Shi et al. 2012; Li et al. 2017). After freezing treatment, seedlings were placed into 15-mL tubes containing 10 mL of deionized water, whose electrical conductivity (EC) was S0, and then shaken for 15 min at 22°C to measure their EC as S1. After determining S1, the samples were boiled for at least 15 min and shaken at 22°C for another 20 min before their EC values were measured again, giving S2. REL was calculated as (S1–S0)/(S2–S0).

### RNA extraction and RT-qPCR

Ten-day-old seedlings were grown under normal conditions and treated at −10°C for 2, 4, 6, or 8 h, with one more sample collected just before freezing treatment as a 0-h sample. Total RNA was extracted, followed by reverse transcription using HiScript II Q RT SuperMix (Vazyme, China). RT**-**qPCR was performed with Taq Pro Universal SYBR qPCR Master Mix (Vazyme, China) on a C1000 Touch Real-Time PCR system (Bio-Rad, USA) to detect the expression of specific genes.

### Wheat protoplast isolation and transfection

Protoplasts were prepared from the wheat variety Fielder. Wheat protoplast isolation and transfection were performed as described (Wang et al. 2014). The Wizard Plus Midipreps DNA Purification System (Promega, USA) was used to extract plasmid DNA for protoplast transfection. Each plasmid (5 *µ*g per construct) was introduced by polyethylene glycol (PEG)–mediated transfection. The transfected protoplasts were incubated at 22°C for 14 to 16 h.

### Subcellular localization

The full-length coding sequence of *TaSAMT1-B* was cloned into *Xba*I-digested pCAMBIA1300-GFP vector using a Seamless Cloning and Assembly Kit (Biomed, China) to generate a fusion construct named pCAMBIA1300-GFP*-*TaSAMT1-B. The resulting vector and the empty vector (as control) were then separately transfected into wheat protoplasts as described above. After incubation at 23°C for 14 to 16 h in the dark, GFP and chlorophyll fluorescence were visualized using a confocal microscope (LSM880; Carl Zeiss; laser, 488 nm; intensity, 0.98%; collection bandwidth, 490 to 537 nm; gains: 805 v) and quantified using the image analysis software provided by the manufacturer.

### Sequence alignment and phylogenetic analysis

To perform phylogenetic analysis, the amino acid sequences of SAMT1 and BZR1 from different plant species were downloaded from Ensembl-plants (http://plants.ensembl.org/). MEGA 7 was employed to construct the phylogenetic tree with the following programs: all protein sequences were aligned in Clustal W with default parameters; then the maximum likelihood (ML) method was used to build a phylogenetic tree with the Poisson model and 1,000 bootstrap replicates.

### Production of recombinant TaSAMT1 and AtBSMT1 and enzyme assay

The full-length coding sequences of *TaSAMT1-B*, *TaSAMT1-A*, *TaSAMT1-D*, and *AtBSMT1* were individually amplified from the cDNA prepared from total RNA extracted from seedling leaves of Fielder variety and Arabidopsis (*Arabidopsis thaliana*) accession Col-0 using specific primers (Supplementary Data Set 3) and 2×Hieff Canace Gold PCR Master Mix (Cat No.10149; Yeasen, Shanghai, China). The amplicons were cloned into *Bam*HI-digested pGEX-6P-1 vector with a Seamless Cloning and Assembly Kit (Biomed, China). *Escherichia coli* Rosetta-gami2 (DE3) cells were transformed with each resulting construct. The production of each recombinant protein was induced with the addition of 0.1 mM isopropyl β-d-1-thiogalactopyranoside (IPTG) and incubated at 16°C for 16 h. The cells were collected by centrifugation at 6,297 × *g* for 5 min at 4°C and then resuspended and sonicated (39% amplitude, 20 min, 4°C) in lysis buffer (1× phosphate-buffered saline [PBS]). The supernatant from the resulting cell lysate was used as the crude enzyme extract. The reaction mixture for the enzyme activity assays comprised the following components: 4 mM MgCl_2_; 250 *μ*M SAM (S-adenosyl-L-methionine); 100 *μ*M adenosine triphosphate; 4 mM dithiothreitol; 1 mM substrates SA; and 900 *μ*L crude proteins. The mixture was adjusted to a final volume of 1.0 mL and then incubated at 28°C for 1 h. The activity of recombinant proteins was analyzed using a gas chromatography–mass spectrometry (GC-MS) instrument (Agilent 7250). Briefly, 1 *μ*L of each reaction mixture was injected into the GC-MS instrument and analyzed following the GC-MS temperature program: 50°C for 5 min, increased to 250°C at a rate of 10°C/min, and maintained for 2 min. Primer sequences are listed in Supplementary Data Set 3.

### Plasmid construction and plant transformation

To obtain knockout constructs for the candidate gene, the target sequences for the single guide RNAs (sgRNAs) were selected according to the specific region within the first or second exon using the E-Crispr Design Website (www.e-crisp.org/E-CRISP/designcrispr). The corresponding sgRNA sequences were synthesized (BGI, China), digested, and inserted into the binary vector pBUE411 via T_4_ DNA ligase (M0202, New England Biolabs, USA). The full-length coding sequence of *TaSAMT1-B* was amplified from the cDNA of seedling leaves of Fielder and cloned into *Bam*HI-digested pMWB110 vector to obtain the *proUbi:TaSAMT1-B* construct. The resulting vectors harboring the desired inserts were transformed into Agrobacterium (*A. tumefaciens*) strain EHA105 and transformed into wheat cultivar Fielder by Agrobacterium-mediated transformation. The primers used for the identification of the knockout transgenic plants are listed in Supplementary Data Set 3.

### SA, MeSA, and BL treatment

Salicylic acid (SA; Yuanye Bio-Technology, China), methyl salicylate (MeSA; Macklin, China), and brassinolide (BL; Coolaber, China) were dissolved in ethanol to proper concentrations as storage solutions. Each chemical was applied evenly on the leaf surface of 10-day-old seedlings to fully evaporated before freezing stress treatment using an identical volume of solvent (ethanol) as a mock treatment. After freezing treatment, the seedlings were allowed to recover at 22°C for 24 h before measuring the survival rates of seedlings.

### SA and MeSA quantification

Ten-day-old seedlings of different genotypes were exposed to −10°C for the indicated times. All seedlings were then dried in a low-temperature freeze dryer (REVO-RV53B5); the resulting dry samples were triturated before the addition of solvent (1 ppm 70% [v/v] HPLC-grade methanol with the addition of the internal standard acyclovir and 0.5 ppm roxithromycin). The mixture was sonicated for 30 min, and the supernatant was filtered through a 0.22-μm filter. The filtrate was checked on an LC-electrospray ionization (ESI)-MS/MS system (HPLC, Shim-pack UFLC SHIMADZU CBM20A system; MS, Applied Biosystems 5500 Q-TRAP) as previously described (Chen et al. 2013). The detected metabolites were analyzed on a mass spectrometry platform (HPLC, Shim-pack UFLC SHIMADZU CBM20A system; MS, Applied Biosystems Triple TOF 5600). The metabolites were quantified using a previously described method (Chen et al. 2013).

### BR quantification

BR content was determined by Nanjing Convinced-test Technology Co., Ltd. Ten-day-old seedlings were exposed to −10°C for 6 h using seedlings not exposed to freezing as a 0-h control sample. The plant tissues were frozen with liquid nitrogen in a mortar and ground into powder. To each 15-mL tube containing 1 g frozen plant material, 8 μL of a 1-μg/mL working solution of internal standards was added, followed by the addition of 10 mL extraction solvent consisting of isopropanol/H_2_O/concentrated HCl (2:1:0.002, v/v/v). The tubes were placed on a shaker at a speed of 100 rpm for 30 min at 4°C. Dichloromethane (5 mL) was added to each sample and shaken for 30 min in a cold room at 4°C. The samples were centrifuged at 13,000 × *g* for 5 min at 4°C. After centrifugation, the solvent from the lower phase was transferred to a screw-cap vial using a Pasteur pipette. The solvent mixture was concentrated but not completely dried using a nitrogen evaporator with nitrogen flow. The samples were redissolved in 0.4 mL methanol. For each sample, 2 µL was injected into a reverse-phased C18 Gemini HPLC column for HPLC-ESI-MS/MS analysis.

### Yeast one-hybrid assays

The promoter fragment for *TaSAMT1*-B was amplified from the genomic DNA of Fielder and cloned into *Kpn*I/*Xho*I-digested pAbAi vector (BGI, China). The construct was linearized with the restriction enzyme *BstB*I and then transformed into yeast (*Saccharomyces cerevisiae*) Y1HGold cells to generate a bait-specific reporter strain. The transformants were selected on synthetic defined medium lacking Ura and Leu (SD −Ura −Leu) and containing 400 ng/mL aureobasidin A (AbA).

The full-length coding sequence of *TaBZR1* was cloned into the *Bam*HI/*Xho*I-digested pB42AD (pJG4-5) vector, yielding the construct pB42AD-TaBZR1. The *TaSAMT1-B* promoter from Fielder was cloned into the *Bam*HI/*Xho*I-digested pLACZi2*μ* vector. These two resulting plasmids were co-transformed into yeast strain EGY48. Y1H assays were performed according to the manufacturer's recommendations (Clontech, Japan). Transformants were selected on SD medium lacking Trp and Ura and containing X-gal (5-bromo-4-chloro-3-indolyl-β-D-galactopyranoside) for blue color development (Li et al. 2010). The primers for Y1H were listed in Supplementary Data Set 3.

### Transient dual luciferase reporter assay

The wild-type or mutated promoter sequences of *TaSAMT1* were cloned into *Bam*HI-digested pGreen II 0800-LUC vector to generate the reporter plasmids. The full-length coding sequences of *TaBZR1* and *TaHAG1* were individually cloned into *Xba*I-digested pUC18-35S vector to yield effector constructs. Relative LUC activity assays were performed in wheat protoplasts, with different combinations of constructs co-transfected. Wheat protoplasts were then incubated in darkness for 15 h at 22°C. For cold treatment, wheat protoplasts were incubated in darkness for 13 h at 22°C and then treated at 4°C for 2 h. The detection of firefly luciferase (LUC) and *Renilla* luciferase (REN) activity was performed as described previously (Zheng et al. 2021). Each sample consisted of three independent transfection reactions; the primers used for cloning are listed in Supplementary Data Set 3.

### Electrophoretic mobility shift assay

The full-length coding sequence of *TaBZR1* was cloned into *Eco*RI/*Sal*I-digested pCold-MBP vector using a Seamless Cloning and Assembly Kit (Biomed, China) to produce an MBP fusion protein. MBP alone was produced from the empty vector and purified for use as a negative control. For EMSA, a 30-bp promoter fragment of *TaSAMT1-B* containing one E-box motif was synthesized (BGI, China) as biotin-labeled probe (Supplementary Data Set 3). Unlabeled probes containing the wild-type or mutated E-box motif were used as competitors. The assays were erformed using a Light Shift Chemiluminescent EMSA Kit (20148; Thermo Fisher Scientific) following the manufacturer's instructions. Recombinant MBP-TaBZR1 or MBP was added to the reaction mixture (1× binding buffer, 50 ng poly(dl·dC), 2.5% [v/v] glycerol, 0.05% [v/v] NP-40, 50 mM KCl, 5 mM MgCl₂, 2 mM EDTA [pH 8.0], and 1 nM biotin-labeled probe), followed by incubation at 25°C for 20 min. The reaction products were separated by 6% PAGE and transferred to a nylon membrane; detection of biotin-labeled DNA was performed according to the manufacturer's instructions.

### Yeast two-hybrid assay

*TaBZR1* was cloned into pGBKT7 vector and used as a bait to screen a yeast two-hybrid library. Different combinations of constructs were co-transformed into yeast strain AH109. Transformants were selected on SD medium lacking Leu and Trp. Liquid cultures started from positive transformants were then serially diluted and plated onto SD medium lacking Leu, Trp, and His and containing 60 mM 3-amino-1,2,4-triazole (3-AT).

### Luciferase complementation imaging assays

The LCI assay was carried out between TaBZR1 and TaHAG1 as described previously (Song et al. 2022). Briefly, the full-length coding sequences of *TaBZR1* and *TaHAG1* were cloned into *Kpn*I/*Sal*I-digested pCAMBIA1300-nLUC (nLUC) or KpnI/BamHI-digested pCAMBIA1300-cLUC (cLUC) vector, respectively. The resulting constructs were transformed into Agrobacterium strain GV3101; Agrobacterium cultures were then resuspended in infiltration buffer to an OD at 600 nm of 0.2, mixed in a 1:1 ratio (v/v), and then infiltrated into the leaves of *N. benthamiana* plants. Firefly LUC signal was collected after 2 days using a cooled charge-coupled device camera (NightSHADE LB 985; Berthold Technologies, Bad Wildbad, Germany) after spraying 1 mM of D-luciferin onto the leaves (Coolaber; CL6928, China). For each assay, three independent leaves of the same plant were assessed for statistical analysis of relative LUC activity. The pCAMBIA1300 n-LUC and pCAMBIA1300 c-LUC vectors were used as negative controls.

### In vitro pull-down assay

The recombinant proteins GST-TaBZR1 and MBP-TaHAG1 were purified from *E. coli* BL21 star (DE3). 10 μL GST-TaBZR1 or GST was incubated with glutathione beads (20507ES10; YEASEN, China) at 4°C for 2 h, followed by incubation with MBP-TaHAG1 for 2 h. The mixture was collected by centrifugation at 500 × *g* for 5 min at 4°C, followed by washing with PBS five times. Proteins bound to glutathione beads were separated on 10% (w/v) SDS-PAGE and detected with anti-GST (1:5,000, HT601, TransGen Biotech, China) and anti-MBP antibodies (1:5,000, AE016; ABclonal, China).

### Co-IP assay

For the Co-IP assay, the *TaBZR1-GFP* and *TaHAG1-MYC* fusion constructs were prepared. The full-length coding sequences of *TaBZR1* and *TaHAG1* were individually cloned into *Xba*I-digested pCAMBIA1300-GFP or *Sma*I-digested pCAMBIA1300-MYC vector, respectively. Each construct was transformed into Agrobacterium strain GV3101 for infiltration of *N. benthamiana* leaves via Agrobacterium-mediated infiltration as described above. Total proteins were isolated from different infiltrated leaves and incubated with anti-GFP beads at 4°C for 4 to 6 h with gentle shaking. The beads were washed with five times before the immunoprecipitated proteins were eluted in IP buffer. Input and eluted protein were then analyzed by immunoblotting using anti-GFP (mouse, 1:2,000; Abclonal, AE012, China) or anti-c-MYC antibodies (mouse, 1:2,000; TransGen Biotech, HT101-01, China). Primer sequences are listed in Supplementary Data Set 3.

### BiFC assay

For the BiFC assays, the full-length coding sequences of *TaHAG1* and *TaBZR1* were amplified and cloned individually into *Bam*HI/*Xba*I-digested cYFP and nYFP vectors, respectively. The resulting constructs and the empty nYFPN and cYFP vectors were transformed into Agrobacterium strain GV3101 and mixed in the appropriate combinations for co-infiltration into *N. benthamiana* leaves. Two days later, the YFP fluorescence signals were detected with a confocal microscope (LSM880; Carl Zeiss, Heidenheim, Germany; laser, 488 nm; intensity, 0.98%; collection bandwidth, 490 to 598 nm; gains: 700 v).

### ChIP assay

ChIP assays were performed as described previously (Zheng et al. 2021). Briefly, 2 g of 10-day-old wheat seedlings was collected and subjected to vacuum infiltration in 1% (v/v) formaldehyde for 20 min at 25°C. After isolation and lysis of nuclei, chromatin was sonicated (20% amplitude, 30 min, 4°C) and incubated with anti-BZR1 antibody (ABclonal, China), anti-GFP antibody (Millipore, USA), anti-H3K9ac antibody, and H3K14ac antibody (Abcam, Britain). The immunoprecipitated DNA fragments were then purified, and the enriched DNA fragments were analyzed by qPCR. Amplified DNA from the chromatin fractions prior to antibody incubation were used as controls. The relative enrichment at each target site was normalized to the input sample. Relevant primer sequences are listed in Supplementary Data Set 3.

### Statistical analysis

The statistical significance of differences was evaluated by Student's *t* test and one-way analysis of variance (ANOVA) followed by Tukey's multiple comparison test (Supplementary Data Set 4). Asterisks indicate significant differences (*, *P* < 0.05; **, *P* < 0.01). Different lowercase letters indicate significant differences (*P* < 0.05).

### Accession numbers

The sequences for all wheat genes used in this study are available in the Ensemb-plants (https://plants.ensembl.org/Triticum_aestivum/Info/Index). The accession numbers of the key genes mentioned are as follows: *TaSAMT1-B* (TraesCS3B02G445100), *TaBZR1* (TraesCS2A02G187800), and *TaHAG1* (TraesCS1D02G134200).

## Supplementary Material

koae100_Supplementary_Data

## Data Availability

The data underlying this article are available in the article and in its online supplementary material.

## Supplementary data

The following materials are available in the online version of this article.

**Supplementary Figure S1.** Heatmap representation of the expression levels of *TaSAMT* family members at 4°C and 23°C (Supports Fig. 1).

**Supplementary Figure S2.** Phylogenetic analysis and protein sequence alignment of TaSAMTs with homologs in wheat, *Brachypodium distachyon*, barley, and rice (Supports Fig. 1).

**Supplementary Figure S3.** Multiple amino acid alignment and protein structure of the three TaSAMT1 homoeologs (Supports Fig. 1).

**Supplementary Figure S4.** Production of recombinant TaSAMT1-A, TaSAMT1-B, TaSAMT1-D, and AtBSMT1 fused to GST in *E. coli* (Supports Fig. 1).

**Supplementary Figure S5.** Phylogenetic analysis and protein sequence alignment of TaBZR1 and its homologs in different species (Supports Fig. 4).

**Supplementary Figure S6.** *TaBZR1* expression in response to freezing treatment and in wild-type KN199 and *TaBZR1*-OE lines (Supports Fig. 4).

**Supplementary Figure S7.** Identification of the *Tabzr1* mutant (Supports Fig. 4).

**Supplementary Figure S8.** Endogenous MeSA levels in wild-type KN199 and *TaBZR1*-OE plants under freezing treatment (Supports Fig. 4).

**Supplementary Figure S9.** Relative transcript levels of *TaBZR1* and *TaSAMT1* in response to BL treatment (Supports Fig. 5).

**Supplementary Figure S10.** The interaction of TaBZR1 with TaHAG1 is specific (Supports Fig. 6).

**Supplementary Figure S11.** Relative *TaHAG1* transcript levels in response to freezing treatment and in wild-type Fielder and *TaHAG1-*OE lines (Supports Fig. 6).

**Supplementary Figure S12.** Endogenous MeSA levels in wild-type Fielder and *TaHAG1*-OE plants under freezing treatment (Supports Fig. 6).

**Supplementary Figure S13.** Enrichment of TaBZR1, H3K9ac, and H3K14ac at *TaSAMT1-B* promoter regions in wheat under BL treatment (Supports Fig. 7).

**Supplementary Figure S14.** Agronomic traits of different *TaSAMT1* transgenic lines and wild-type Fielder plants grown in the field (Supports Figs. 2 and 7).

**Supplementary Data Set 1.** List of the upstream regulators of *TaSAMT1-B* expression identified in Y1H assays.

**Supplementary Data Set 2.** Candidate TaBZR1-interacting proteins identified by Y2H screening.

**Supplementary Data Set 3.** Primer pairs used in this study.

**Supplementary Data Set 4.** Data analysis.

## Dive Curated Terms

The following phenotypic, genotypic, and functional terms are of significance to the work described in this paper:

BZR1 Gramene: AT1G75080
BZR1 Araport: AT1G75080

BES1 Gramene: AT1G19350
BES1 Araport: AT1G19350

EIN3 Gramene: AT3G20770
EIN3 Araport: AT3G20770

NPR1 Gramene: AT1G64280
NPR1 Araport: AT1G64280

BAK1 Gramene: AT4G33430
BAK1 Araport: AT4G33430

BRI1 Gramene: AT4G39400
BRI1 Araport: AT4G39400

BSMT1 Gramene: AT3G11480
BSMT1 Araport: AT3G11480

EIL1 Gramene: Os03g20790
EIL1 Araport: Os03g20790
