# Supplementary material for: Methyltransferase TaSAMT1 mediates wheat freezing tolerance by integrating brassinosteroid and salicylic acid signaling
Source: Plant Cell. 2024 Mar 27;36(7):2607–28. doi: 10.1093/plcell/koae100 (PMC11218785; doi:10.1093/plcell/koae100)
Supplement: koae100_Supplementary_Data [file koae100_supplementary_data.zip › TPC2024RA00036DR1Supplementary Figures S114.pdf]

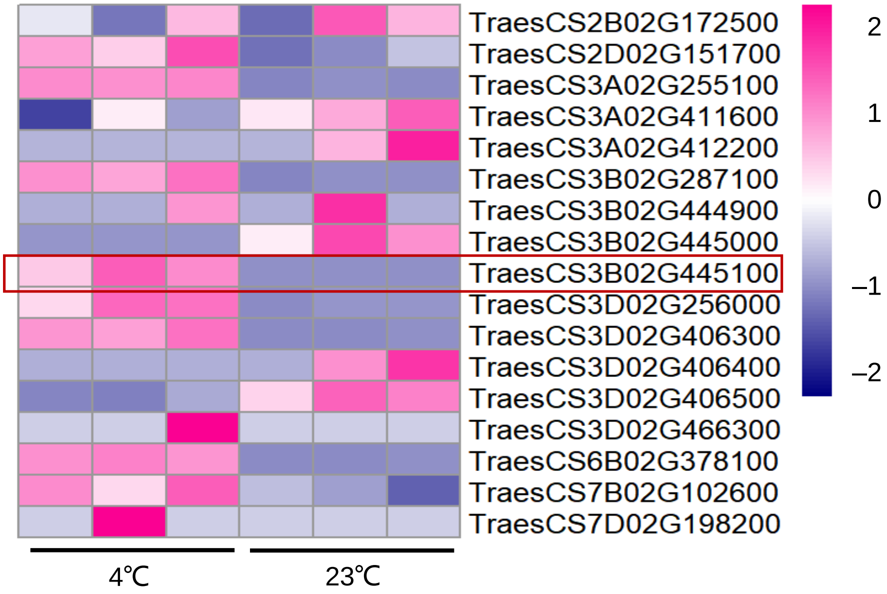

Supplemental Figure S1. Heatmap representation of the expression levels of *TaSAMT* family members at 4℃ and 23℃ (Supports Figure 1). The red box indicates target gene *TaSAMT1.*

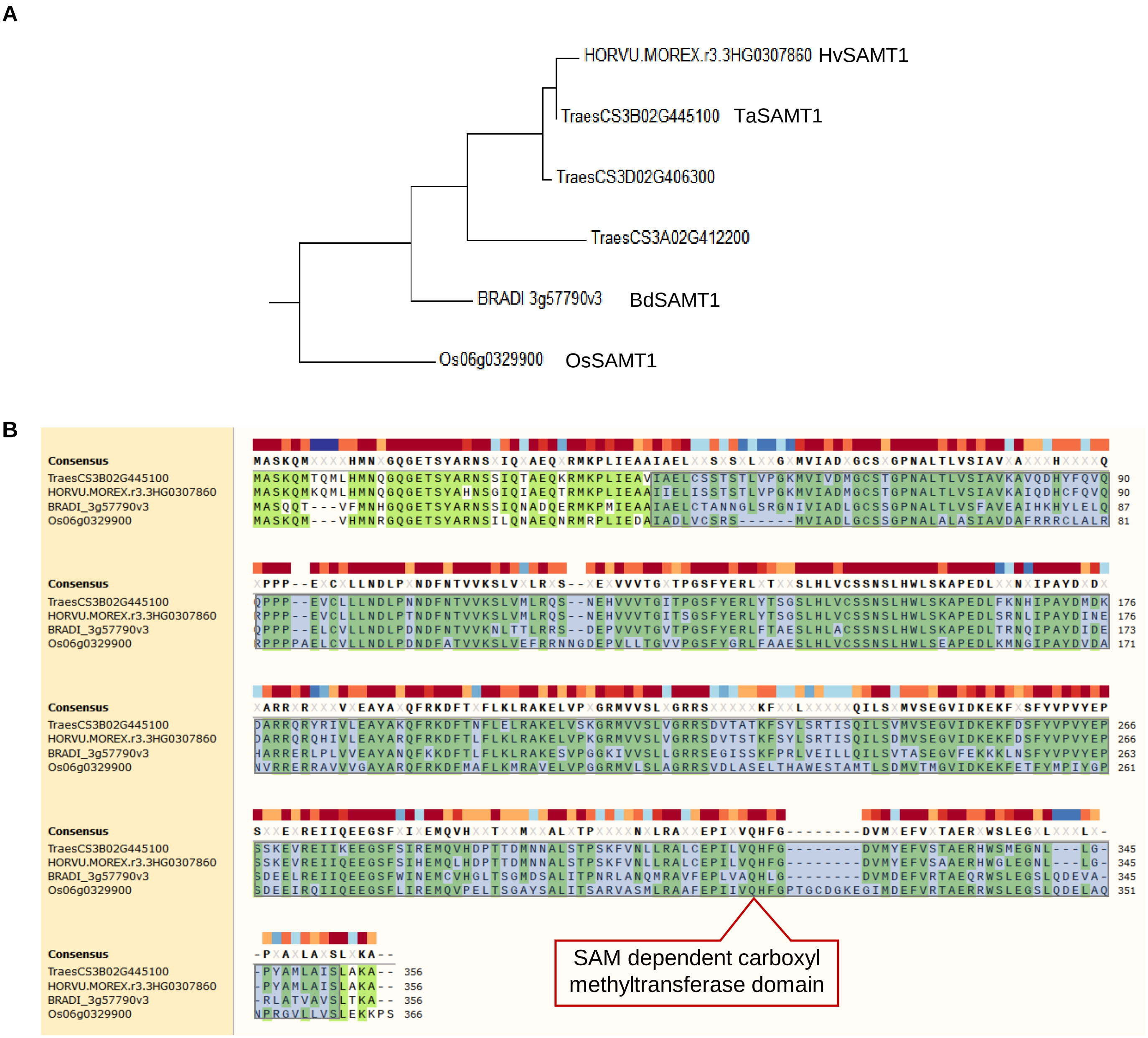

Supplemental Figure S2. Phylogenetic analysis and protein sequence alignment of TaSAMTs with homologs in wheat, *Brachypodium distachyon*, barley, and rice (Supports Figure 1).

(A) Phylogenetic of TaSAMTs with orthologs in *Brachypodium distachyon*, barley, and rice. The maximum likelihood (ML) method is performed in MEGA 7.0 software. **(B)** Comparison the amino acids of TaSAMT with HvSAMT , BdSAMT and OsSAMT. Gray boxes indicate the SAM-dependent carboxyl methyltransferase domain.

A

```
TaSAMT1-A   ----------------------------------MKSLIEGVVVELCSNAGTLQPRKIVI
TaSAMT1-B   MASKQMTQMLHMNQGQGETSYARNSSIQTAEQKRMKPLIEAVIAELCSSTSTLVPGKMVI
TaSAMT1-D   ------------------------------------------------------------

TaSAMT1-A   ADLGCSTGPNALALVSIAVNAIHDHCLQFQQPSPEVSVLLNDLPENDFNTVVKSLVTLRQ
TaSAMT1-B   VDMGCSTGPNALTLVSIAVKAVQDHYFQVQQPPPEVCLLLNDLPNNDFNTVVKSLVMLRQ
TaSAMT1-D   --------------------------------------------------------MLRQ
                                                                     ***

TaSAMT1-A   SNDPVVVTGITPGSFYERLFTSESVHLVCSSNSLHWLSKVRV------------------
TaSAMT1-B   SNEHVVVTGITPGSFYERLYTSGSLHLVCSSNSLHWLSKAPEDLFKNHIPAYDMDKDARR
TaSAMT1-D   SNEHVVVTGITPGSFYERLYTSDSLHLVCSSNSLHWLSKAPEDLFRNLIPAYDMDKDARR
            **: ***************:** *:**************.

TaSAMT1-A   ------------------------------------------------------------
TaSAMT1-B   QRYRIVLEAYAKQFRKDFTNFLELRAKELVSKGRMVVSLVGRRSDVTATKFSYLSRTISQ
TaSAMT1-D   QRHRLVLEAYAKQFRKDFTNFLKLRAKELVSKGRMVVSLVVRRSDVTATKFSYLSRTISQ

TaSAMT1-A   ------------------------------------------------------------
TaSAMT1-B   ILSVMVSEGVIDKEKFDSFYVPVYEPSSKEVREIIKEEGSFSIREMQVHDPTTDMNNALS
TaSAMT1-D   ILSVMVSEGVIDKEKFDSFYVPVYEPSSKEVRETIEEEGSFSIKEMQVHDPTTDMNNALS

TaSAMT1-A   ---------------------------------------------------------
TaSAMT1-B   TPSKFVNLLRALCEPILVQHFGDVMYEFVSTAERHWSMEGNLLGPYAMLAISLAKA
TaSAMT1-D   TPSKFVNLLRALCEPILVQHFGDVMYEFVSAAERHWSLEGNLLGPYAMLAISLAKA
```

SAM dependent carboxyl methyltransferase domain

*: identical amino acids among in the three TaSAMT1 homoeologs;

-: the missing amino acids in TaSAMT1-A or TaSAMT1-D compared with TaSAMT1-B

B

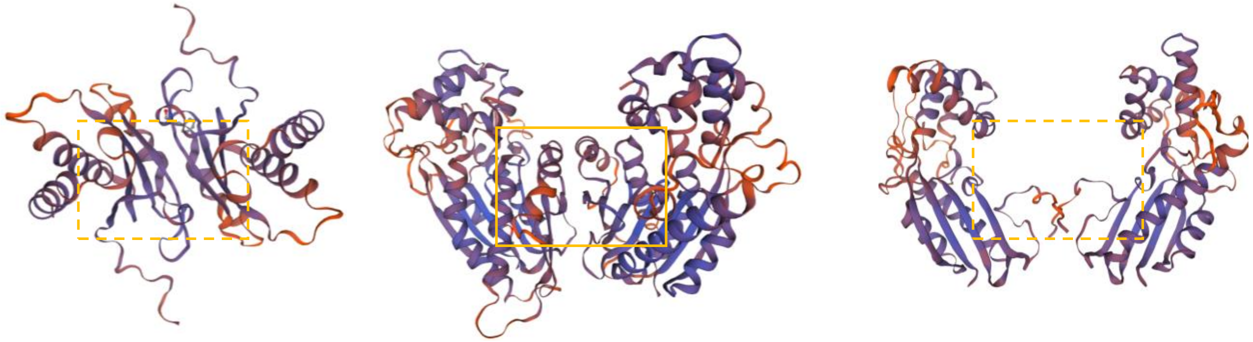

TraesCS3A02G412200
TaSAMT1-A

TraesCS3B02G445100
TaSAMT1-B

TraesCS3D02G406300
TaSAMT1-D

Supplemental Figure S3. Multiple amino acid alignment and protein structure of the three TaSAMT1 homoeologs (Supports Figure 1).

(A) Comparison the amino acids of TaSAMT1-A, TaSAMT1-B and TaSAMT1-D. Identical amino acids are indicated by asterisks, and gray boxes indicate the SAM-dependent carboxyl methyltransferase domain. **(B)** Protein structure prediction analysis of TaSAMT1-A, TaSAMT1-B and TaSAMT1-D. Yellow box indicates the putative catalytic region in TaSAMT1-B, that is broken both in TaSAMT1-A and TaSAMT1-D.

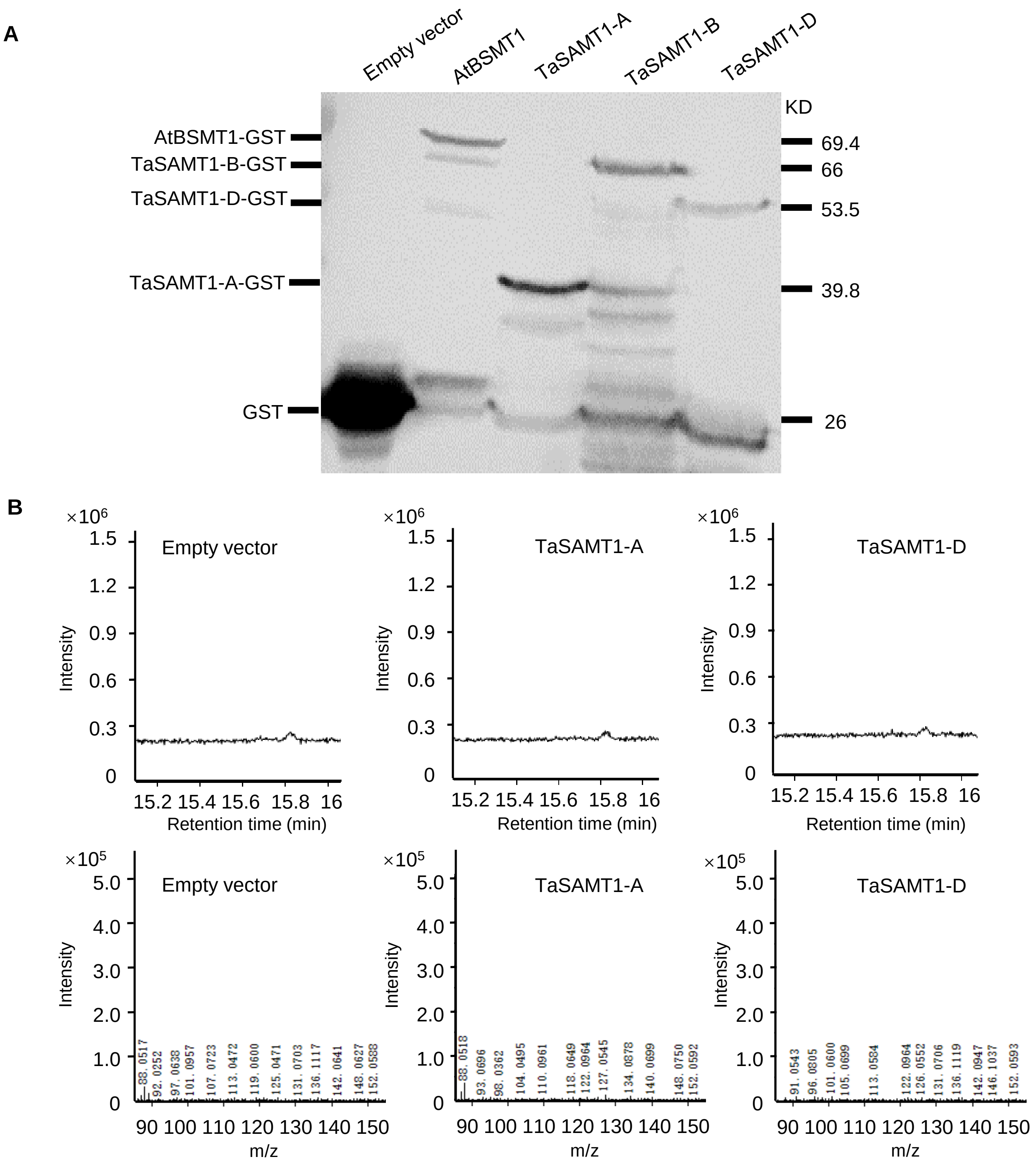

Supplemental Figure S4. Production of recombinant TaSAMT1-A, TaSAMT1-B, TaSAMT1-D, and AtBSMT1 fused to GST in *E. coli* (Supports Figure 1).

(A) The crude protein extracts from *E. coli* cell pellets were separated by SDS-PAGE and analyzed by immunoblotting with an anti-GST antibody (HT601-01; TransGen Biotech, China). **(B)** Enzyme activity assay using recombinant TaSAMT1-A-GST, TaSAMT1-D-GST, or GST as negative control, using SA as substrate.

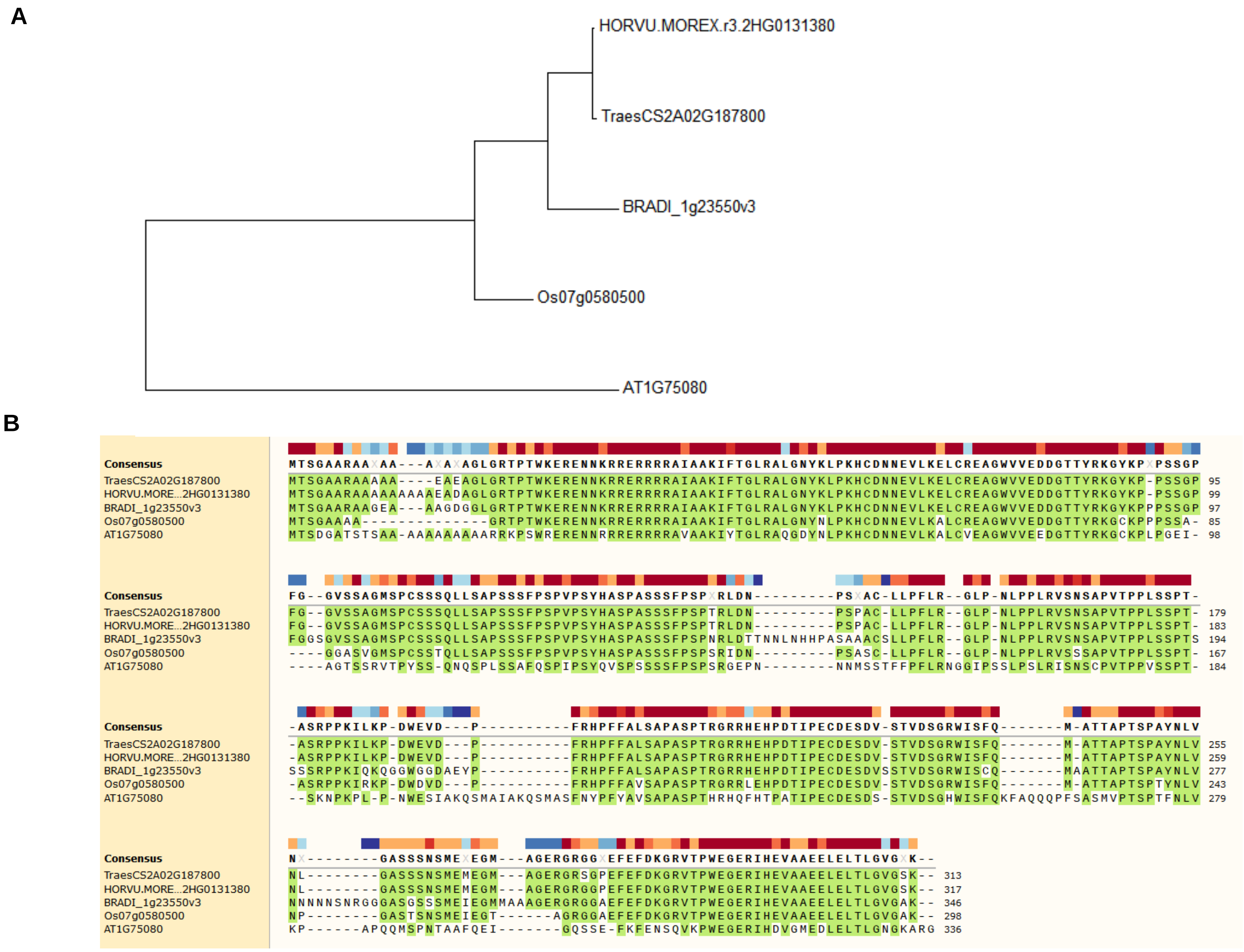

Supplemental Figure S5. Phylogenetic analysis and protein sequence alignment of TaBZR1 and its homologs in different species (Supports Figure 4).
(A) Phylogenetic analysis of TaBZR1 and its orthologs in different species. The maximum likelihood (ML) method is performed in MEGA 7.0 software. **(B**) Comparison the amino acids of TaBZR1 with its orthologs in different species.

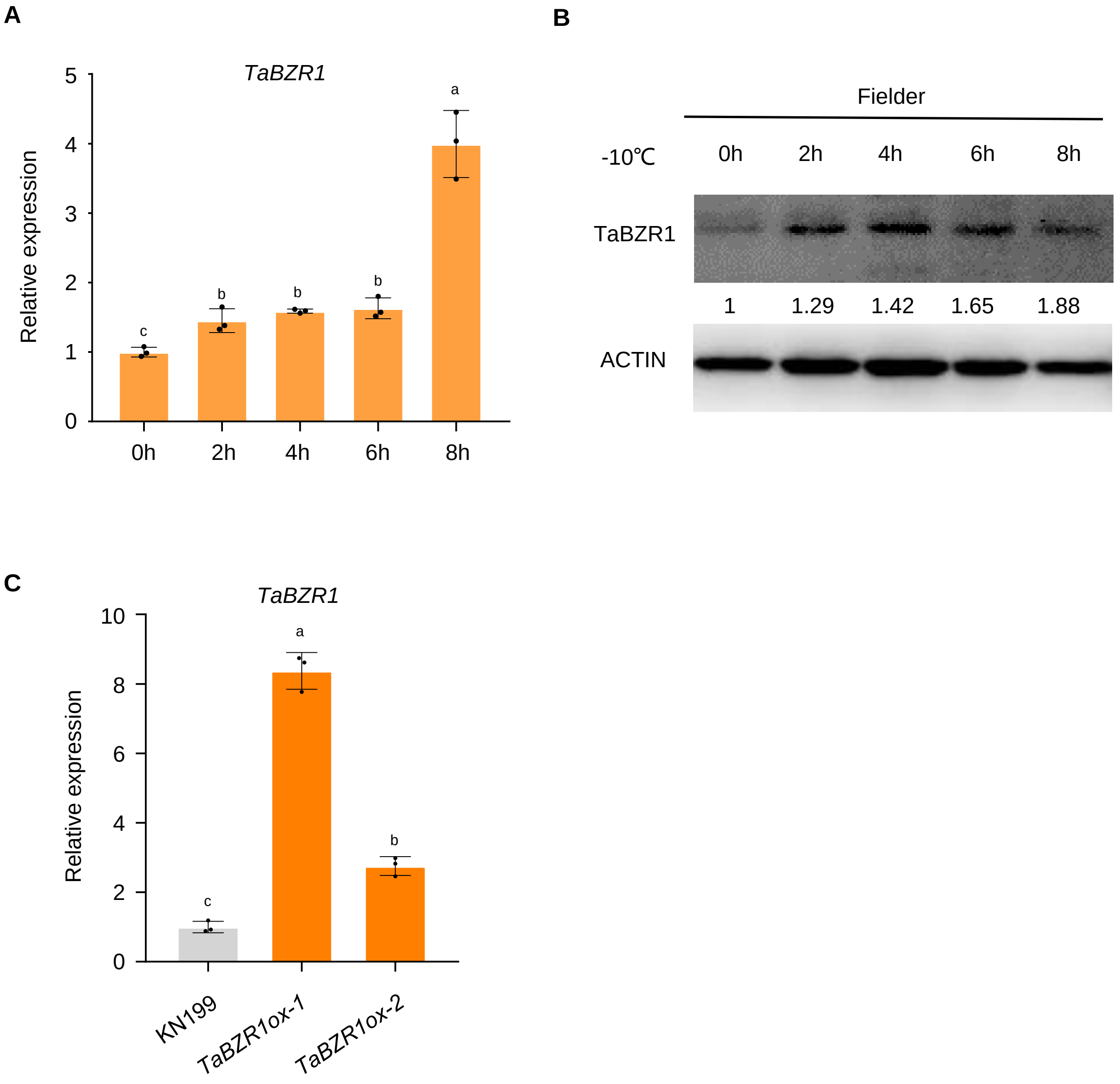

Supplemental Figure S6. *TaBZR1* expression in response to freezing treatment and in wild-type KN199 and *TaBZR1*-OE lines (Supports Figure 4).

(A) Relative *TaBZR1* transcript levels in response to treatment at −10℃ for up to 8 h. Data were normalized to *Ta-ACTIN* and are shown as means ± standard deviation (SD, n = 3). **(B)** Immunoblot analysis of TaBZR1 in Fielder under freezing stress. Ten-day-old seedlings grown in normal conditions were exposed to −10℃ for 2, 4, 6, or 8 h, or collected prior to cold treatment (0 h). Total proteins were extracted and subjected to immunoblot analysis with anti-BZR1 antibodies (ABclonal, China). **(C)** Relative *TaBZR1* transcript levels in wild-type (KN199) and *TaBZR1*-OE (ox-1 and ox-2) plants. Data are shown as means ± SD (n = 3). The data in **(A)** and **(C)** were analyzed by one-way ANOVA followed by Tukey's test. Different lowercase letters indicate a significant difference ($P < 0.05$).

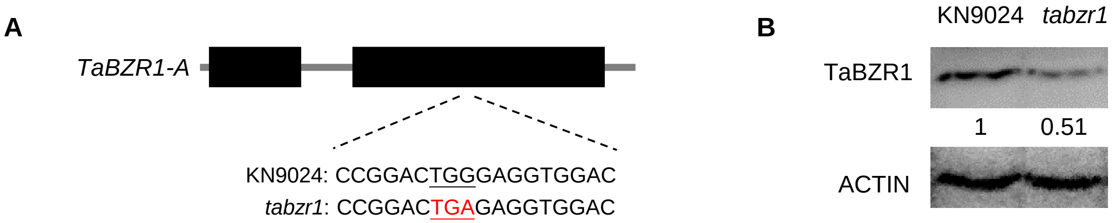

Supplemental Figure S7. Identification of the *Tabzr1* mutant (Supports Figure 4).
(A) *TaBZR1* sequences in the wild type (KN9024) and *tabzr1* mutant. The mutant carries a G-to-A transition in the second exon of *TaBZR1-A*. **(B)** Immunoblot analysis of TaBZR1 abundance with an anti-BZR1 antibody in the *Tabzr1* mutant and wild type plants. Total proteins were extracted and subjected to immunoblot analysis with anti-BZR1 antibodies (ABclonal, China).

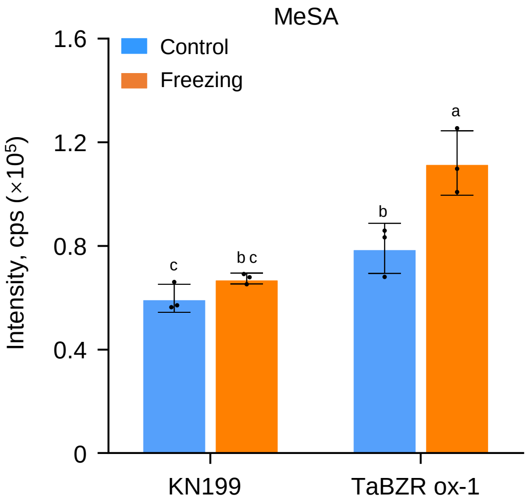

Supplemental Figure S8. Endogenous MeSA levels in wild-type KN199 and *TaBZR1*-OE plants under freezing treatment (Supports Figure 4). Data are shown as means ± SD (n = 3). The data were analyzed by one-way ANOVA followed by Tukey's test. Different lowercase letters indicate a significant difference ($P < 0.05$).

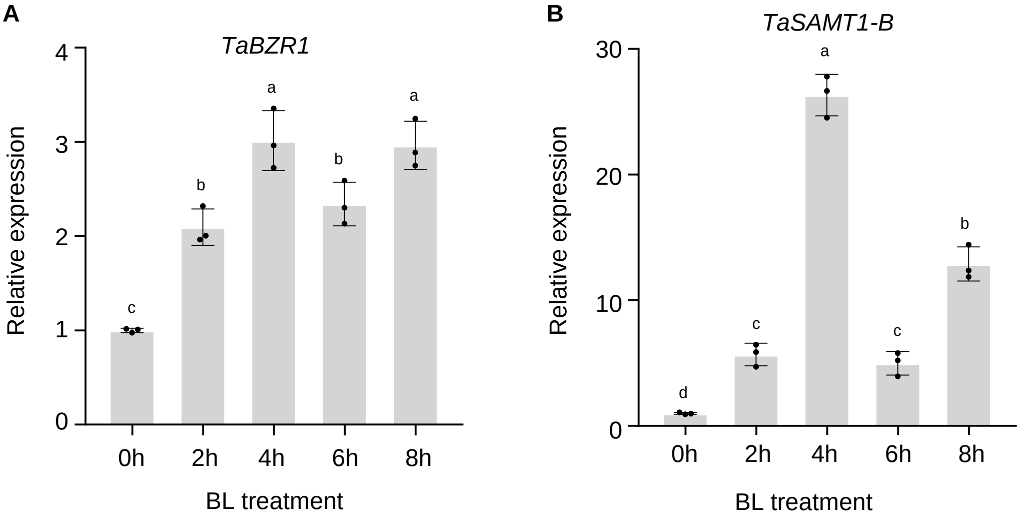

Supplemental Figure S9. Relative transcript levels of *TaBZR1* and *TaSAMT1* in response to BL treatment (Supports Figure 5). Data were normalized to *Ta-ACTIN* and are shown as means ± standard deviation (SD, n = 3). The data in **(A)** and **(B)** were analyzed by one-way ANOVA followed by Tukey's test. Different lowercase letters indicate a significant difference ($P < 0.05$).

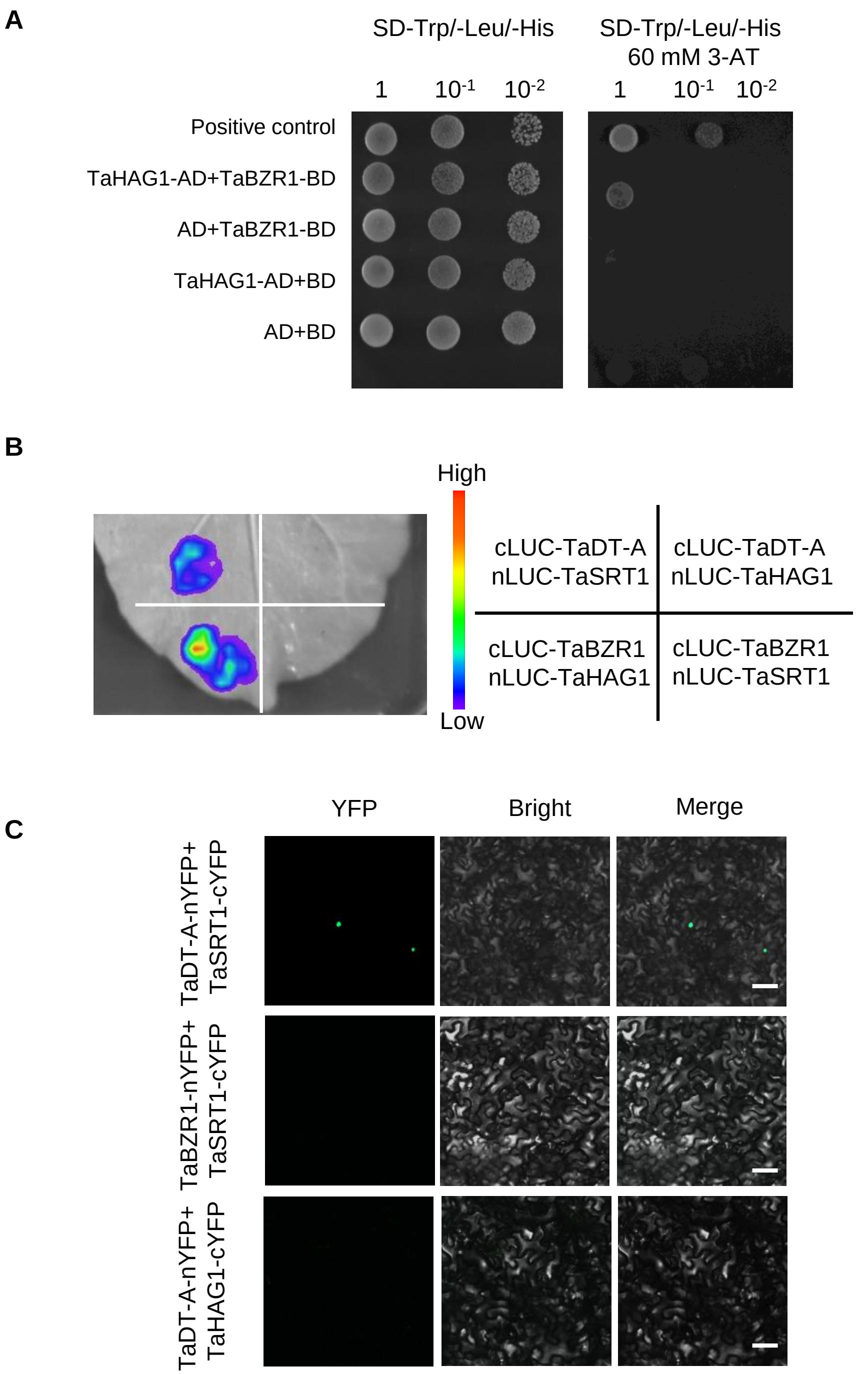

Supplemental Figure S10. The interaction of TaBZR1 with TaHAG1 is specific (Supports Figure 6).
(A) Yeast two-hybrid assay showing the interaction of TaBZR1 and TaHAG1. **(B)** LCI assay shows that TaBZR1 cannot interact with TaSRT1 and TaHAG1 cannot interact with TaDT-A, both are as the negative controls. **(C)** BiFC assay in *N. benthamiana* leaf cells between TaBZR1 and TaSRT1, TaDT-A and TaHAG1. BF, bright field. Bar, 50 μm.

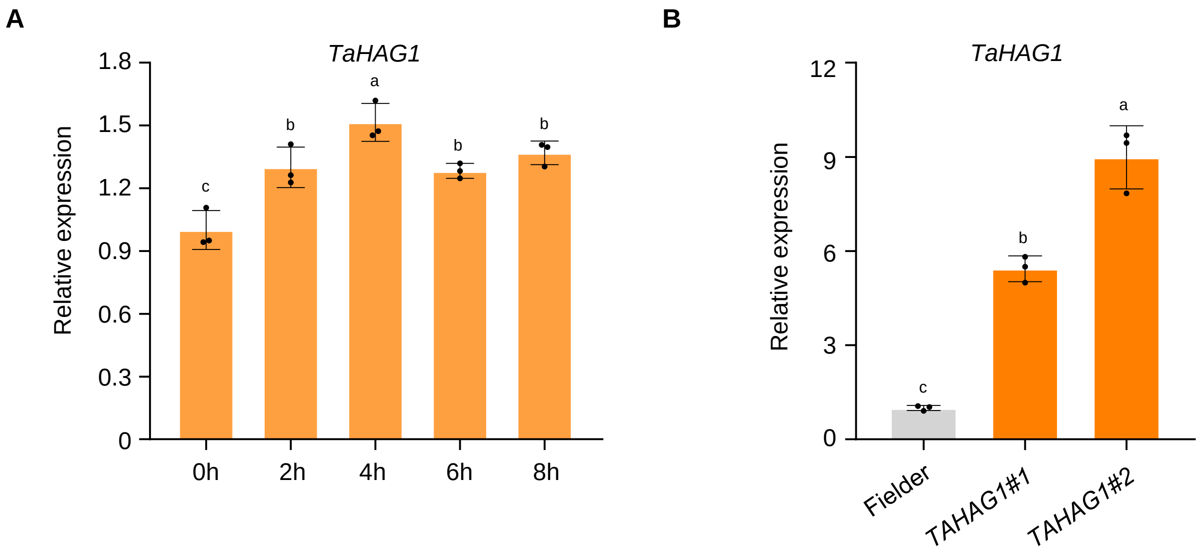

Supplemental Figure S11. Relative *TaHAG1* transcript levels in response to freezing treatment and in wild-type Fielder and *TaHAG1*-OE lines (Supports Figure 6).

(A) Relative *TaHAG1* transcript levels in response to treatment at −10℃ for up to 8 h. Data were normalized to *Ta-ACTIN* and are shown as means ± standard deviation (SD, n = 3). **(B)** Relative *TaHAG1* transcript levels in wild-type (Fielder) and *TaHAG1*-OE (#1 and #2) plants. Data are shown as means ± SD (n = 3). The data in **(A)** and **(B)** were analyzed by one-way ANOVA followed by Tukey's test. Different lowercase letters indicate a significant difference ($P < 0.05$).

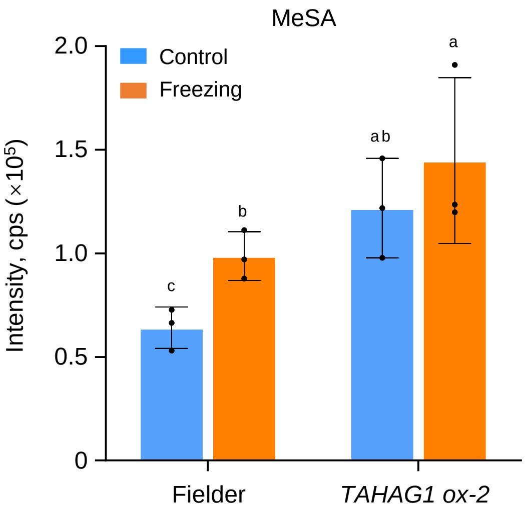

Supplemental Figure S12. Endogenous MeSA levels in wild-type Fielder and *TaHAG1*-OE plants under freezing treatment (Supports Figure 6). Data are shown as means ± SD (n = 3). The data were analyzed by one-way ANOVA followed by Tukey's test. Different lowercase letters indicate a significant difference ($P < 0.05$).

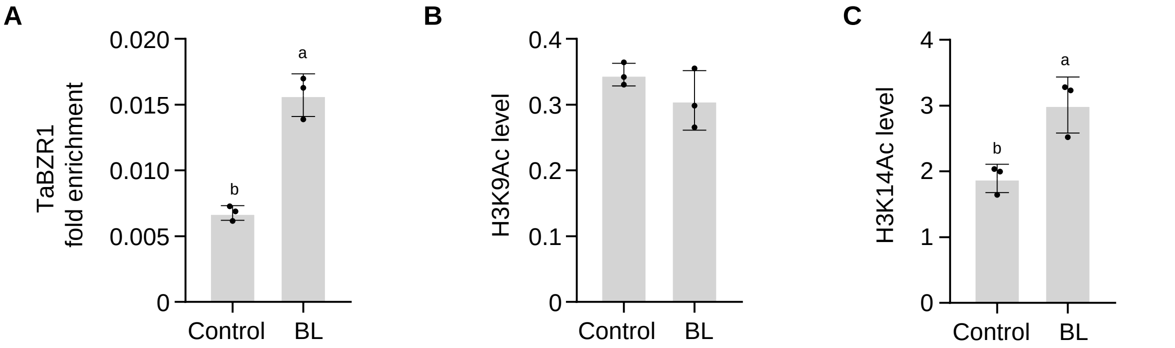

Supplemental Figure S13. Enrichment of TaBZR1, H3K9ac, and H3K14ac at *TaSAMT1-B* promoter regions in wheat under BL treatment (Supports Figure 7). The data in **(A)**, **(B)** and **(C)** are shown as means ± SD (n = 3) , and were analyzed by one-way ANOVA followed by Tukey's test. Different lowercase letters indicate a significant difference (P < 0.05).

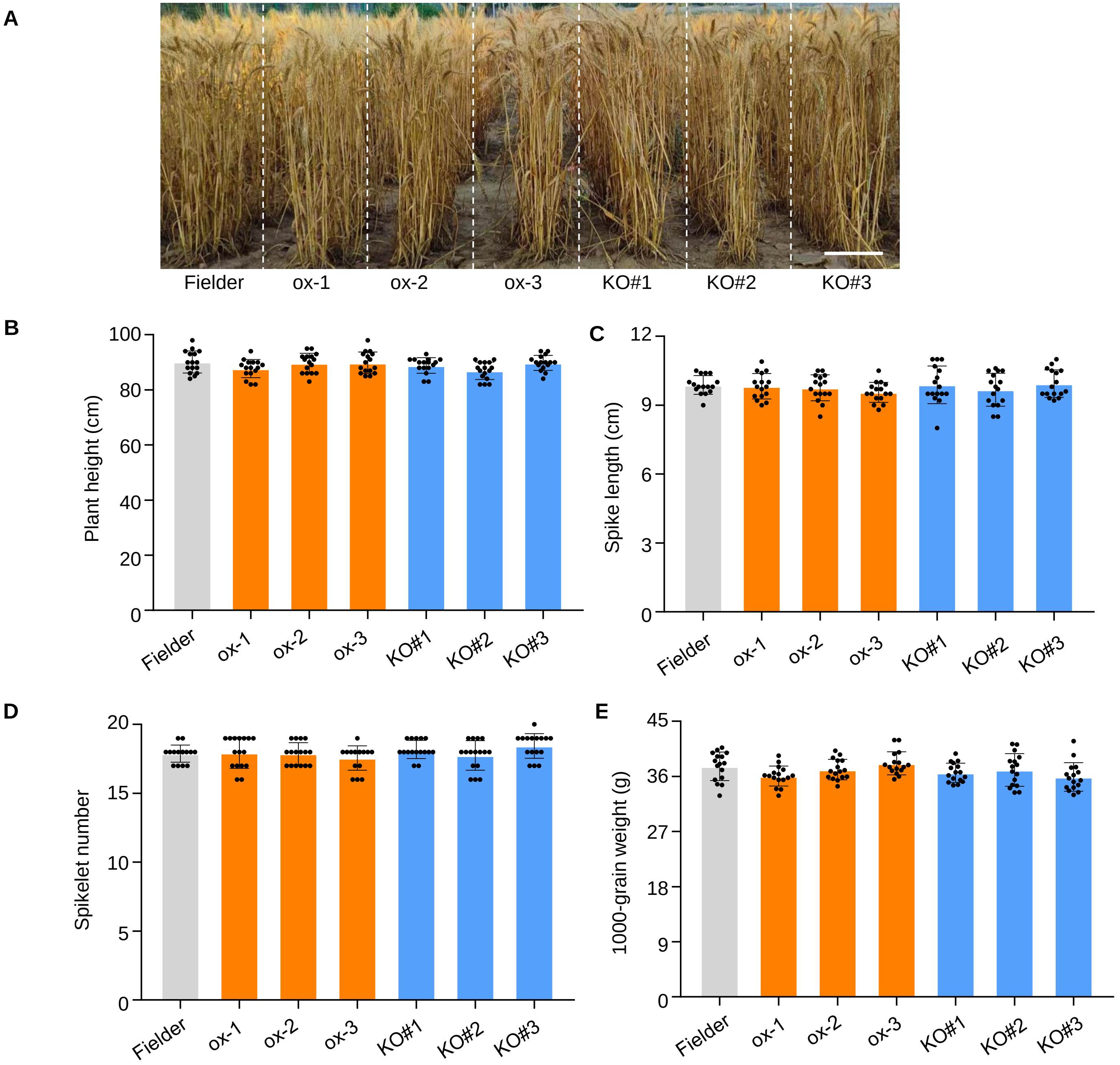

Supplemental Figure S14. Agronomic traits of different *TaSAMT1* transgenic lines and wild-type Fielder plants grown in the field (Supports Figures 2, 7).

(A) The mature plant of wild-type Fielder, *TaSAMT1-B*-OE (ox-1, ox-2, and ox-3), and *Tasamt1*-B knockout (KO#1, KO#2, and KO#3) lines. Scale bars, 20 cm. Analysis the yield-related traits under field conditions for wild-type, *TaSAMT1-B*-OE (ox-1, ox-2, and ox-3), and *Tasamt1*-B knockout (KO#1, KO#2, and KO#3) plants for plant height **(B)**, spike length **(C)**, spikelet number per spike **(D)**, and thousand-kernel weight (TKW) **(E)**. The data in **(B)**, **(C)**, **(D)**, and **(E)** are shown as means ± SD (n = 16).
