# Supplementary material for: Methyltransferase TaSAMT1 mediates wheat freezing tolerance by integrating brassinosteroid and salicylic acid signaling
Source: Plant Cell. 2024 Mar 27;36(7):2607–28. doi: 10.1093/plcell/koae100 (PMC11218785; doi:10.1093/plcell/koae100)
Supplement: koae100_Supplementary_Data [file tpc.00036.2024-s09.pdf › tpc.00036.2024-s09.pdf]

THE PLANT CELL

The salicylic acid methyl transferase TaSAMT1 mediates freezing tolerance by integrating the brassinosteroid and salicylic acid pathways in wheat

Wei Chu, Shumin Chang, Jingchen Lin, Chenji Zhang, Jinpeng Li, Xingbei Liu, Zehui Liu, Debiao Liu, Qun Yang, Danyang Zhao, Xiaoyu Liu, Weilong Guo, Mingming Xin, Yingyin Yao, Huiru Peng, Chaojie Xie, Zhongfu Ni, Qixin Sun, and Zhaorong Hu

Review Timeline:

Submission Date:	27-Mar-2023
Editorial Decision:	08-May-2023
Resubmission Received:	16-Jan-2024
Editorial Decision:	01-Feb-2024
Revision Received:	22-Feb-2024
Accepted:	23-Feb-2024

Dr. Zhaorong Hu
China Agricultural University
Beijing
China

Dear Dr. Hu:

Thank you for choosing to send your manuscript entitled "Salicylic acid methyl transferase TaSAMT1 mediates cold tolerance by integrating with the brassinosteroids and salicylic acid pathways in wheat" for consideration at The Plant Cell. Your submission has been evaluated by members of the editorial board as well as expert reviewers in your field, and we regret to inform you that we are not able to recommend the publication of this manuscript in its current form. We have had input from multiple scientists and we have solicited post-review comments.

During the post-review consultation session, we also agreed that if you could address the major points raised by the reviewers by new experiments, we would welcome a resubmission. This will be treated as a new submission, but we would attempt to use at least some of the same reviewers. Nevertheless, reviewers will be asked to assess as a new manuscript (i.e. are the claims fully supported by the data and do the results presented move the field forward?), and not only whether previous reviewer comments have been addressed.

The most important reviewer and editor comments that should be addressed are listed below:

- The manuscript should deal with freezing tolerance.
- A major conclusion of the work is that BR rescues cold tolerance phenotypes of the KO lines. To fully support this conclusions, it is important to show the cold tolerance phenotypes and TaSAMT1 gene expression profiles of the TaBZR1 KO mutant.
- The authors also assert that BZR1-mediated cold tolerance occurs via transcriptional regulation of TaSAMT1. For this conclusion to be fully valid, the authors need to test the phenotype of the TaBZR1 KO line crossed with the TaSAMT1 OX line. This would clarify that the role of TaBZR1 in cold tolerance occurs through TaSAMT1 (and not other target genes).
- The manuscript states that cold conditions increase the histone acetylation of TaSAMT1, but the mechanism is unclear in fully connecting TaBZR1-TaHAG1 regulation to cold signalling/response (shown in the authors' nice model/diagram). Does cold temperature enhance interaction between TaBZR1 and TaHAG1? And/or does cold temperature increase TaBZR1 recruitment to TaSAMT1. Are MeSA levels increased in TaBZR1 OX and/or TaHAG1 OX plants
- There are numerous issues related to lack of controls, quantification and methods description, which need to be resolved.

In addition, we find that the writing suffers from some problems with grammar and awkward phrasing, and in general lacks clarity and conciseness. We strongly encourage the use of a professional editing service to ensure that the work is presented in the best manner and reaches the broadest possible audience. Several editing services have agreed to extend a discount to authors coming to their website via this ASPB page https://aspb.org/aspb-journal-submission-editing-services/.

It will be important to convince the editors and reviewers that the study adds significant new understanding(s) of mechanisms or processes, or otherwise represents a significant advance in the field, and that the major claims made are fully justified by the data presented. This includes careful consideration and explanation of the various controls used in experiments, the extent and manner of replication, and the statistical analyses used. Sampling methods and the nature of "biological replicates" should be described precisely (i.e. different plants, parts of plants, pooled tissue, independent pools of tissue, sampled at different times, etc.), along with a clear description of and rationale for any statistical analyses conducted. The reader should know exactly what was sampled; what forms the basis of the calculation of any means and other statistical variables and parameters reported. This is also necessary to ensure that proper statistical analysis was conducted.

The Plant Cell now requires authors to complete and submit an author revisions checklist upon submission of a revised manuscript. The aim of the checklist is to aid authors in preparing a high-quality manuscript, facilitate the review and assessment of revised manuscripts, and help to ensure that journal standards are maintained across the board. If your manuscript is accepted, the completed checklist will be published as supplemental material attached to the article online. Please download a copy of the checklist (a fillable PDF form) at this link, for submission with your revised manuscript: https://tpc.msubmit.net/html/Author_Revisions_Checklist.pdf.

Note also that supplemental materials should be restricted to large datasets and tables, presentation of replicates, and validation of reagents, methods, or genotypes. Any data that are used to support the main claims must be in the main manuscript. Supplemental figure legends must indicate what figure in the main manuscript is supported by the supplemental data presented.

We thank you for your interest in and support of The Plant Cell. We hope we will be able to render a more positive decision on future work.

Sincerely,

The Plant Cell Board of Editors

-- Reviewer comments:

Reviewer #1 (Comments for the Author):

In the manuscript, the authors start from the functional identification of SA methyltransferase TaSAMT1 in cold resistance and provided solid evidence supporting the role of TaSAMT1 in freezing tolerance, e.g., TaSAMT is induced by cold stress in expression, knockout of TaSAMT1 results in increased sensitivity to freezing, etc.. Then, the regulation of TaSAMT1 was further investigated by identifying transacting proteins through Y1H screening, and TaBZR1, a key component of BR signaling pathway, was brought to the forefront of authors. Further Y2H screening identified the TaBZR1 partner TaHAG1, one of histone acetyltranferases in wheat. Both TaBZR1 and TaHAG1 are induced in expression by freezing in wheat, and overexpression of either one of them increased cold tolerance of transgenic wheat. TaBZR1 was evidenced to binding TaSAMT1 promoter sequence containing the E-box by EMSA analysis, and the sequence was also evidenced to be targeted by TaHAG1, the interaction partner of TaBZR1. Finally the authors proposed the model on how TaSAMT1 mediates cold tolerance by integrating with the BR and SA pathways, i.e., BZR1 binds to E-box containing sequence of TaSAMT1 promoter to establish the basic transregulation system for activating TaSAMT1 expression and the interaction of TaHAG1 with TaBZR1 is necessary for the enhanced transregulation system via increasing histone acetylation. Higher TaSAMT1 levels in turn result in accumulation of MeSA and enhanced resistance to cold tolerance. The authors' work may provide the community of plant stresses with novel clues on how BR and SA signaling pathways crosstalk or cooperate to regulate plants' tolerance to cold stress, a very interesting work. However, the quality of current manuscript is not up to publishing on PC. The authors are encouraged to improve the manuscript by adding more experimental data as well as comprehensive revision.

The main concern includes:

1. The role of TaBZR1-TaHAG1 interaction in regulating the expression of TaSAMT1 is not clear. From Figure 7E, BZR1 and HAG1 promote on each other in activating the expression of TaSAMT1. Is TaBZR1 helpful to the recruitment of TaHAG1 to TaSAMT1, and vice versa ? Or other roles? More experiments are needed to answer these questions. For example, checking whether BR is induced by freezing, whether BR treatment and BZR1 overexpression induce the increasing of histone acetylation of TaSAMT1.
2. Because of the problem in (1), BZR1-HAG1 interaction need to be tested by Co-IP, the golden standard for protein-protein interaction up to now.

Other comments:
1. Cold stresses include chilling and freezing. The authors used -10°C to treat the materials. This condition belongs to freezing. Replace cold with freezing in the manuscript.
2. The authors claimed that they used Y1H to screen the transacting regulator of TaSAMT1, and used Y2H to screen the interaction partners of TaBZR1. However no information on how the screening was performed. Add essential description on this work, including vector, cDNA library in Materials and Methods. The initial data showing the candidate proteins from screening should be mentioned in text.
3. The authors claimed that overexpression of TaSAMT1 confers tolerance to cold stress without the occurrence of discernible pleiotropic phenotypes and yield penalty. The authors drew the conclusion just based on a photograph (Fig S8) without any quantitative analyses on the yield in the field. More quantitative data are needed to support the conclusion if the authors want to keep this statement in manuscript. Otherwise, adjust the statement to avoid overstating the value of current data.
4. Line 130 and 136. In wheat genome, there are three homologs of TaSAMT1, TaSAMT1-A, TaSAMT1-B and TaSAMT1-C. Is TraesCS3BG445100, the TaSAMT1-B? Please clear it in text.
5. qRT-PCR or RT-qPCR ? Both are used in the manuscript. Should be RT-qPCR, not qRT-PCR.
6. For description of the location of sequences, line 245，-679 to -387 bp (from left to right); line 331, -360 to -760 bp (from right to left). If not having special meaning for experiment designing, the same pattern should be used to avoid confusing readers.
7. Line 372-373, I really do not understand the following sentence: which carries... platforms.
8. Line 393-395, TaSAMT1 is involved in SA pathway upon .... From the author's work, the protein is involved in MeSA biosynthesis and may regulate the relative content of SA and MeSA. However, it is not known whether TaSAMT1 is directly involved in SA signaling pathway in wheat.
9. Line 416, "tolerance in plant" need the or a before the word plant. Or just use the plural, plants.
10. Line 417, "pathway" should be "pathways".
11. Line 494, 10-day-old seedlings, 10 days after germination or 10 days after sowing ? clear it.
12. Line 494, 1.5 h and and. The second "and" should be deleted.
13. Line 495-496, how are surviving and died seedlings are judged? State the standard clearly.
14. Line 513-515, add the reference citation for the protoplast transformation, or describe the method in detail.

15. Line 546-547, Yuanye, Macklin and Coolaber are names of the company? if yes, add the name of the country to which the company belongs.

Reviewer #2 (Comments for the Author):

Summary
Low temperature stress is a major environmental stress types which limits plant performance and crop yields. Many crops are affected and among them is wheat, which can be damaged by freezing stress in several major growing areas of the world. In this work it was aimed to identify factors that can increase the frost tolerance of this crop plant. A novel mode of resistance against frost is revealed, which relies on the cold-induction of the salicylic acid (SA) methyltransferase TaSAMT1-B, that catalyzes MeSA production. Evidence is provided that this is carried out via the brassinosteroid signaling component TaBZR1 and the histone acyltransferase TaHAG1.
These are novel and significant findings since a SA methyltransferase has not been implicated in frost tolerance as yet. Also, it had been unknown that MeSA production can be induced by BR signaling components and that BZR1 takes part.

Methodology
In the study gain and loss-of function mutants of TaSAMT1 were generated to assess differences in freezing tolerance to wild-type, using seedling survival rates and electrolyte leakage as phenotypic read-outs. This showed that plants with reduced TaSAMT1 expression were more susceptible to frost damage, whereas plants with increased TaSAMT1 expression had an increased resistance. Also, TaSAMT1oe plants accumulated more MeSA after cold stress exposure than wild-type whereas TaSAMT1ko plants accumulated less, showing that TaSAMT1 contributes to MeSA formation in response to cold.
It is then shown that TaBZR1, a BR-controlled transcription factor, can bind to the TaSAMT1 promoter and induce its expression in response to cold stress. For this purpose, in vitro and in vivo DNA binding studies were used and a TaBZR1oe line was generated and assessed. In TaBZR1oe plants frost tolerance and TaSAMT1 expression were increased, providing evidence that it acts as a positive regulator of the pathway. In addition, it is shown that TaBZR1 can directly interact with the histone acetyltransferase TaHAG1 and that this interaction promotes TaBZR1 activity in TaSAMT1 promoter binding. Since TaHAG1oe lines showed increased frost tolerance, TaHAG1 is positioned in the cold-TaBZR1+TaHAG1-TaSMT1-MeSA pathway.

Concerns
+) Is there a BR context of TaBZR1 activity in the cold-induction of TaSAMT1 expression, like your model in Fig. 7 indicates? What's the evidence for it?
+) Are MeSA levels increased in TaBZR1oe and/or TaHAG1oe plants?
+) I don't see the E-box in the R2 promoter region of TaSAMT1. The region highlighted in red in Fig. 4A doesn't seem to contain one?!
+) The manuscript would benefit from language editing.

Reviewer #3 (Comments for the Author):

Dear Authors/Editor,

In this study, Chu and colleagues identified TaSAMT1-B as a salicylic acid (SA) methyltransferase in wheat plants, which converts salicylic acid (SA) into MeSA. Examining connection of TaSAMT1 to abiotic stresses, they discovered that TaSAMT1 transcript levels are upregulated by cold temperature. This leads to more MeSA production to confer chilling tolerance in wheat plants. Interestingly, the authors discovered that wheat TaBZR1 transcription factor directly targets the upstream sequences of TaSAMT1 to regulate gene expression. Mechanistically, TaBZR1 interacted with the histone acetyltransferase TaHAG1 and the resulting histone acetylation was enhanced by cold stress. Using genetic analyses, Chu and co-authors attempted to conclude that brassinosteroid (BR) signaling in wheat also mediates cold tolerance by modulating MeSA metabolism and homeostasis. Overall, this paper proposes that TaSAMT1 represents a metabolic/regulatory node for SA-BR hormone signaling crosstalk during cold stress responses in plants.

This study is interesting since, to my knowledge, this is the first time that a functional SAMT ortholog in wheat has been identified and characterized. Importantly, the authors tried to mechanistically link SA and BR signaling in cold tolerance responses, when previous studies only focused on the individual contribution of each plant hormone pathway. Taken together, this study should be of general interest to a broad readership, especially plant scientists working in abiotic stress, crop physiology, hormone signaling and gene regulation.

To further improve the manuscript, there are major issues that the authors need to address. In particular, these are associated with experiments and/or controls that would robustly support the broad claims and conclusions of the paper.

1) The authors performed biochemical characterization of TaSAMT1 activity and its ability to generate MeSA. They show MeSA standard as the positive control and empty expression vector as the negative control. Because the GC mass spectrum of the MeSA standard is expectedly "cleaner" than the TaSAMT1-generated MeSA, it is important that more appropriate controls are used. For example, a good positive control would be using a confirmed plant SA methyltransferase (e.g. Arabidopsis BSMT1) and good negative controls would be the truncated homeologs TaSAMT1-A and TaSAMT1-C.

2) As shown in Figure 3, MeSA does not fully rescue the phenotypes of the KO lines in terms of survival rates and electrolyte leakage. It may be prudent if a range of MeSA concentrations could be used to determine which concentration leads to full rescue of KO lines, making them comparable to the WT Fielder. If this does not work, then potential reasons for the partially rescued phenotype should be indicated in the Discussion section.

3) A major conclusion of the paper is that TaBZR1 directly targets and regulate TaSAMT1 transcription. To robustly support this claim, I believe it is necessary to show in vivo BZR1 ChIP data.

4) Another major conclusion is that exogenous BR rescues cold tolerance phenotypes of the KO lines. However, looking at Figure 5B, the increased survival rate by BL addition is only statistically significant in KO #1 but not in KO#2 and KO#3 lines. In addition, BL treatment has a lesser effect than MeSA treatment (shown in Figure 3). To fully support their conclusions on the mechanistic connection between BR and SA in plant cold tolerance, and the requirement of BZR1 regulation, it is important to show the cold tolerance phenotypes and TaSAMT1 gene expression profiles of the TaBZR1 KO mutant.

5) The authors also assert that BZR1-mediated cold tolerance occurs via transcriptional regulation of TaSAMT1 and eventual MeSA generation. The current datasets are not fully sufficient to state this conclusions since one would need to cross and test a TaBZR1 KO line with the TaSAMT1 OX line. This would strongly show that the role of TaBZR1 in cold tolerance occurs through TaSAMT1 and not via other genes that it targets as a transcription factor.

6) Finally, the authors state that cold conditions increase the histone acetylation of the TaSAMT1 locus, but it is unclear whether this is due to enhanced interaction between TaBZR1 and TaHAG1 at colder temperatures and/or increased TaBZR1 recruitment to the target TaSAMT1 promoter region. I believe this needs more clarification to fully connect TaBZR1-TaHAG1 regulation to cold signaling/response.

Minor comments:

1) Line 55 onward: In the Introduction (second paragraph), there needs to be better reflection of the current literature on cold temperature regulation of plant hormone signaling and metabolism. What is currently known in wheat and other plant species, especially in relation to the SA and BR pathways?

2) Lines 76-77: It is important to note that a recent study has shown systemic transport of SA via the apoplast (see Lim et al. 2020; https://doi.org/10.1126/sciadv.aaz0478).

3) Lines 112-115: This could be restated for better clarity.

4) Materials and Methods: This section needs more information to facilitate future reproducibility. For example, please indicate the soil mix (and suppliers) and PPFD light settings for plant growth. Importantly, clearly outline the cloning steps (and kits used) for the following: (a) TaSAMT1 cloning to pCAMBIA1300-GFP, pGEX-6P-1, pLACZi2u and pGreen II 0800-LUC; (b) TaBZR1 cloning into pB42AD (pJG4-5), pUC18-35S and pCold-MBP; and (c) TaHAG1 cloning into pUC18-35S. Additionally, what protocol for protoplast isolation was used? Finally, please detail the protein purification steps as mentioned in the EMSA assay and in-vitro pull-down assay sections.

5) My comments focus on the science and not grammar, but I suggest that English proofreading (perhaps using Grammarly or professional copyediting services?) is needed before publication.

Comments on Figures:

• For all figure captions: Please indicate the number of reproducible independent experimental repeats performed for the various experiments.

• In addition, please clearly define n (are they individual plants? Individual leaves of the same plant?, etc.)

• For Figure 1A/B; Figure 2A/E/F; Figure 3A/B/D/E; Figure 4D/G/G/H/I; Figure 5B/C; Figure 6E/F/G/H; Figure 7B/C/D; Figure S4C; and Figure S5; Figure S7A/B; and Figure S8B/C/D/E: For statistical transparency, please show the individual data points in all data graphs.

- Figure 1D: This needs a higher-resolution image.

- Figure 2B and Figure 4C: Quantification of band intensities needed.

- Figure 2F left panel: Statistical analysis is missing.

- Figure S1C: Please label the protein sequences with the catalytic site and other important protein domains.

- Figure S2B: Please label the protein structures with the catalytic site and other important protein domains, highlighting what are missing in the A and C homeologs.

Dear Editor and Reviewers,

We would like to thank you for the thorough evaluation of the manuscript and for the valuable recommendations and suggestions. We are grateful for all your contribution, which help us a lot to improve the quality of the manuscript. Hereby, we would like to express our gratitude for your time and work. In the revised manuscript, all the raised issues have been addressed and revised according as requested. The following responses have been provided to address all comments of reviewers in detail.

Response to the editor:

In the revised manuscript, all the raised issues have been addressed and revised according as requested by the editor and reviewers. Briefly, the *Tabzr1* mutant line was identified and freezing tolerance phenotypes and *TaSAMT1* gene expression profiles of the *Tabzr1* mutant were examined. The SR and cell membrane stability were significantly lower in *Tabzr1* mutant plants than that of wild-type, respectively. The expression of *TaSAMT1* was reduced in *Tabzr1* mutant plants compared to wild type (detailed in lines 309-325 and Fig. 4J-M in revised version).

we crossed a *TaBZR1*-OE line to a *Tasamt1* mutant line and identified plants homozygous for the mutation of *TaSAMT1-B* and overexpressing *TaBZR1*. This *Tasamt1 TaBZR1*-OE line shared the same decreased survival rate and cell membrane integrity phenotypes as the *Tasamt1* mutant under freezing treatment, indicating that TaSAMT1-B functions downstream of TaBZR1 and modulates freezing tolerance (detailed in lines 350-357 and Fig. 5F,G,H in revised version).

Moreover, ChIP assays with an anti-BZR1 antibody confirmed that TaBZR1 binds directly to the E-box motif-containing segments within the *TaSAMT1-B* promoter regions. Specifically, freezing stress treatment led to an enrichment of the R2 region containing the E-box motif from the *TaSAMT1-B* promoter in the precipitated chromatin, in line with *TaSAMT1-B* elevated transcriptional activation compared to mock treatment (detailed in lines 297-302 and Fig. 4E in revised version). Furthermore, the levels of H3K9ac at the *TaSAMT1-B* promoter were significantly increased in *TaBZR1*-OE seedlings compared to the wild type under freezing

treatment; H3K14ac levels were significantly higher in *TaBZR1*-OE seedlings than in the wild type under both control and freezing stress conditions (detailed in lines 437-441 and Fig. 7F,G in revised version). In addition, we observed an enrichment for TaBZR1 at the *TaSAMT1-B* promoter and increased H3K14ac levels over the *TaSAMT1* promoter following BL treatment relative to control seedlings (detailed in lines 444-449 and Fig. S13 in revised version). Notably, these changes in acetylation levels were not caused by changes in the affinity between TaBZR1 and TaHAG1, as determined by LCI assays in *N. benthamiana* leaves co-expressing *cLUC-TaBZR1* and *nLUC-TaHAG1* exposed to control or cold stress conditions (detailed in lines 450-453 and Fig. 7H in revised version). We also found the endogenous level of MeSA content was significantly higher both in *TaBZR1*-OE and *TaHAG1*-OE lines than in wild-type plants, especially under freezing treatment (detailed in lines 326-327, lines 406-407 and Fig. S8, Fig. S12 in revised version). Collectively, these results indicate that TaBZR1 directly binds to the *TaSAMT1-B* promoter and further activates its transcription under cold stress by recruiting TaHAG1 and increasing the levels of histone H3 acetylation (detailed in Fig. 4; Fig. 7 and Fig. S8,S12,S13 in revised version).

In addition, the quantification and methods description were further detailed in the revised version. The manuscript was revised completely by professional editing service (Planteditors).

Response to the reviewers point-by-point:

Reviewer comments:

Reviewer: 1

In the manuscript, the authors start from the functional identification of SA methyltransferase TaSAMT1 in cold resistance and provided solid evidence supporting the role of TaSAMT1 in freezing tolerance, e.g., TaSAMT is induced by cold stress in expression, knockout of TaSAMT1 results in increased sensitivity to freezing, etc. Then, the regulation of TaSAMT1 was further investigated by identifying transacting proteins through Y1H screening, and TaBZR1, a key

component of BR signaling pathway, was brought to the forefront of authors. Further Y2H screening identified the TaBZR1 partner TaHAG1, one of histone acetyltranferases in wheat. Both TaBZR1 and TaHAG1 are induced in expression by freezing in wheat, and overexpression of either one of them increased cold tolerance of transgenic wheat. TaBZR1 was evidenced to binding TaSAMT1 promoter sequence containing the E-box by EMSA analysis, and the sequence was also evidenced to be targeted by TaHAG1, the interaction partner of TaBZR1. Finally, the authors proposed the model on how TaSAMT1 mediates cold tolerance by integrating with the BR and SA pathways, i.e., BZR1 binds to E-box containing sequence of TaSAMT1 promoter to establish the basic transregulation system for activating TaSAMT1 expression and the interaction of TaHAG1 with TaBZR1 is necessary for the enhanced transregulation system via increasing histone acetylation. Higher TaSAMT1 levels in turn result in accumulation of MeSA and enhanced resistance to cold tolerance. The authors' work may provide the community of plant stresses with novel clues on how BR and SA signaling pathways crosstalk or cooperate to regulate plants' tolerance to cold stress, a very interesting work. However, the quality of current manuscript is not up to publishing on PC. The authors are encouraged to improve the manuscript by adding more experimental data as well as comprehensive revision.

Response: We greatly appreciate your positive evaluation on the research topic. Thanks very much for your suggestion. We have performed additional experiments, further analyzed the data; and the manuscript was revised in the new version, accordingly. The followings are our detailed responses to your comments.

Major comments:

1. The role of TaBZR1-TaHAG1 interaction in regulating the expression of *TaSAMT1* is not clear. From Figure 7E, BZR1 and HAG1 promote on each other in activating the expression of *TaSAMT1*. Is TaBZR1 helpful to the recruitment of TaHAG1 to TaSAMT1, and vice versa? Or other roles? More experiments are needed to answer these questions. For example, checking whether BR is induced by freezing, whether BR treatment and BZR1 overexpression induce the increasing of histone acetylation

of TaSAMT1.

Response: Thanks very much for your comments. According to your suggestion, we measured the endogenous levels of BRs in wheat seedlings under freezing stress. We detected higher levels of endogenous BRs, including castasterone and 6-deoxocastasterone, in wild-type seedlings under cold stress (detailed in lines 332-335 and Fig. 5A, B in revised version). We performed ChIP assays with an anti-BZR1 antibody confirmed that TaBZR1 binds directly to the E-box motif-containing segments within the *TaSAMT1-B* promoter regions. Specifically, freezing stress treatment led to an enrichment of the R2 region containing the E-box motif from the *TaSAMT1-B* promoter in the precipitated chromatin, in line with *TaSAMT1-B* elevated transcriptional activation compared to mock treatment (detailed in lines 297-302 and Fig. 4E in revised version). Moreover, the levels of H3K9ac at the *TaSAMT1-B* promoter were significantly increased in *TaBZR1*-OE seedlings compared to the wild type under freezing treatment; H3K14ac levels were significantly higher in *TaBZR1*-OE seedlings than in the wild type under both control and freezing stress conditions (detailed in lines 437-441 and Fig. 7F,G in revised version). These results indicated that the overexpression of TaBZR1 triggers increased H3K9ac, H3K14ac and transcriptional upregulation of this loci under freezing stress. In addition, we observed an enrichment for TaBZR1 at the *TaSAMT1-B* promoter and increased H3K14ac levels over the *TaSAMT1* promoter following BL treatment relative to control seedlings (detailed in lines 444-449 and Fig. S13 in revised version). Notably, these changes in acetylation levels were not caused by changes in the affinity between TaBZR1 and TaHAG1, as determined by LCI assays in *N. benthamiana* leaves co-expressing *cLUC-TaBZR1* and *nLUC-TaHAG1* exposed to control or cold stress conditions (detailed in lines 450-453 and Fig. 7H in revised version). Taken together, these results indicate that TaBZR1 directly binds to the *TaSAMT1-B* promoter and further activates its transcription under cold stress by recruiting TaHAG1 and increasing the levels of histone H3 acetylation (detailed in Fig. 4; Fig. 7 and Fig. S13 in revised version).

2. Because of the problem in (1), BZR1-HAG1 interaction need to be tested by Co-IP, the golden standard for protein-protein interaction up to now.

Response: Thanks very much for your suggestion. In revised manuscript, we conducted co-immunoprecipitation (Co-IP) assays of protein extracts from *N. benthamiana* leaves expressing *TaBZR1-GFP*, *TaHAG1-MYC*, and the *GFP* alone via Agrobacterium (*Agrobacterium tumefaciens*)-mediated infiltration. Following an IP with an anti-GFP antibody, an immunoblot analysis with an anti-MYC antibody detected a band corresponding to TaHAG1-MYC in the samples co-expressing *TaBZR1-GFP* and *TaHAG1-MYC*, but not in those expressing *TaHAG1-MYC* and *GFP* (detailed in lines 372-378 and Fig. 6C in revised version). This result further confirmed that TaBZR1 can interact with TaHAG1.

Other comments:

1. Cold stresses include chilling and freezing. The authors used -10°C to treat the materials. This condition belongs to freezing. Replace cold with freezing in the manuscript.

Response: We are grateful for calling our attention to these inappropriate descriptions. We revised it, accordingly in the revised manuscript.

2. The authors claimed that they used Y1H to screen the transacting regulator of TaSAMT1, and used Y2H to screen the interaction partners of TaBZR1. However, no information on how the screening was performed. Add essential description on this work, including vector, cDNA library in Materials and Methods. The initial data showing the candidate proteins from screening should be mentioned in text.

Response: According to your suggestion, we further detailed the Y1H and Y2H screening information in section of Materials and Methods in the revised manuscript (detailed in lines 699-705 and lines 741-747 in revised version). The initial data of the candidate proteins from screening was presented in the revised manuscript, respectively (detailed in lines 260-264, lines 359-362 and Table S2, S3 in revised version).

3. The authors claimed that overexpression of *TaSAMT1* confers tolerance to cold

stress without the occurrence of discernible pleiotropic phenotypes and yield penalty. The authors drew the conclusion just based on a photograph (Fig S8) without any quantitative analyses on the yield in the field. More quantitative data are needed to support the conclusion if the authors want to keep this statement in manuscript. Otherwise, adjust the statement to avoid overstating the value of current data.

Response: Thanks very much for your suggestion. In the revised manuscript, we provided the quantitative data of agronomic traits of the *TaSAMT1* transgenic lines with wild-type plants, including plant height, spike length, spikelet number of per spike and thousand kernel weight. These results suggest that manipulation of *TaSAMT1* did not result in clear pleiotropic phenotypes or yield penalty under normal conditions but significantly increased freezing tolerance compared with wild-type plants (see lines 569-576 and Fig. S14 in revised version).

4. Line 130 and 136. In wheat genome, there are three homologs of TaSAMT1, *TaSAMT1-A*, *TaSAMT1-B* and *TaSAMT1-D*. Is *TraesCS3BG445100*, the *TaSAMT1-B*? Please clear it in text.

Response: Thanks very much for your comments. The gene of *TraesCS3BG445100* encodes TaSAMT1-B, and we present this information in the revised manuscript, according to your suggestion (see lines 159-165 in revised version).

5. qRT-PCR or RT-qPCR? Both are used in the manuscript. Should be RT-qPCR, not qRT-PCR.

Response: We are grateful for calling our attention to these inappropriate descriptions. We changed it to the RT-qPCR accordingly in the revised manuscript.

6. For description of the location of sequences, line 245, -679 to -387 bp (from left to right); line 331, -360 to -760 bp (from right to left). If not having special meaning for experiment designing, the same pattern should be used to avoid confusing readers.

Response: Thanks very much for your suggestion. We adjusted and uniformed the pattern in the revised manuscript (see line 409-410 in revised version).

7. Line 372-373, I really do not understand the following sentence: which carries...

platforms.

Response: We apologize for this inappropriate description that make it hard to understand. We revised it to “This question is particularly challenging in common wheat, which carries a large hexaploid genome with limited gene editing efficiency” in the revised manuscript (see line 464-466 in revised version).

8. Line 393-395, TaSAMT1 is involved in SA pathway upon .... From the author's work, the protein is involved in MeSA biosynthesis and may regulate the relative content of SA and MeSA. However, it is not known whether TaSAMT1 is directly involved in SA signaling pathway in wheat.

Response: Thank you for your comments. According to your suggestion, we revised it to “the SA methyl transferase TaSAMT1-B is involved in MeSA accumulation upon freezing stress and is essential for cold tolerance in wheat” in the revised manuscript (see line 488-489 in revised version).

9. Line 416, "tolerance in plant" need the or a before the word plant. Or just use the plural, plants.

Response: Many thanks for your suggestion. we revised it to “plants” in the revised version.

10. Line 417, "pathway" should be "pathways".

Response: We are grateful for calling our attention to these grammatical mistakes. We proofread and corrected accordingly in the revised manuscript.

11. Line 494, 10-day-old seedlings, 10 days after germination or 10 days after sowing? clear it.

Response: We are grateful for calling our attention to these missing information. 10-day-old seedlings refers to the 10 days after germination. We revised it in the new version (see line 591 in revised version).

12. Line 494, 1.5 h and and. The second "and" should be deleted.

Response: We greatly appreciate that these mistakes have been pointed out. We have carefully proofread and corrected the grammar and phrase errors accordingly in the

revised manuscript (all the revisions could be found in MS with Changes Highlighted version).

13. Line 495-496, how are surviving and died seedlings are judged? State the standard clearly.

Response: Thanks very much for your comments. We apologize for the inadequate description of the surviving judgement standard. According to your suggestion, we further detailed the information in section of Materials and Methods in the revised manuscript (see lines 592-594 in revised version).

14. Line 513-515, add the reference citation for the protoplast transformation, or describe the method in detail.

Response: Many thanks for your suggestion. We added the references in the revised manuscript (see lines 614 in revised version).

15. Line 546-547, Yuanye, Macklin and Coolaber are names of the company? if yes, add the name of the country to which the company belongs.

Response: We apologize for the inadequate description of the company information. According to your suggestion, we further detailed the country to which the company belongs in the revised manuscript (see lines 664-670 in revised version).

We would like to thank you again for the valuable recommendations and suggestions of the manuscript. Thank you again for your important contribution.

Sincerely,

Zhaorong Hu

Reviewer: 2

Low temperature stress is a major environmental stress types which limits plant performance and crop yields. Many crops are affected and among them is wheat, which can be damaged by freezing stress in several major growing areas of the world. In this work it was aimed to identify factors that can increase the frost tolerance of this crop plant. A novel mode of resistance against frost is revealed, which relies on the cold-induction of the salicylic acid (SA) methyltransferase TaSAMT1-B, that catalyzes MeSA production. Evidence is provided that this is carried out via the brassinosteroid signaling component TaBZR1 and the histone acyltransferase TaHAG1.

These are novel and significant findings since a SA methyltransferase has not been implicated in frost tolerance as yet. Also, it had been unknown that MeSA production can be induced by BR signaling components and that BZR1 takes part.

Response: We greatly appreciate your kind and positive evaluation on the research topic. Thanks very much for your suggestion, we further analyzed the data for some conclusions and the manuscript was revised in the new version, accordingly. The followings are our detailed responses to your comments.

Major comments:

1. Is there a BR context of TaBZR1 activity in the cold-induction of TaSAMT1 expression, like your model in Fig. 7 indicates? What's the evidence for it?

Response: Thanks very much for your comments. In revised manuscript, to check whether the BR pathway is involved in the cold response of wheat, we measured the endogenous levels of BRs in wheat seedlings under freezing stress. We detected higher levels of endogenous BRs, including castasterone and 6-deoxocastasterone, in wild-type seedlings under cold stress (see Fig. 5A,B in revised version). Moreover, relative *TaBZR1* transcript levels were upregulated after BL treatment, as were those of *TaSAMT1* (see Fig. S9A,B in revised version). Furthermore, we performed ChIP assays with an anti-BZR1 antibody and the ChIP-qPCR confirmed that TaBZR1 binds directly to the E-box motif-containing segments within the *TaSAMT1-B* promoter

regions. Specifically, freezing stress treatment led to an enrichment of the R2 region containing the E-box motif from the *TaSAMT1-B* promoter in the precipitated chromatin, in line with *TaSAMT1-B* elevated transcriptional activation compared to mock treatment (detailed in lines 297-302 and Fig. 4E in revised version). In addition, the levels of H3K9ac and H3K14ac at the *TaSAMT1-B* promoter were significantly increased in *TaBZR1*-OE seedlings compared to the wild type under freezing treatment (detailed in lines 437-441 and Fig. 7F,G in revised version). We also observed an enrichment for TaBZR1 at the *TaSAMT1-B* promoter and increased H3K14ac levels over the *TaSAMT1* promoter following BL treatment relative to control seedlings (detailed in lines 444-449 and Fig. S13 in revised version). Collectively, these results indicated the enhanced the BR content and TaBZR1 activity under cold treatment contributed to the cold-induction of *TaSAMT1* expression.

2. Are MeSA levels increased in TaBZR1oe and/or TaHAG1oe plants?

Response: Thanks very much for your comments. According to your suggestion, we measured the endogenous levels of MeSA in *TaBZR1*-OE and *TaHAG1*-OE plants. The result indicated endogenous level of MeSA content was significantly higher both in *TaBZR1*-OE and *TaHAG1*-OE lines than in wild-type plants, especially under freezing treatment (detailed in lines 326-327, lines 406-407 and Fig. S8, Fig. S12 in revised version), suggesting the overexpression of *TaBZR1* and *TaHAG1* improve the expression of *TaSAMT1* levels in turn result in accumulation of MeSA and enhanced resistance to freezing tolerance in wheat.

3. I don't see the E-box in the R2 promoter region of *TaSAMT1*. The region highlighted in red in Fig. 4A doesn't seem to contain one?!

Response: We are grateful for calling our attention to these inappropriate presentations. The BZR1 could bind to BRRE (CGTG(T/C)) and E-box (CANNTG) motifs in the promoters of target genes (see lines 274-275 and Fig. 4A in revised version). we re-highlighted the region in the revised version, accordingly.

4. The manuscript would benefit from language editing.

Response: Thanks very much for your comments. We apologize for the inadequate

description of the original manuscript. According to your suggestion, we further revised the manuscript completely via professional editing service.

We would like to thank you again for the valuable recommendations and suggestions of the manuscript. Thank you again for your important contribution.

Sincerely,

Zhaorong Hu

Reviewer: 3

In this study, Chu and colleagues identified TaSAMT1-B as a salicylic acid (SA) methyltransferase in wheat plants, which converts salicylic acid (SA) into MeSA. Examining connection of TaSAMT1 to abiotic stresses, they discovered that TaSAMT1 transcript levels are upregulated by cold temperature. This leads to more MeSA production to confer chilling tolerance in wheat plants. Interestingly, the authors discovered that wheat TaBZR1 transcription factor directly targets the upstream sequences of *TaSAMT1* to regulate gene expression. Mechanistically, TaBZR1 interacted with the histone acetyltransferase TaHAG1 and the resulting histone acetylation was enhanced by cold stress. Using genetic analyses, Chu and co-authors attempted to conclude that brassinosteroid (BR) signaling in wheat also mediates cold tolerance by modulating MeSA metabolism and homeostasis. Overall, this paper proposes that TaSAMT1 represents a metabolic/regulatory node for SA-BR hormone signaling crosstalk during cold stress responses in plants.

This study is interesting since, to my knowledge, this is the first time that a functional SAMT ortholog in wheat has been identified and characterized. Importantly, the authors tried to mechanistically link SA and BR signaling in cold tolerance responses, when previous studies only focused on the individual contribution of each plant hormone pathway. Taken together, this study should be of general interest to a broad readership, especially plant scientists working in abiotic stress, crop physiology, hormone signaling and gene regulation.

Response: Thank you very much for the evaluation. We greatly appreciate your kind and positive evaluation on our manuscript. We have performed additional experiments and revised our manuscript, accordingly. The followings are our detailed responses to your comments.

Major comments:

1. The authors performed biochemical characterization of TaSAMT1 activity and its ability to generate MeSA. They show MeSA standard as the positive control and empty expression vector as the negative control. Because the GC mass spectrum of

the MeSA standard is expectedly "cleaner" than the TaSAMT1-generated MeSA, it is important that more appropriate controls are used. For example, a good positive control would be using a confirmed plant SA methyltransferase (e.g. Arabidopsis BSMT1) and good negative controls would be the truncated homeologs TaSAMT1-A and TaSAMT1-C.

Response: Thanks very much for your comments. According to your suggestion, to test whether TaSAMT1 has enzymatic function, we produced and purified recombinant TaSAMT1-B fused to glutathione S-transferase (GST) in *E. coli*, together with GST alone, TaSAMT1-A-GST, and TaSAMT1-D-GST, using AtBSMT1-GST as a positive control. We then incubated crude bacterial protein extracts in the presence of SA to test methylation activity, revealing efficient catalysis of SA methylation with S-adenosyl-L-methionine (SAM) as a methyl donor for the cell lysates containing TaSAMT1-B or AtBSMT1, but not those with TaSAMT1-A or TaSAMT1-D, consistent with the absence of a complete carboxyl methyltransferase domain for these latter proteins (see lines 183-192 and Fig. 1D, Fig. S4 in revised version).

2. As shown in Figure 3, MeSA does not fully rescue the phenotypes of the KO lines in terms of survival rates and electrolyte leakage. It may be prudent if a range of MeSA concentrations could be used to determine which concentration leads to full rescue of KO lines, making them comparable to the WT Fielder. If this does not work, then potential reasons for the partially rescued phenotype should be indicated in the Discussion section.

Response: Thanks very much for your suggestion. In line with your suggestion, we also noted pre-treatment with MeSA (500 μM) could not fully rescue the phenotypes of the KO lines in terms of survival rates and electrolyte leakage to wild-type levels under freezing stress. We speculate the main reason for this phenomenon could be that the MeSA concentration (500 μM) might be not optimal in pre-treatment spray assays. In present study, we further assessed the effect of exogenous MeSA application on the freezing tolerance of *Tasamt1* mutants and wild-type plants. To this

end, we measured SRs and cell membrane stability in the wild-type Fielder and *Tasamt1* lines exposed to freezing conditions alone or together with a prior spray of MeSA at different concentrations. When 500 μM MeSA was applied before freezing treatment, the SR and cell membrane stability of both wild-type and *Tasamt1* plants significantly improved over those seen following freezing stress treatment alone. This response was dosage dependent, as an application of 1 mM MeSA fully rescued the SR and cell membrane stability defects seen in the *Tasamt1* mutants following freezing treatment, and comparable to wild-type Fielder under freezing stress. This finding is consistent with a role for TaSAMT1 in freezing tolerance that is dependent on MeSA accumulation.

(see lines 241-252 and Fig. 3 in revised version).

3. A major conclusion of the paper is that TaBZR1 directly targets and regulate *TaSAMT1* transcription. To robustly support this claim, I believe it is necessary to show in vivo BZR1 ChIP data.

Response: Thanks very much for your suggestion. In the revised manuscript, we performed the ChIP assays with antibodies against specific to TaBZR1 protein using the seedlings of wheat plants in control and freezing treatment. The precipitated chromatin DNA was analyzed by qPCR to examine enrichment levels relative to those of non-precipitated (input) genomic DNA. The ChIP-qPCR confirmed that TaBZR1 binds directly to the E-box motif-containing segments within the *TaSAMT1-B* promoter regions. Specifically, freezing stress treatment led to an enrichment of the R2 region containing the E-box motif from the *TaSAMT1-B* promoter in the precipitated chromatin, in line with *TaSAMT1-B* elevated transcriptional activation compared to mock treatment (detailed in lines 297-302 and Fig. 4E in revised version).

4. Another major conclusion is that exogenous BR rescues cold tolerance phenotypes of the KO lines. However, looking at Figure 5B, the increased survival rate by BL addition is only statistically significant in KO #1 but not in KO#2 and KO#3 lines. In addition, BL treatment has a lesser effect than MeSA treatment (shown in Figure 3).

To fully support their conclusions on the mechanistic connection between BR and SA in plant cold tolerance, and the requirement of BZR1 regulation, it is important to show the cold tolerance phenotypes and *TaSAMT1* gene expression profiles of the TaBZR1 KO mutant.

Response: Thanks very much for your suggestion. In line with the reviewer comments, we found exogenous BR treatment has a lesser effect than MeSA treatment and could not rescue cold tolerance phenotypes of the *TaSAMT1*-KO lines to wild type levels under freezing treatment (see Fig. 5C in revised version). These result suggested that *TaSAMT1* is required for the function of TaBZR1-involved BR pathway in freezing tolerance in wheat. To further support the conclusions on the mechanistic connection between BR and SA in plant cold tolerance, we identified a *Tabzr1* mutant by screening a mutant library in the KN9204 (a wheat variety) background. The mutant carries a G-to-A transition in the second exon of TraesCS2A02G187800, resulting in the introduction of a premature translation termination codon (Detailed in Fig. S7A in revised version). Immunoblot analysis with an anti-BZR1 antibody revealed a 50% decrease in TaBZR1 abundance in the *Tabzr1* mutant compared to the wild type (Detailed in Fig. S7B in revised version). We assessed the performance of the *Tabzr1* mutant lines under freezing stress conditions. When subjected to freezing treatment, the SR and cell membrane stability were significantly lower in *Tabzr1* mutant plants than that of wild-type (see lines 309-322 and Fig. 4J-L in revised version). We further assessed the expression levels of *TaSAMT1* in the *Tabzr1* mutant plants. The expression of *TaSAMT1* was downregulated in the *Tabzr1* mutant compared to the wild type (see lines 323-325 and Fig. 4ML in revised version). Together with the freezing tolerance phenotypes and *TaSAMT1* gene expression profiles in *TaBZR1*-OE lines, these results indicated TaBZR1 functions as a positive regulator in freezing tolerance of wheat and suggested that *TaSAMT1* could be an important target of TaBZR1 for freezing tolerance, which connecting the relationship between BR and SA pathways.

5. The authors also assert that BZR1-mediated cold tolerance occurs via

transcriptional regulation of TaSAMT1 and eventual MeSA generation. The current datasets are not fully sufficient to state this conclusion since one would need to cross and test a TaBZR1 KO line with the TaSAMT1 OX line. This would strongly show that the role of TaBZR1 in cold tolerance occurs through TaSAMT1 and not via other genes that it targets as a transcription factor.

Response: Thanks very much for your comments. The aforementioned data suggested that TaBZR1-mediated freezing tolerance occurs via transcriptional regulation of *TaSAMT1* and eventual MeSA generation. To further examine whether the TaBZR1-mediated BR signaling pathway regulates cold tolerance synergistically with the SA pathway via TaSAMT1 in wheat, we exposed wild-type, *TaSAMT1-B*-OE, and *Tasamt1* plants to freezing stress alone or following a prior spray with brassinolide (BL). The application of BL onto wild-type and *Tasamt1* seedlings before freezing stress treatment improved their survival rate and cell membrane stability compared to freezing stress treatment alone. Notably, the application of BL only partially alleviated the freezing sensitivity of *Tasamt1* seedlings and was not sufficient to return to wild-type levels (detailed in Fig. 5C-E in revised version), suggesting the TaSAMT1-B functions downstream of the BR pathway and is important for BR-mediated freezing tolerance. To test this hypothesis genetically, we crossed a *TaBZR1*-OE line (ox-2) to a *Tasamt1* mutant line (KO#3) and identified plants homozygous for the mutation of *TaSAMT1-B* and overexpressing *TaBZR1*. This *Tasamt1 TaBZR1*-OE line shared the same decreased SR and cell membrane integrity phenotypes as the *Tasamt1* mutant under freezing treatment (detailed in Fig. 5F-H in revised version), indicating that TaSAMT1-B functions downstream of TaBZR1 and modulates freezing tolerance. Collectively, these results suggest that TaSAMT1-B is required for the BR-regulated pathway and plays a vital role in freezing tolerance in wheat.

6. Finally, the authors state that cold conditions increase the histone acetylation of the *TaSAMT1* locus, but it is unclear whether this is due to enhanced interaction between TaBZR1 and TaHAG1 at colder temperatures and/or increased TaBZR1 recruitment to

the target *TaSAMT1* promoter region. I believe this needs more clarification to fully connect TaBZR1-TaHAG1 regulation to cold signaling/response.

Response: Thanks very much for your comments. According to your suggestion, we performed ChIP assays with an anti-BZR1 antibody and the ChIP-qPCR confirmed that TaBZR1 binds directly to the E-box motif-containing segments within the *TaSAMT1-B* promoter regions. Specifically, freezing stress treatment led to an enrichment of the R2 region containing the E-box motif from the *TaSAMT1-B* promoter in the precipitated chromatin, in line with *TaSAMT1-B* elevated transcriptional activation compared to mock treatment (detailed in lines 297-302 and Fig. 4E in revised version). Furthermore, the levels of H3K9ac and H3K14ac at the *TaSAMT1-B* promoter were significantly increased in *TaBZR1*-OE seedlings compared to the wild type under freezing treatment (detailed in lines 437-441 and Fig. 7F,G in revised version). In addition, we observed an enrichment for TaBZR1 at the *TaSAMT1-B* promoter and increased H3K14ac levels over the *TaSAMT1* promoter following BL treatment relative to control seedlings (detailed in lines 444-449 and Fig. S13 in revised version). Notably, these changes in acetylation levels were not caused by changes in the affinity between TaBZR1 and TaHAG1, as determined by LCI assays in *N. benthamiana* leaves co-expressing *cLUC-TaBZR1* and *nLUC-TaHAG1* exposed to control or cold stress conditions (detailed in lines 450-453 and Fig. 7H in revised version). Collectively, these results indicate that TaBZR1 directly binds to the *TaSAMT1-B* promoter and further activates its transcription under cold stress by recruiting TaHAG1 and increasing the levels of histone H3 acetylation (detailed in Fig. 4; Fig. 7 and Fig. S13 in revised version).

Minor comments:

1. Line 55 onward: In the Introduction (second paragraph), there needs to be better reflection of the current literature on cold temperature regulation of plant hormone signaling and metabolism. What is currently known in wheat and other plant species, especially in relation to the SA and BR pathways?

Response: We apologize for inadequate review of the current literature on cold temperature regulation of plant hormone signaling and metabolism. According to your suggestion, we further summarized the current literature in this field in revised manuscript (see lines 59-81 in revised version).

2. Lines 76-77: It is important to note that a recent study has shown systemic transport of SA via the apoplast (see Lim et al. 2020; https://doi.org/10.1126/sciadv.aaz0478).

Response: We are grateful for calling our attention to the recent study. According to your suggestion, we further revised the text and cited this literature, accordingly in the new manuscript (see lines 102-104 in revised version).

3. Lines 112-115: This could be restated for better clarity.

Response: Thanks very much for your suggestion. We revised it to "several studies have indicated that epigenetic modifications also have critical roles in coordinating environmental and BR signals" in the revised manuscript (see line 128-130 in revised version).

4. Materials and Methods: This section needs more information to facilitate future reproducibility. For example, please indicate the soil mix (and suppliers) and PPFD light settings for plant growth. Importantly, clearly outline the cloning steps (and kits used) for the following: (a) TaSAMT1 cloning to pCAMBIA1300-GFP, pGEX-6P-1, pLACZi2u and pGreen II 0800-LUC; (b) TaBZR1 cloning into pB42AD (pJG4-5), pUC18-35S and pCold-MBP; and (c) TaHAG1 cloning into pUC18-35S. Additionally, what protocol for protoplast isolation was used? Finally, please detail the protein purification steps as mentioned in the EMSA assay and in-vitro pull-down assay sections.

Response: Many thanks for your comments. According to your suggestion, we detailed the information in term of plant growth condition, cloning steps, protein purification in all assays, and protoplast isolation protocol, accordingly, in the revised manuscript (see section of Materials and Methods in revised version).

5. My comments focus on the science and not grammar, but I suggest that English

proofreading (perhaps using Grammarly or professional copyediting services?) is needed before publication.

Response: Many thanks for your suggestion. We further revised the manuscript completely via professional editing service (see in revised version).

Comments on Figures:

1. For all figure captions: Please indicate the number of reproducible independent experimental repeats performed for the various experiments.

Response: We are grateful for calling our attention to these missing information. We presented the number of reproducible independent experimental repeats performed for the various experiments in figure captions, respectively in the revised manuscript.

2. In addition, please clearly define n (are they individual plants? Individual leaves of the same plant? etc.)

Response: Many thanks for your comments. According to your suggestion, we detailed the sample information, accordingly in the revised manuscript (see line 758-759 and figure legend of Fig. 6d in revised version).

3. For Figure 1A/B; Figure 2A/E/F; Figure 3A/B/D/E; Figure 4D/G/G/H/I; Figure 5B/C; Figure 6E/F/G/H; Figure 7B/C/D; Figure S4C; and Figure S5; Figure S7A/B; and Figure S8B/C/D/E: For statistical transparency, please show the individual data points in all data graphs.

Response: Thanks very much for your suggestion. We presented the individual data points in all data graphs, respectively in the revised manuscript (see new figures in revised version).

4. Figure 1D: This needs a higher-resolution image.

Response: Many thanks for your comments. According to your suggestion, we further adjusted the figure to provide higher-resolution image in the revised manuscript (see Fig. 1D in revised version).

5. Figure 2B and Figure 4C: Quantification of band intensities needed.

Response: Thanks very much for your suggestion. We quantified the band intensities

in the revised version, accordingly.

6. Figure 2F left panel: Statistical analysis is missing.

Response: Many thanks for your comments. According to your suggestion, we further performed statistical analysis of the relative electrolyte leakage using t-test in Fig. 2F in the revised manuscript (see Fig. 2F in revised version).

7. Figure S1C: Please label the protein sequences with the catalytic site and other important protein domains.

Response: Thanks very much for your suggestion. We labeled the SAM-dependent carboxyl methyltransferase domain in the revised version (see Fig. S2B in revised manuscript).

8. Figure S2B: Please label the protein structures with the catalytic site and other important protein domains, highlighting what are missing in the A and D homeologs.

Response: According to your suggestion, we labeled the the SAM-dependent carboxyl methyltransferase domain and highlighted the missing domains, accordingly, in the revised manuscript (see Fig. S3 in revised version).

We would like to thank you again for the valuable recommendations and suggestions of the manuscript. Thank you again for your important contribution.

Sincerely,

Zhaorong Hu

Dear Dr. Zhaorong Hu:

A note from the Editor-in-Chief: First, thank you for your prompt and detailed response to my emailed request for more information and the investigation about the image duplication in Figure 4B. Your response supports that this was an honest mistake, which certainly happens. Below is the decision letter than we had written before my email, so it mentions the need for the negative controls, which I had also mentioned. It sounds like you understand how those controls should be designed, but let me know if anything is unclear.

I would also note that Reviewer 3 mentions sentences flagged by iThenticate as at risk for copying or plagiarism. You may want to use the same tool to see those results for yourself, and re-write the sentences that they mention, and others that the software catches.

We have received reviews of your manuscript entitled "The salicylic acid methyl transferase TaSAMT1 mediates freezing tolerance by integrating the brassinosteroid and salicylic acid pathways in wheat." On the basis of the advice received, the board of reviewing editors would like to accept your manuscript for publication in The Plant Cell. This acceptance is contingent on revision based on the comments of our reviewers. In particular, please consider the following:

Rephrase paragraphs that resemble text from previously published manuscripts. Revise the text according the requests of Reviewer #1. Finally, in addition to the reviewers comments, the LCI and BiFC experiments in Fig. 6A and D are lacking the appropriate negative controls - please repeat these with the appropriate negative controls.

Please highlight all changes and include a detailed annotation of changes of the text, with line numbers, and noting your responses to the comments.

To submit your revised manuscript, click:

Link Not Available

If you have any questions about the revision submission procedures, please contact the Editorial Office Staff (tpc-submissions@aspb.org). If you cannot return the revised manuscript within 30 days, please let us know. Otherwise, we will assume that you have elected not to revise the manuscript and withdraw it.

Thank you very much for the privilege of reviewing this work. I look forward to receiving the next version.

Sincerely,

The Plant Cell Board of Editorsl

Reviewer #1 (Comments for the Author):

The authors added some new data to support their conclusion in this version, and answered all questions asked in the previous version. Thus I agree to publish it after some revision.

1. Line 153, delete "putative". The data provided here indicated that TaSAMT1/TaSAMT1-B/TraesCS3B02G445100 is a salicylate carboxyl methyltransferase.
2. Line 306-309, "freezing stress treatment led to an enrichment of the R2 region containing the E-box motif from the TaSAMT1-B promoter in the precipitated chromatin, in line with TaSAMT1-B elevated transcriptional activation compared to mock treatment (Fig. 4E)". please note, in Figure 4E, control is higher than freezing treatment according to the column color.

Reviewer #2 (Comments for the Author):

The authors have addressed all of my concerns. It is a very interesting paper, which I look forward to seeing published.

Reviewer #3 (Comments for the Author):

Dear Authors/Editor,

I commend Chu and co-authors for the tremendous achievement and meticulous work in conducting additional experiments that further strengthened this manuscript. The authors have excellently addressed all my major technical and conceptual concerns/suggestions as a reviewer. Thank you very much!

This study successfully linked SA and BR signaling in cold tolerance responses, when previous studies only focused on the individual contribution of each plant hormone. As I had stated previously, this work should be of general interest to a broad readership, especially plant scientists working in abiotic stress, crop physiology, hormone signaling and gene regulation.

Minor comment: I did run the text through iThenticate and I would caution the authors to carefully paraphrase the following lines that highly resemble previously published material/papers:

1) Lines 54-56: Highly comparable to an Intro sentence in Jiang et al. 2022 (https://doi.org/10.3389/fpls.2022.811884)

2) Lines 59-61: Highly comparable to an Abstract sentence in Raza et al. 2023 (https://doi.org/10.1016/j.stress.2023.100152)

3) Lines 70-74: Highly comparable to Abstract and Discussion sentences in Castroverde et al. 2021 (https://doi.org/10.1093/jxb/erab257)

4) Lines 74-79: Highly comparable to a Conclusion sentence in Shi et al. 2014 (https://doi.org/10.1093/pcp/pcu115)

5) Lines 104-105: Highly comparable to a Conclusion sentence in Lim et al. 2020 (https://doi.org/10.1126/sciadv.aaz0478)

6) Lines 111-113: Highly comparable to a sentence in the review article by Gondor et al. 2022 (https://doi.org/10.1016/j.jplph.2022.153809)

7) Lines 119-122; 128-129: Highly comparable to a sentence in the review article by Nolan et al. 2020 (https://doi.org/10.1105/tpc.19.00335)

8) Lines 128-129: Identical to an Introduction sentence in the review article by Gondor et al. 2022 (https://doi.org/10.1016/j.jplph.2022.153809)

9) Lines 483-483: Highly comparable to a Discussion sentence in Kim et al. 2022 (https://doi.org/10.1016/j.molp.2022.05.002)

10) Lines 503-505; 510-511: Highly comparable to a sentence in the review article by Gondor et al. 2022 (https://doi.org/10.1016/j.jplph.2022.153809)

11) Lines 517-518: Highly comparable to a sentence in the review article by Nolan et al. 2020 (https://doi.org/10.1105/tpc.19.00335)

12) Lines 538-540; 550-552: Highly comparable to an Introduction sentence in Song et al. 2022 (https://doi.org/10.1111/nph.18372)

13) Lines 576-578: Highly comparable to a sentence in the review article by Nolan et al. 2020 (https://doi.org/10.1105/tpc.19.00335)

14) Lines 590-593: Highly comparable to a Discussion sentence in Zheng et al. 2021 (https://doi.org/10.1093/plphys/kiab187)

15) Lines 611-618: Highly comparable to Methods text in Li et al. 2017 (https://doi.org/10.1016/j.molp.2017.01.004)

Please also note the following:

-The Plant Cell now requires authors to complete and submit an author revisions checklist upon submission of a revised manuscript. The aim of the checklist is to aid authors in preparing a high-quality manuscript, facilitate the review and assessment of revised manuscripts, and help to ensure that journal standards are maintained across the board. If your manuscript is accepted, the completed checklist will be published as supplemental material attached to the article online. Please

download a copy of the checklist (pdf fillable form) at this link, for submission with your revised manuscript: https://tpc.msubmit.net/html/Author_Revisions_Checklist.pdf.

-Supplemental materials should be restricted to large datasets and tables, presentation of replicates, and validation of reagents, methods, or genotypes. Any data that are used to support the major claims must be in the main manuscript. Supplemental figure legends must indicate what figure in the main manuscript is supported by the supplemental data presented. Please justify how each of the supplemental figures meet the criteria.

-Sampling methods and the nature of "biological replicates" should be described precisely (i.e. different plants, parts of plants, pooled tissue, independent pools of tissue, sampled at different times, etc.), along with a clear description of and rationale for any statistical analyses conducted. The reader should know exactly what was sampled; what forms the basis of the calculation of any means and statistical parameters reported. This is also necessary to ensure that proper statistical analysis was conducted.

Dear Editors and Reviewers,

We would like to thank you and the reviewers for the helpful comments and invaluable suggestions on our manuscript, which help us a lot to improve the quality of the manuscript for publication in the Plant Cell. We would like to express our gratitude for your time and work. The following responses have been provided to address all comments in detail.

Response to the editors and reviewers point-by-point:

In the revised manuscript, all the raised issues have been addressed and revised according as requested by the editors and reviewers. The the negative controls for the LCI and BiFC experiments in Fig. 6A and D were performed and presented in Fig. S10B and C in the revised version. We apologize again for the mistake in Fig. 4B and we are very grateful that this mistake has a chance to be corrected in time. The Fig. 4B and E were revised, accordingly. In addition, the manuscript was checked throughout and the sentences flagged by the software were paraphrased or quoted appropriately.

Reviewer comments:

Reviewer #1:

The authors added some new data to support their conclusion in this version, and answered all questions asked in the previous version. Thus I agree to publish it after some revision.

1. Line 153, delete "putative". The data provided here indicated that TaSAMT1/TaSAMT1-B/TraesCS3B02G445100 is a salicylate carboxyl methyltransferase.

Response: We greatly appreciate your comments and recommendations of the manuscript. According to your suggestion, we deleted the "putative" in the revised manuscript (see line 153 in revised version).

2. Line 306-309, "freezing stress treatment led to an enrichment of the R2 region containing the E-box motif from the TaSAMT1-B promoter in the precipitated chromatin, in line with TaSAMT1-B elevated transcriptional activation compared to mock treatment (Fig. 4E)". please note, in Figure 4E, control is higher than freezing treatment according to the column color.

Response: We are grateful for calling our attention to these column color mistakes.

We proofread and corrected accordingly in the revised manuscript (see in Fig 4E in the revised version).

Reviewer #2:

The authors have addressed all of my concerns. It is a very interesting paper, which I look forward to seeing published.

Response: We greatly appreciate your kind and positive evaluation on the research topic. Thanks very much for your help and suggestion.

Reviewer #3:

I commend Chu and co-authors for the tremendous achievement and meticulous work in conducting additional experiments that further strengthened this manuscript. The authors have excellently addressed all my major technical and conceptual concerns/suggestions as a reviewer. Thank you very much!

This study successfully linked SA and BR signaling in cold tolerance responses, when previous studies only focused on the individual contribution of each plant hormone. As I had stated previously, this work should be of general interest to a broad readership, especially plant scientists working in abiotic stress, crop physiology, hormone signaling and gene regulation.

Minor comment: I did run the text through iThenticate and I would caution the authors to carefully paraphrase the following lines that highly resemble previously published material/papers:

Response: We greatly appreciate your important comments and positive evaluation of the manuscript. We are grateful for your contribution, which help us a lot to improve the quality of the manuscript. Thank you very much for calling our attention to these sentences flagged as at risk for copying. We checked the manuscript throughout and the sentences flagged by the software were paraphrased or quoted appropriately (see in the revised version of the manuscript).

We would like to express our deepest gratitude to the editors and reviewers for the comments and suggestions to the manuscript. Thank you again for your important contribution.

Sincerely yours,

Zhaorong Hu

Dear Dr. Zhaorong Hu:

We are pleased to inform you that your paper entitled "The salicylic acid methyl transferase TaSAMT1 mediates freezing tolerance by integrating the brassinosteroid and salicylic acid pathways in wheat" has been accepted for publication in The Plant Cell, pending a final editorial review by a science editor. At this stage, your manuscript will be evaluated by a science editor with respect to its presentation of scientific content, compliance with journal policies, and presentation for a broad readership. The Plant Cell has contracted with Plant Editors (planteditors.com) to provide this service to our authors, and you will soon receive additional information on this process.

Please note that each author needs to link their ORCID identifier to their account in the system before your manuscript can be published. If any authors do not have an ORCID linked to their account, they will receive a message with a link to complete this task. Please ensure that ALL of your coauthors have completed this task as soon as possible.

ASPB offers an OPEN option that allows authors to have their online articles available for free to all users immediately upon publication. For more information about the ASPB OPEN option, refer to the Final Submission Checklist Form.

The Plant Cell and The Arabidopsis Information Resource (TAIR) are collaborating to collect functional annotation data about Arabidopsis genes from authors. This includes information about the gene's molecular function (e.g., kinase activity, ATP synthetase activity), the biological process/es it is involved in (e.g., endosperm development, threonine biosynthesis), its subcellular location (e.g., nucleus, ER), anatomical or developmental expression pattern (e.g., leaf, ovule, flower stage 10, seedling stage), or its partner in a protein-protein interaction (e.g., AT1G01010 interacts with AT1G01020).

If your paper contains results falling into one or more of these categories for Arabidopsis genes, we request that you now submit these data for inclusion in TAIR by filling in the form provided at the following URL: https://www.arabidopsis.org/doc/submit/functional_annotation/123. If you need further clarification on what types of data can be submitted please contact curator@arabidopsis.org.

Finally, we encourage your submission of artwork for the journal cover. Monthly issues will have an online cover image and selected covers will be used for posters, other promotional items, and "wallpaper" for mobile devices. For more information, refer to Cover Submission in the Instructions for Authors https://tpc.msubmit.net/cgi-bin/main.plex?form_type=display_auth_instructions.

We look forward to seeing your paper published.

Sincerely,

The Plant Cell Board of Editors

=====
IMPORTANT REMINDER: PEER REVIEW REPORTS
=====

If you opted to publish a peer review report along with your article during the original submission process, it will be prepared by the editorial staff and publicly posted with your manuscript, inside the zip file that contains any other supplemental material. As a reminder, the peer review report is a public record of all comments from editors and reviewers, as well as your prior responses, as you received them in the decision letters for each draft of your manuscript. If you agreed to publish this report and have changed your mind, or are not sure if you selected this option, please contact the editorial office as soon as possible before signing the license agreement from our publisher.

=====
=====

----FOR ASPB OFFICE USE ONLY (DO NOT EDIT)----
MSID: 37895
Corresponding Author: Prof. Hu
